# Four subtypes of disease-causing missense mutations underlie pathogenic protein interactions in neurodegenerative *VPS13A* disease

**DOI:** 10.1172/JCI200890

**Published:** 2026-03-24

**Authors:** Xing Lin, Yuta Ryoden, Chigure Suzuki, Hiroyuki Ishikawa, Takaharu Sakuragi, Yasuo Uchiyama, Shigekazu Nagata

**Affiliations:** 1 Laboratory of Biochemistry and Immunology, Immunology Frontier Research Center, The University of Osaka, Suita, Osaka, Japan.; 2 Department of Cellular and Molecular Pharmacology, and; 3 Department of Cellular and Neuropathology, Graduate School of Medicine, Juntendo University, Tokyo, Japan.; 4 Center for Infectious Disease Education and Research, The University of Osaka, Suita, Osaka, Japan.

**Keywords:** Genetics, Neuroscience, Neurodegeneration

## Abstract

VPS13A is an intracellular lipid transfer protein comprising more than 3,000 amino acids. Mutations in human VPS13A cause *VPS13A* disease, a neurodegenerative disorder that affects movement and cognition. VPS13A forms a complex with the membrane protein XK to mediate ATP-induced phospholipid scrambling in the plasma membrane. Here, we established a mouse cell system expressing full-length mouse VPS13A and examined its interaction with XK. Mutational analysis revealed that VPS13A binds to XK through a C-terminal β-strand that interacts with a β-hairpin in the central region of XK, an interaction essential for scramblase activity. The XK paralog XKR2, which contains a similar β-hairpin structure, also associates with VPS13A and supports phospholipid scrambling. We analyzed 10 mouse VPS13A variants corresponding to human patient mutations and classified them into 4 groups: (a) L67P, I90K, and W2453R, which showed reduced expression; (b) A1091P and M3080R, which were normally expressed but lacked scramblase activity; (c) S1446P, Q2689H, Y2713C, and R3084H, which modestly impaired expression or activity; and (d) I2763R, which altered cell size and disrupted ER independently of XK. These findings define the VPS13A–XK interaction interface, clarify the functional impact of disease-causing mutations, and reveal an unexpected gain-of-function mutation of a VPS13A variant.

## Abstract 2

In this study, we propose a model that explains how the neurodegenerative disease chorea-acanthocytosis develops.

## Graphical abstract

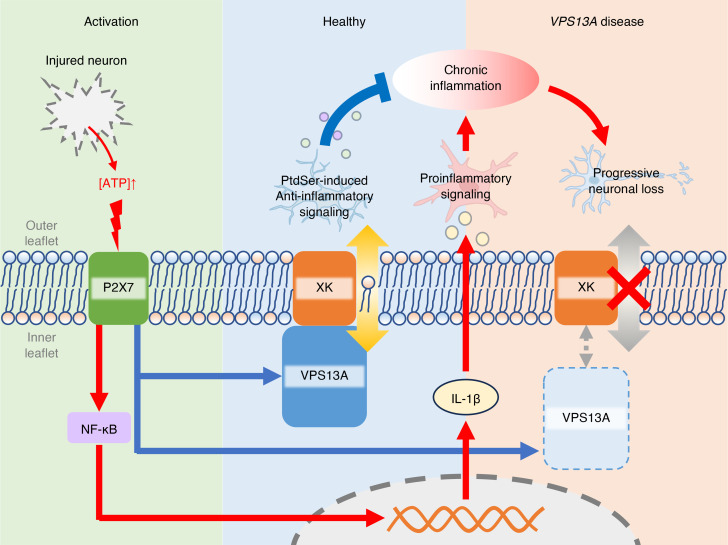

## Introduction

Vacuolar protein sorting 13 homolog A (VPS13A) is a large cytoplasmic protein that belongs to the bridge-like lipid transfer protein family. It is thought to mediate the directional transfer of phospholipids between organelles, including ER, mitochondria, plasma membrane, and lipid droplets (1, 2). Human VPS13A consists of 3,174 amino acids and contains an N-terminal chorein domain and an extensive β-sheet region with a highly hydrophobic interior (3). Its C-terminal region harbors several domains: a VPS13 adaptor-binding domain, aberrant pollen transmission 1 domain, autophagy-related protein C-terminal domain, and pleckstrin homology domain (PH), which mediate interactions with target membranes or adaptor proteins (3, 4). Loss-of-function mutations in the *VPS13A* gene, including deletions and missense variants, cause *VPS13A* disease (historically referred to as chorea-acanthocytosis), a rare autosomal recessive neurodegenerative disorder (2, 5).

In eukaryotic cells, the plasma membrane comprises a lipid bilayer that separates the cell from its environment. Organelles such as ER, nucleus, mitochondria, and lysosomes are enclosed by lipid bilayers that isolate their lumens from the cytosol (6). The major constituents of lipid bilayers include phospholipids such as phosphatidylcholine, phosphatidylethanolamine, phosphatidylserine (PtdSer), phosphatidylinositol, and sphingomyelin (7). Glycerophospholipids are synthesized in the ER (8), whereas most sphingomyelin is synthesized in the Golgi apparatus (9). Lipid composition varies between plasma membranes and intracellular organelles (6). Because phospholipids are amphiphilic, they spontaneously form bilayers with inwardly oriented hydrophobic acyl chains. Variations in acyl chain length and unsaturation influence membrane curvature (7). Cells exploit these properties by asymmetrically distributing phospholipids between the 2 leaflets of the bilayer (10, 11) to maintain membrane integrity and function.

In the plasma membrane, PtdSer and phosphatidylethanolamine are usually confined to the inner leaflet, a distribution maintained by P4-type ATPases ATP11A and ATP11C, which actively flip these phospholipids inward in an ATP-dependent manner (11–13). This asymmetry is disrupted in processes such as apoptosis, blood clotting, cell fusion, and inflammation, during which PtdSer exposure on the cell surface serves as a signaling cue or enzyme activator (11, 14). Phospholipid scrambling, the bidirectional, nonselective translocation of lipids between bilayer leaflets, is mediated by scramblases. Three types of plasma membrane scramblases have been identified: TMEM16F, XKR8, and TMEM63B (11, 15, 16). TMEM16F functions as a Ca²^+^-dependent scramblase responsible for PtdSer exposure in activated platelets (17), whereas XKR8 acts as a caspase-dependent scramblase that mediates PtdSer exposure during apoptosis (18).

Although extracellular ATP concentrations are typically low (~30 nM) compared with intracellular levels (~4 mM) (19), ATP can reach hundreds of micromolar levels in inflamed tissues or tumors due to its release from necrotic cells. In these settings, ATP binds to the P2X7 receptor on CD25^+^ regulatory T cells and macrophages, triggering PtdSer exposure (20, 21). In a CRISPR–Cas9 screen for genes involved in ATP-induced PtdSer exposure in T cells, we previously identified the VPS13A-XK complex as a scramblase activated downstream of ATP-engaged P2X7 (22). The shared clinical features of *XK* disease (McLeod syndrome) and *VPS13A* diseases prompted several groups to report the physical and functional interactions between XK and VPS13A (23–26). Furthermore, by transiently expressing internally Halo- or mCherry-tagged human VPS13A or a GFP-tagged C-terminal fragment of VPS13A in COS or HEK293T cells, Park et al. (24) and Guillén-Samander et al. (25) showed that VPS13A interacts with XK via the C-terminal PH domain.

In this study, we established stable mouse cell lines expressing intact mouse VPS13A and demonstrated that its complex formation with XK is required for ATP-stimulated lipid scrambling. We also identified mouse XKR2, a previously identified orphan XKR family member, as a VPS13A-binding partner that supports lipid scrambling. Finally, using stable transformants expressing mouse VPS13A mutants corresponding to patient-derived missense variants, we found that 3 mutations impaired protein synthesis or stability, whereas 2 specifically compromised PtdSer-scramblase activity. Notably, 1 mutant not only reduced protein expression and scramblase activity, it also induced a novel phenotype characterized by enlarged cell size and delayed growth, revealing an additional XK-independent role for VPS13A in cellular physiology.

## Results

### VPS13A–XK complex in ATP-induced PtdSer exposure.

Park et al. (24) previously reported an elongated VPS13A structure predicted by AlphaFold 2.1, in which 4 overlapping VPS13A segments were individually modeled and assembled into a full-length structure using ChimeraX. The XK model, also predicted by AlphaFold, was aligned with the PH region of VPS13A.

**Figure 1:**
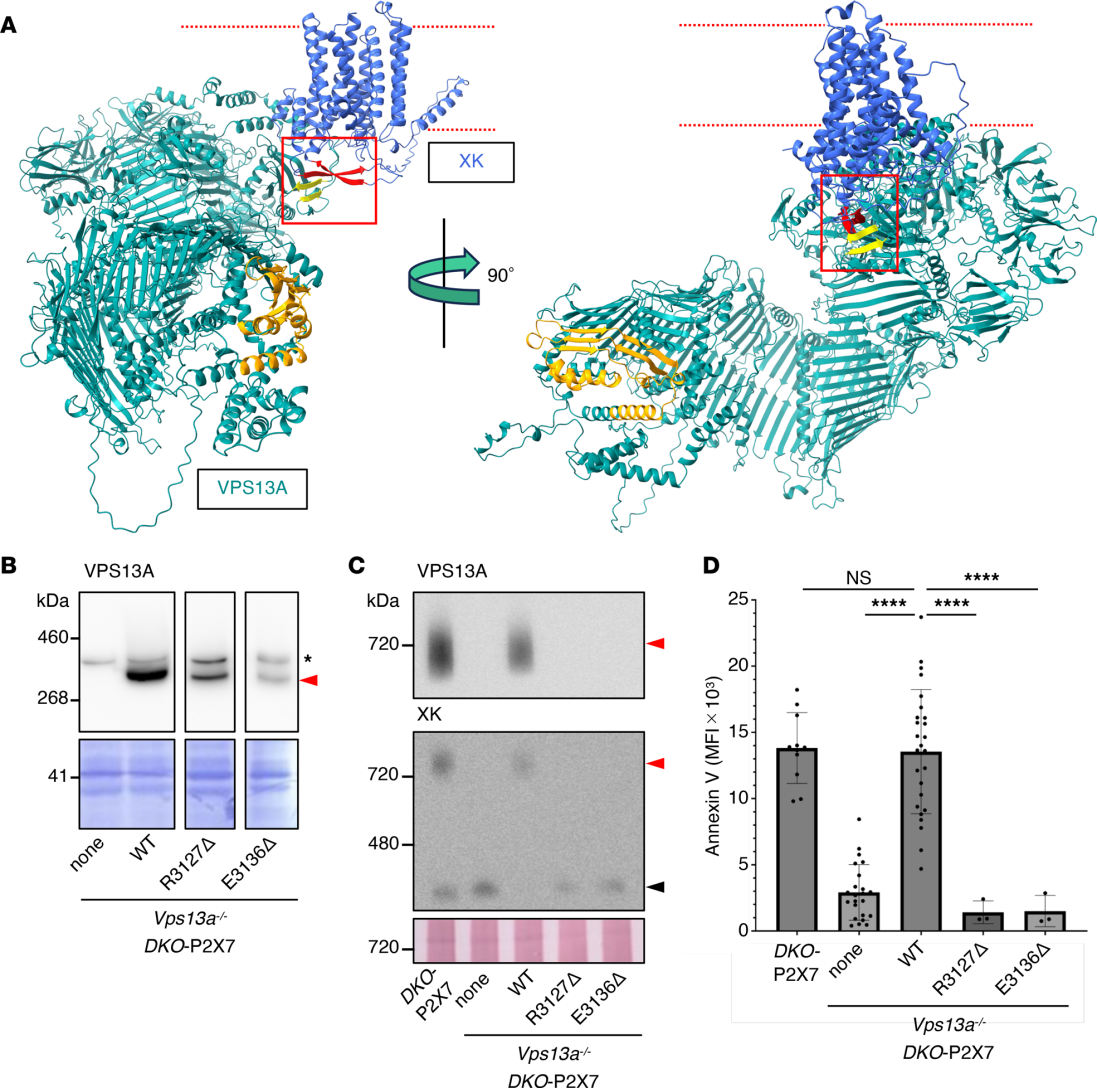
Requirement of the C-terminal domain of mouse VPS13A for ATP-induced PtdSer exposure. (**A**) AlphaFold 3-predicted mouse VPS13A–XK structure. The structure of the VPS13A-XK complex was predicted using AlphaFold 3 (27) with interface predicted template modeling score (ipTM) = 0.72 and predicted template modeling score (pTM) = 0.54, and viewed at 2 angles. VPS13A is shown in dark cyan and XK in royal blue. The N-terminal chorein domain of VPS13A is shown in orange. The β-hairpin of XK is shown in red, and the 2 β-strands in the PH domain of VPS13A are shown in yellow. The red dotted lines indicate the predicted membrane bilayers. The region where β-hairpin of XK interacts with β-strands of the PH domain of VPS13A is enclosed by a square line. (**B–D**) Effect of VPS13A C-terminal deletion mutants on their interaction with XK and ATP-induced PtdSer exposure. WT, R3127Δ, and E3136Δ VPS13A were stably expressed in *Vps13a^–/–^DKO*-P2X7 cells. (**B**) Whole-cell lysates were separated by SDS-PAGE and analyzed by Western blotting using anti-VPS13A Ab. Red arrowhead indicates VPS13A; * indicates a nonspecific band. (**C**) Membrane fractions from *DKO*-P2X7, *Vps13a^–/–^DKO*-P2X7, and their transformants expressing none, WT, R3127Δ, or E3136Δ mouse VPS13A were separated by BN-PAGE and analyzed by Western blotting with anti-VPS13A (upper) or anti-XK (lower) Abs. Red arrowheads indicate the VPS13A–XK complex; the black arrowhead indicates XK. The band intensity for XK in WT*-Vps13a^–/–^DKO*-P2X7 is slightly lower than that observed in *DKO*-P2X7 with unknown reason. (**D**) *DKO*-P2X7, *Vps13a^–/–^DKO*-P2X7, and their transformants expressing none, WT, R3127Δ, or E3136Δ mouse VPS13A were treated with ATP. PtdSer exposure was assessed by flow cytometry using Cy5–annexin V and expressed as MFI. Data are presented as mean ± SD (bars) of at least 3 independent experiments. The statistical significance was determined using 1-way ANOVA with Dunnett’s multiple comparison test. ****P < 0.0001.

AlphaFold 3 has increased the size limit for structure prediction to 5,000 amino acids and enables the modeling of protein complexes (27), allowing us to predict the structure of full-length VPS13A in complex with XK. When mouse VPS13A and XK sequences were submitted to the AlphaFold 3 server, the resulting complex structure (Figure 1A) differed markedly from the human VPS13A reported by Park et al. (24). The predicted VPS13A structure was more compact and bent, suggesting that N- or C-terminal truncations may adopt conformations distinct from those of the intact protein. Moreover, the AlphaFold 3–predicted structure differed substantially from the cryo-EM structure of VPS13C reported by Li et al. (28). Because purified VPS13C forms a complex with calmodulin, this interaction may account for the observed structural differences.

GFP-tagged human VPS13A (hVPS13A^GFP), with GFP inserted between codons 1359 and 1360, has been shown to complement *Vps13a* deficiency in yeast (29). The mouse T cell lymphoma line WR19L grows well in suspension and is well-suited for flow cytometric analysis. We previously reported that *Tmem16f^–/–^P2rx7^–/–^*WR19L cell (*DKO*) transformants expressing P2X7 respond to treatment with 500 μM ATP by exposing PtdSer within 5 minutes at 4°C in a VPS13A-dependent manner (Supplemental Figure 1; supplemental material available online with this article; https://doi.org/10.1172/JCI200890DS1) (22). Stable expression of full-length human or mouse VPS13A under the control of the human *EF-1α* promoter rescued scramblase activity, whereas hVPS13A^mCherry did not (Supplemental Figure 1).

To elucidate the molecular mechanism by which the VPS13A–XK complex supports phospholipid scrambling, we generated stable cell lines expressing WT or mutant VPS13A or XK in *Vps13a*-null or *Xk*-null cell lines.

### Interaction between XK’s β-hairpin and the C-terminal domain of VPS13A.

XK, but not XKR8, contains a β-hairpin in its central cytoplasmic region (22), a motif that often mediates protein-protein interactions (30, 31). Two groups (24, 25) demonstrated that this β-hairpin binds to the C-terminal domain of human VPS13A using transient overexpression in COS or 293T cells of GFP-tagged truncated VPS13A or internally mCherry- or Halo-tagged VPS13A.

To test the role of this interaction in scramblase activity, we expressed WT mouse VPS13A or C-terminally truncated mutants (R3127* and E3136Vfs*6; hereafter referred to as R3127Δ and E3136Δ, respectively), corresponding to patient variants (32, 33), in *Vps13a^–/–^DKO-*P2X7 cells. SDS–PAGE analysis showed that the mutant proteins were stably expressed, although at lower levels than WT mouse VPS13A (Figure 1B). Blue native (BN)-PAGE detected WT VPS13A at approximately 700 kDa in membrane fractions, which was absent in nontransformants and transformants expressing the C-terminally truncated VPS13A (Figure 1C). Immunoblotting with anti-XK Ab detected a 250 kDa band in VPS13A-nontransformed cells. Given that detergent-solubilized membrane proteins often migrate larger than their expected size in BN-PAGE (34), this band likely represents the monomeric form of mouse XK protein. In cells expressing WT but not mutant VPS13A, mouse XK displayed an additional band at approximately 720 kDa, which likely represents the VPS13A-XK complex (Figure 1C).

When these transformants were treated with ATP, only those expressing WT mouse VPS13A showed PtdSer exposure (Figure 1D), demonstrating that the C-terminal 31 residues of VPS13A are essential for its expression and scramblase activity in the plasma membrane. Structural modeling suggests that the C-terminal PH region of 100 amino acids (position 3067–3166) forms a knob-like structure comprising 1 α-helix segment (position 3146–3160) and 2 clusters of β-strands (β-α-β) stabilized by hydrophobic contacts (V3071 with I3152; I3114 and L3120 with L3153) (Supplemental Figure 2).

**Figure 2:**
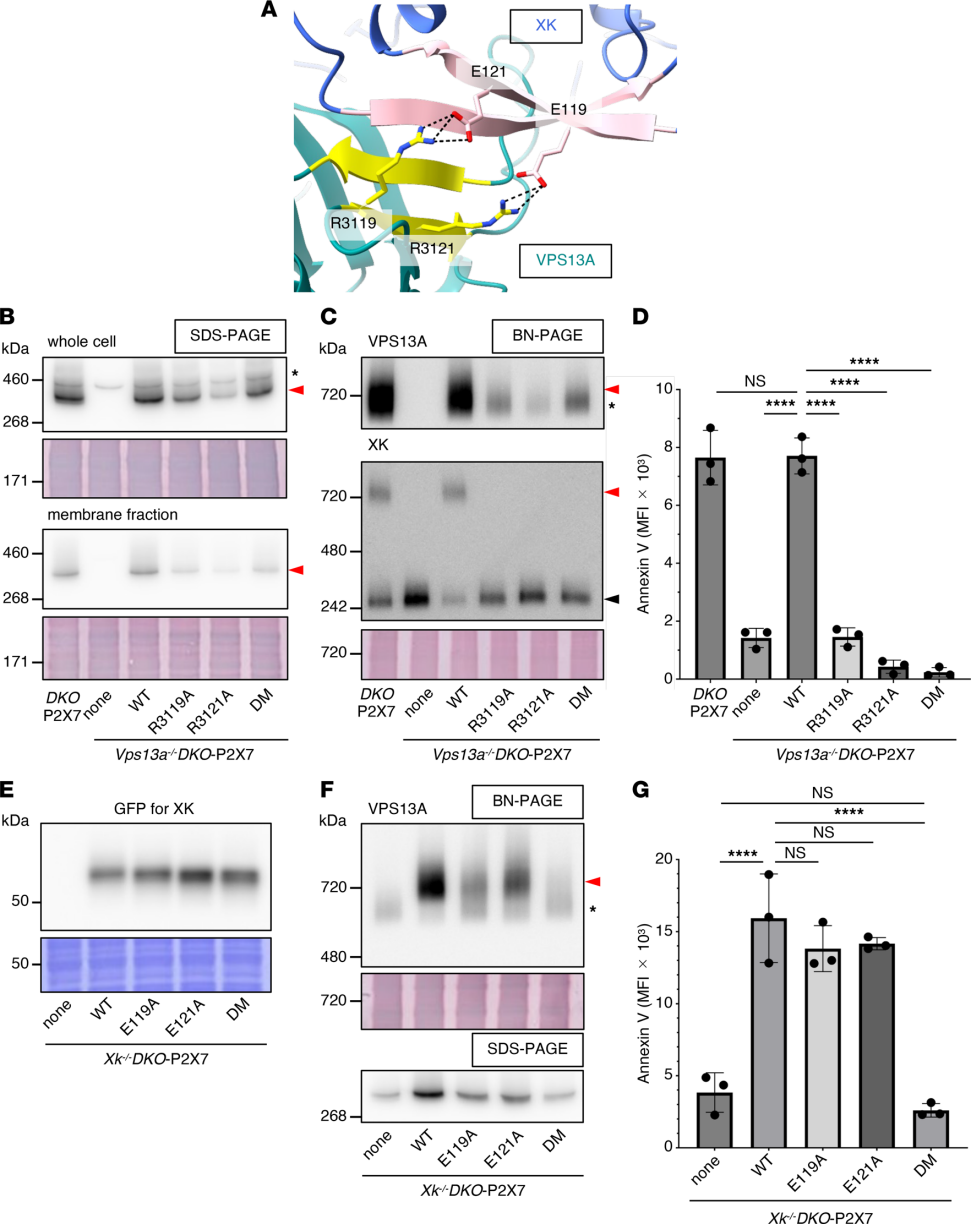
Interaction between mouse VPS13A and XK. (**A**) The red boxed region in Figure 1A is shown in an enlarged view. Two glutamates (E119 and E121) in the XK β-hairpin form hydrogen bonds (dotted lines) with R3119 and R3121 in the VPS13A C-terminal β-sheet. (**B–D**) Effect of VPS13A missense mutations on XK binding and scramblase activity. *Vps13a^–/–^DKO*-P2X7 cells were transformed with WT, R3119A, R3121A, or R3119A/R3121A DM mouse VPS13A. (**B**) Whole-cell lysates (upper panel) and the membrane fractions (lower panel) were separated by SDS-PAGE and immunoblotted with anti-VPS13A Ab. (**C**) Membrane fractions were separated by BN-PAGE and immunoblotted with anti-VPS13A Ab (upper panel) or anti-XK Ab (lower panel). Red arrowheads indicate the VPS13A-XK complex. The band marked with an asterisk indicates an unidentified protein; the black arrowhead indicates the uncomplexed XK. (**D**) Cells were stimulated with ATP, and PtdSer exposure was analyzed by flow cytometry using Cy5–annexin V for MFI. Data are presented as mean ± SD (bars) of 3 experiments. (**E–G**) Effect of XK missense mutations on VPS13A binding and scramblase activity. *Xk^–/–^DKO*-P2X7 cells were transformed with WT, E119A, E121A, or E119A/E121A DM mouse XK. (**E**) Whole-cell lysates were separated by SDS-PAGE and immunoblotted with anti-GFP Ab. (**F**) Membrane fractions were separated by BN-PAGE (upper panel) or SDS-PAGE (lower panel) and immunoblotted with anti-VPS13A Ab. Arrowheads indicate the VPS13A-XK complex. The band marked with an asterisk indicates an unidentified protein. (**G**) Cells were stimulated with ATP, and PtdSer exposure was analyzed by flow cytometry using Cy5–annexin V for MFI. Data are presented as mean ± SD (bar) of 3 experiments. In (**D** and **G**), statistical significance was determined using 1-way ANOVA with Dunnett’s (**D**) or Šídák’s (**G**) multiple comparison test. ****P < 0.0001.

A close examination of the interaction interface between mouse XK and VPS13A revealed that the XK’s β-hairpin interacts with β-strands in the C-terminal region of VPS13A through specific hydrogen bonds, with mouse XK residues E119 and E121 engaging with mouse VPS13A residues R3121 and R3119, respectively (Figure 2A). To assess the functional importance of these interactions, mouse VPS13A residues R3119 and/or R3121 were mutated to alanine (Ala) and expressed in *Vps13a*-null *DKO-*P2X7 cells. Conversely, mouse XK residues E119 and/or E121 were mutated to Ala in C-terminally monomeric (m)EGFP-tagged XK and introduced into *Xk*-null *DKO-*P2X7 cells. As shown in Figure 2, B and E, mutant VPS13A and XK proteins were expressed comparable to those of their respective WT counterparts except for R3121A mutant. In addition, the plasma membrane localization of XK mutants was indistinguishable from that of WT XK (Supplemental Figure 3).

BN-PAGE and functional analyses demonstrated that a single Ala substitution of either VPS13A residue R3119 or R3121 markedly impaired VPS13A-XK complex formation in the membrane fraction (Figure 2, B and C) and abolished ATP-induced PtdSer exposure (Figure 2D). In contrast, single E119A or E121A mutations in XK still allowed complex formation with VPS13A, albeit at reduced levels compared with WT XK, and retained the ability to support ATP-induced PtdSer exposure. However, the XK double-mutant (DM) (E119A/E121A) failed to form a complex with VPS13A and could not mediate phospholipid scrambling (Figure 2, F and G). The pronounced effect of an arginine to Ala substitution may be explained by previous observations that Ala replacement of an interfacial arginine can have a greater impact on complex stability than the mutation of its acidic binding partner (35).

Taken together, these results demonstrate that the interactions between the XK β-hairpin residues E119 and E121 and the VPS13A C-terminal residues R3121 and R3119 are essential for the assembly of the mouse VPS13A–XK complex and scramblase activity at the plasma membrane.

### Ability of mouse XKR2 to respond to ATP for PtdSer exposure.

**Figure 3:**
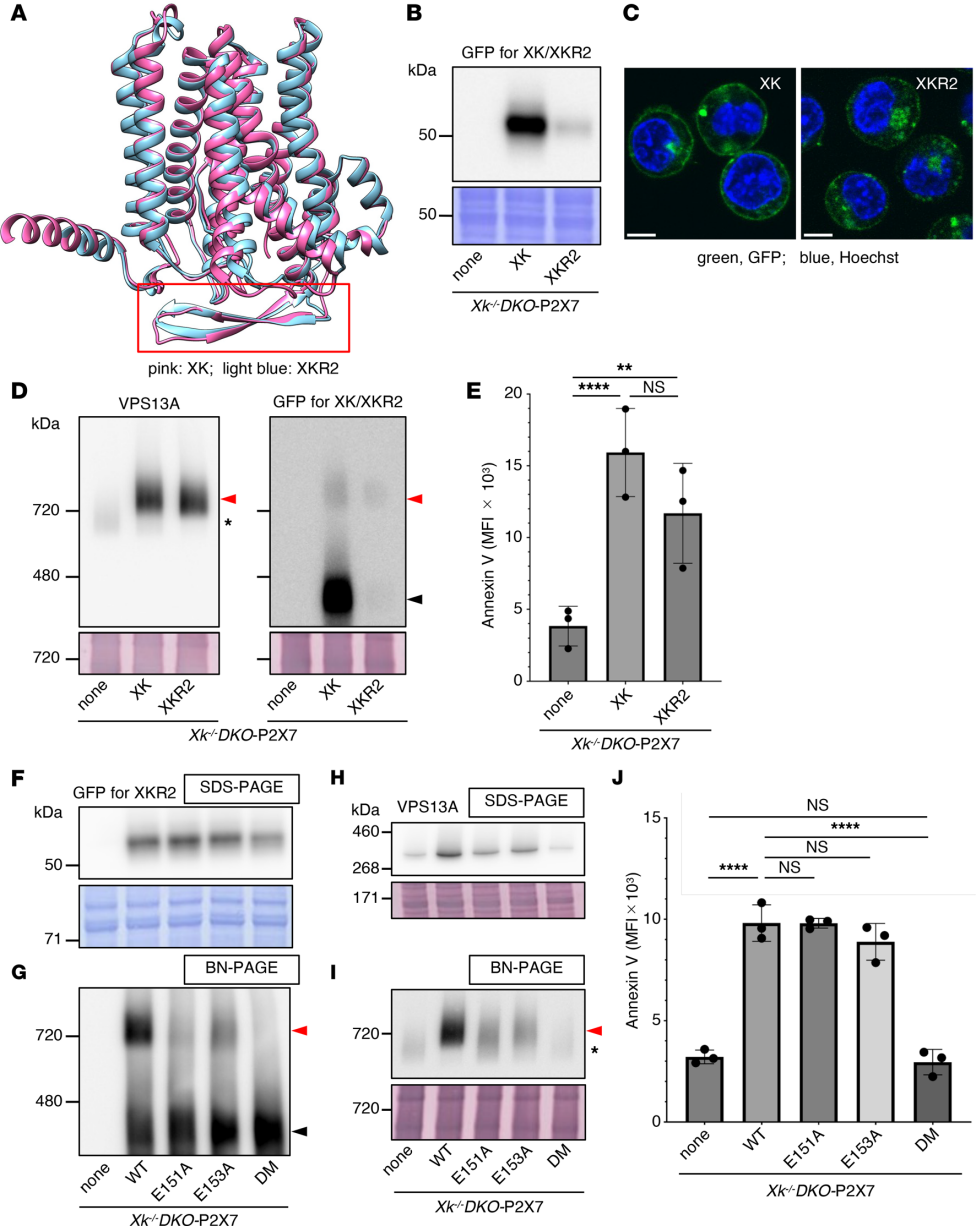
Interaction of mouse XKR2 with VPS13A and its ability to support ATP-induced PtdSer exposure. (**A**) Structural comparison of XK and XKR2. AlphaFold 3–predicted structures of mouse XK (AlphafoldDB, Q9QXY7) and XKR2 (AlphafoldDB, Q5GH68), excluding their N- and C-terminal regions, are shown. The β-hairpin region is enclosed in a red box. (**B–E**) Functional analysis of XKR2. *Xk^–/–^DKO*-P2X7 cells were transfected with mEGFP-tagged mouse XK or XKR2. (**B**) Whole-cell lysates were separated by SDS-PAGE and immunoblotted with an anti-GFP Ab or stained with CBB. (**C**) GFP fluorescence was observed using a microscope. Scale bar: 5 �m. (**D**) Membrane fractions were separated by BN-PAGE and immunoblotted with anti-VPS13A or anti-GFP Abs or stained with colloidal gold protein stain. Red arrowhead indicates VPS13A-XK-mEGFP or XKR2-mEGFP complex; band marked with an asterisk is an unidentified protein; black arrowhead indicates uncomplexed XK or XKR2. (**E**) Cells were treated with ATP and analyzed by flow cytometry for PtdSer exposure. (**F–J**) Effect of XKR2 missense mutations on VPS13A binding and scramblase activity. *Xk^–/–^DKO*-P2X7 cells were transformed with WT, E151A, E153A, or E151A/E153A DM mouse XKR2-mEGFP. (**F**) Whole-cell lysates were separated by SDS-PAGE and immunoblotted with anti-GFP Ab. (**G–I**) Membrane fractions were separated by BN-PAGE (**G** and **I**) and SDS-PAGE (**H**) and immunoblotted with anti-GFP Ab (**G**) or anti-VPS13A Ab (**H** and **I**). Red arrowheads indicate the VPS13A-XKR2 complex; the black arrowhead indicates uncomplexed XKR2. The band marked by an asterisk is an unidentified protein. (**J**) Cells were stimulated with ATP, and PtdSer exposure was analyzed by flow cytometry using Cy5–annexin V. In (**E** and **J**), PtdSer exposure is expressed as MFI. Data are presented as the mean ± SD (bar) of 3 independent experiments. Statistical significance was determined using Šídák’s multiple comparison test. **P < 0.01, ****P < 0.0001.

XKR2, encoded by *Xkrx*, is an orphan member of the XKR family with an unknown function (11, 36). Mouse XKR2 consists of 449 amino acids and shares 38.5% sequence identity with the 446–amino acid mouse XK protein, including the conservation of the β-hairpin structure (Supplemental Figure 4). The predicted tertiary structure of mouse XKR2 was highly similar to that of mouse XK (Figure 3A). In contrast with the *Xk* gene, the *Xkrx* gene is hardly expressed in WR19L cells (Gene Expression Omnibus accession GSE181171).

When expression vectors encoding mEGFP-tagged mouse XK or XKR2 were introduced into *Xk-*null *DKO-*P2X7 cells, the amount of expressed XKR2 protein was substantially lower than that of XK (Figure 3B). Both mouse XK and XKR2 were localized to the plasma membrane (Figure 3C). Remarkably, even this limited amount of mouse XKR2 recruited VPS13A to the membrane fraction, as revealed by BN-PAGE (Figure 3D). Most exogenously expressed mouse XK proteins were not associated with VPS13A; however, approximately 50% of mouse XKR2 formed a complex with VPS13A (Figure 3D). Consistent with this, mouse XKR2 supported ATP-stimulated PtdSer exposure (Figure 3E).

The 2 glutamic acid residues in XK (E119 and E121) required for interaction with VPS13A are conserved in XKR2 at the corresponding positions (E151 and E153) (Supplemental Figure 4). To examine whether XKR2 forms a complex with VPS13A in a manner similar to XK, the residues in C-terminally mEGFP-tagged XKR2 were mutated to Ala, either individually or in combination, and the mutants were expressed in *Xk^–/–^DKO*-P2X7 cells (Figure 3F). BN-PAGE and SDS-PAGE analyses of membrane fractions showed that both the recruitment of VPS13A to the membrane and the formation of the VPS13A-XKR2 complex were severely impaired in the DM (Figure 3, G–I). Consistently, the DM XKR2 failed to induce ATP-induced PtdSer exposure (Figure 3J). These results indicate that XKR2 forms a functional complex with VPS13A through a mechanism similar to that of XK and acts as a phospholipid scramblase.

### Missense mutations in VPS13A.

Nine missense mutations in human VPS13A (L67P, I90K, A1095P, S1452P, W2460R, Y2721C, I2771R, M3088R, and R3092H) have been reported in patients with *VPS13A* disease (33, 37–41). In addition, a VPS13A missense mutation (Q2697H) has been registered in the Human Gene Mutation Database (https://www.hgmd.cf.ac.uk) among 140 missense variants identified in patients with schizophrenia (42).

Mouse VPS13A shares 84% identity in amino acid sequence with human VPS13A, and human VPS13A compensates the null-mutation of mouse *Vps13a* gene in mouse cells (Supplemental Figure 1) indicating that VPS13A has no species-specificity between human and mouse, at least in ATP-induced scramblase activity. To assess the functionality of the VPS13A variants found in human patients, 10 corresponding mutations (*n* = 9 from patients with *VPS13A* disease and 1 from schizophrenia) were introduced into the mouse *Vps13a* cDNA using PCR-based site-directed mutagenesis (Supplemental Figure 5). The mutant cDNAs were expressed under the *EF-1α* promoter (43) and introduced into *Vps13a^–/–^DKO-*P2X7 cells via electroporation. Stable transformants were isolated by limiting dilution, and more than 10 individual clones per mutant were screened by Western blotting for mouse VPS13A expression. The highest-expressing clones of each mutant were selected for analysis, revealing 4 types of mutations (Table 1).

**Figure 4:**
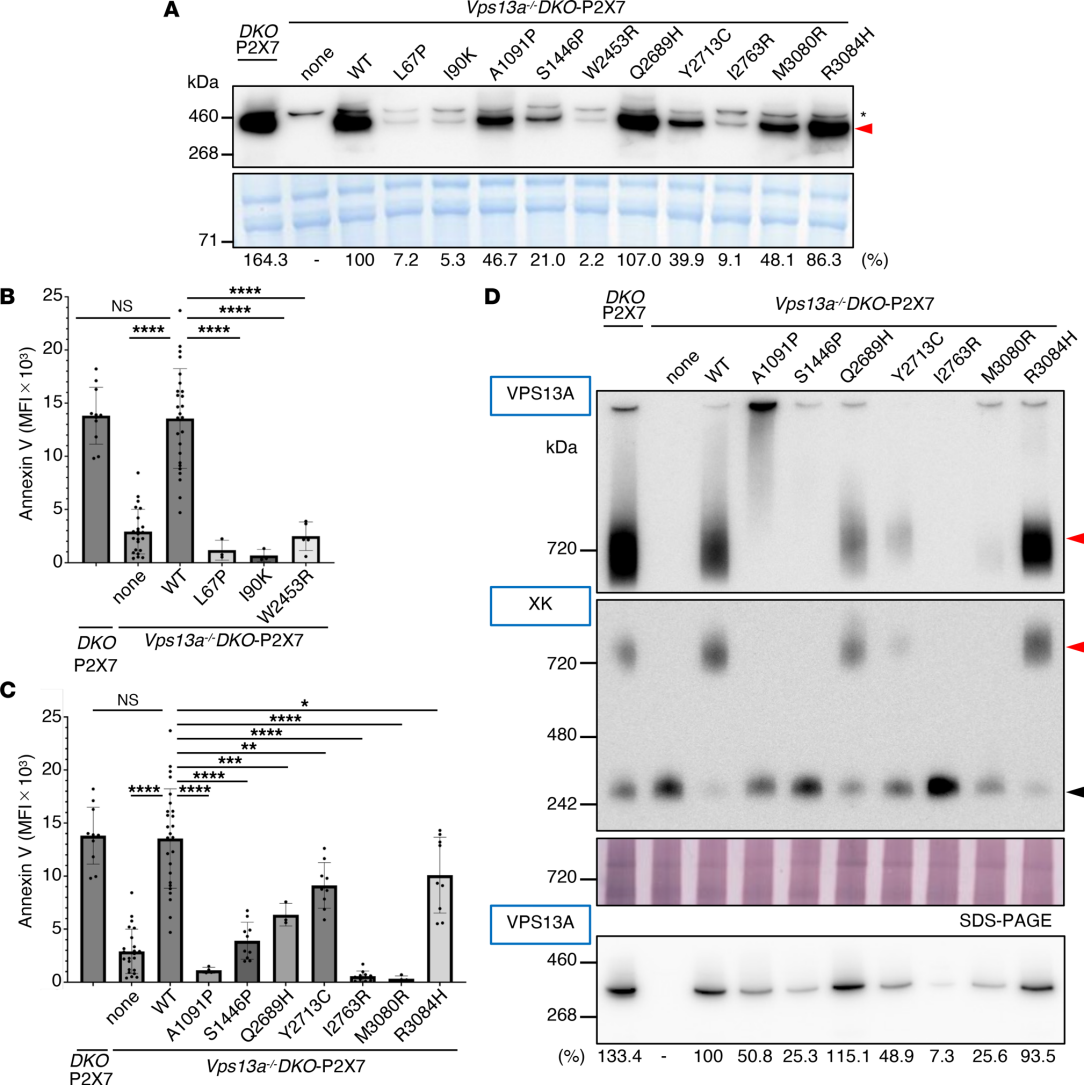
Characterization of mouse VPS13A missense mutations. (**A**) Stable expression of VPS13A missense mutations. *Vps13a^–/–^DKO-*P2X7 cells were stably transformed with WT or the indicated mouse VPS13A mutants. Whole-cell lysates from *DKO*-P2X7, *Vps13a^–/–^DKO*-P2X7 (none), and mutant transformants were analyzed by SDS-PAGE and Western blotting using anti-VPS13A Ab. The CBB-stained membrane is shown. Red arrowhead indicates VPS13A; * indicates a nonspecific band. (**B** and **C**) ATP-induced PtdSer exposure. Transformants expressing WT or mutant mouse VPS13A were treated with ATP, stained with Cy5–annexin V, analyzed by flow cytometry, and expressed as MFI. Data are presented as mean ± SD (bar) of more than 3 experiments. Statistical significance was determined using Dunnett’s multiple comparison test. *P < 0.05, **P < 0.01, ***P < 0.001, ****P < 0.0001. (**D**) Complex formation with XK. Membrane fractions were analyzed by BN-PAGE (upper panel) and SDS-PAGE (bottom panel) and Western blotting with anti-VPS13A or anti-XK Abs. Red arrowheads indicate the mouse VPS13A–XK complex; the black arrowhead indicates free XK. Membranes were stained with CBB (**A**) or colloidal gold protein stain (**D**). In (**A** and **D**), the band intensity detected by Western blotting was quantified by densitometry and expressed relative to that detected in cells expressing WT-VPS13A.

In the first class of mutants, 3 variants (L67P, I90K, and W2453R) were expressed at barely detectable levels (Figure 4A), and their corresponding transformants failed to exhibit ATP-induced PtdSer exposure (Figure 4B), indicating that these mutations destabilized VPS13A or severely impaired its expression. The second class of mutants (S1446P, Q2689H, Y2713C, and R3084H) showed only modest effects on expression and/or function (Figure 4, A, C, and D). Q2689H and R3084H were expressed at near-WT levels and supported ATP-induced PtdSer exposure via complex formation with XK, although Q2689H showed reduced complex formation. Y2713C was expressed at approximately 40% of the WT levels, yet it retained ATP-induced PtdSer exposure and XK complex formation.

In contrast, A1091P and M3080R mutants were expressed at higher levels than Y2713C (Figure 4A) but failed to support ATP-induced PtdSer exposure (Figure 4C). VPS13A recruitment to the plasma membrane is dependent on XK (22). SDS-PAGE of membrane fractions showed that the A1091P protein was present at approximately 50% of the WT VPS13A level, comparable to that of Y2713C (Figure 4D). However, BN-PAGE revealed that the A1091P-XK complex was markedly larger than the WT VPS13A-XK or Y2713C VPS13A-XK complexes (Figure 4D), suggesting that A1091P induced aberrant aggregation. Conversely, the M3080R mutant was recruited to the membranes less efficiently (25.6% of WT VPS13A) and showed little or no detectable interaction with XK (Figure 4D).

Taken together, these findings suggest the disease phenotypes observed in patients carrying mutations corresponding to the mouse VPS13A A1091P or M3080R variants can be attributed to the impaired phospholipid scrambling activity of the VPS13A–XK complex. The A1091P mutant retains the ability to bind XK at the plasma membrane but forms nonfunctional aggregates, whereas the M3080R mutant interacts inefficiently with XK, resulting in little or no functional complex formation.

### Giant-cell morphology of I2763R-expressing cells.

**Figure 5:**
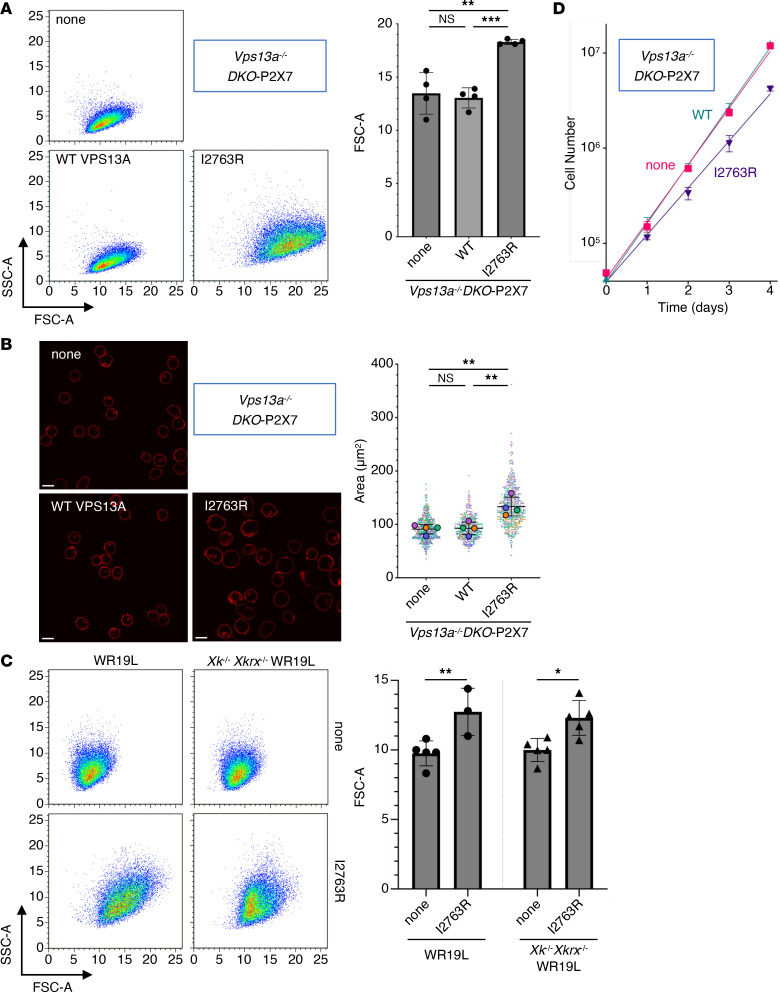
Characterization of the mouse VPS13A I2763R mutant. (**A**) Flow cytometry analysis. *Vps13a^–/–^DKO*-P2X7 and transformants expressing WT or I2763R mouse VPS13A were analyzed by flow cytometry. FSC-A/SSC-A profiles of the propidium iodide–negative population are shown. The experiments were performed at least 3 times, and the FSC values are presented as the mean ± SD (bar). (**B**) Microscopy-based size measurements. The cells were stained with PlasMem and observed using a confocal microscope. Cell size was quantified using Cellpose-SAM and Fiji; the distributions (in �m^2^) are plotted by SuperPlots (78). Small dots represent individual cells; large dots represent the mean of 4 independent experiments. Scale bar 10 �m. (**C**) Effect of I2763R in the XK-intact *Xk* or *Xkrx*-null genetic background. I2763R mouse VPS13A was expressed in WR19L or *Xk^–/–^Xkrx^–/–^*WR19L cells, and stable transformants were analyzed for FSC-A/SSC-A profiles using flow cytometry. Mean ± SD of at least 3 experiments is shown. (**D**) Growth assay. Cells were seeded at 4 × 10^4^ cells/mL, cultured for 4 days, and their growth was monitored. In (**A** and **B**), statistical significance was determined using 1-way ANOVA with Tukey’s multiple comparison test, and by using 2-way ANOVA with Šídák’s multiple comparison test in (**C**). *P < 0.05; **P < 0.01, ***P < 0.001.

Western blot analysis showed that the expression level of the mouse VPS13A I2763R mutant protein was approximately 9.1% of that of WT VPS13A (Figure 4A). Consistent with this low expression level, cells expressing I2763R did not respond to extracellular ATP with PtdSer exposure (Figure 4C), and no complex formation with mouse XK was detected by BN-PAGE (Figure 4D). In contrast, flow cytometry revealed that the forward scatter area/side scatter area (FSC-A/SSC-A) profiles of I2763R-expressing cells were markedly different from those of *Vps13a^–/–^DKO-*P2X7 cells or their transformants expressing WT mouse VPS13A (Figure 5A). Specifically, both FSC-A and SSC-A were increased in I2763R-expressing cells, indicating an increase in cell size and granularity. This phenotype was confirmed by microscopic observation of PlasMem-stained cells, and quantitative image analysis demonstrated that I2763R-expressing cells were approximately 30% larger than *Vps13a^–/–^DKO-*P2X7 cells or *Vps13a^–/–^DKO-*P2X7 cells expressing WT mouse VPS13A (Figure 5B).

Strikingly, giant-cell morphology was also observed when the mouse VPS13A I2763R mutant was expressed in WT WR19L cells containing endogenous VPS13A (Figure 5C), indicating a dominant phenotype of the mutant. Furthermore, the expression of I2763R in *Xk^–/–^Xkrx^–/–^* cells still caused high FSC-A/SSC-A values, suggesting that this phenotype is independent of XK/XKR2. Because cell size is linked to the growth rate and cell cycle progression (44, 45), we compared the growth kinetics. Mouse VPS13A I2763R mutant–expressing cells grew markedly slower (doubling time [*T_d_*] = 14.63 hours) than either parental *Vps13a*-null *DKO* (*T_d_* = 12.10 hours) or WT mouse VPS13A-reconstituted cells (*T_d_* = 11.73 hours) (Figure 5D).

### Damaged and fragmented ER in the I2763R mutant.

**Figure 6:**
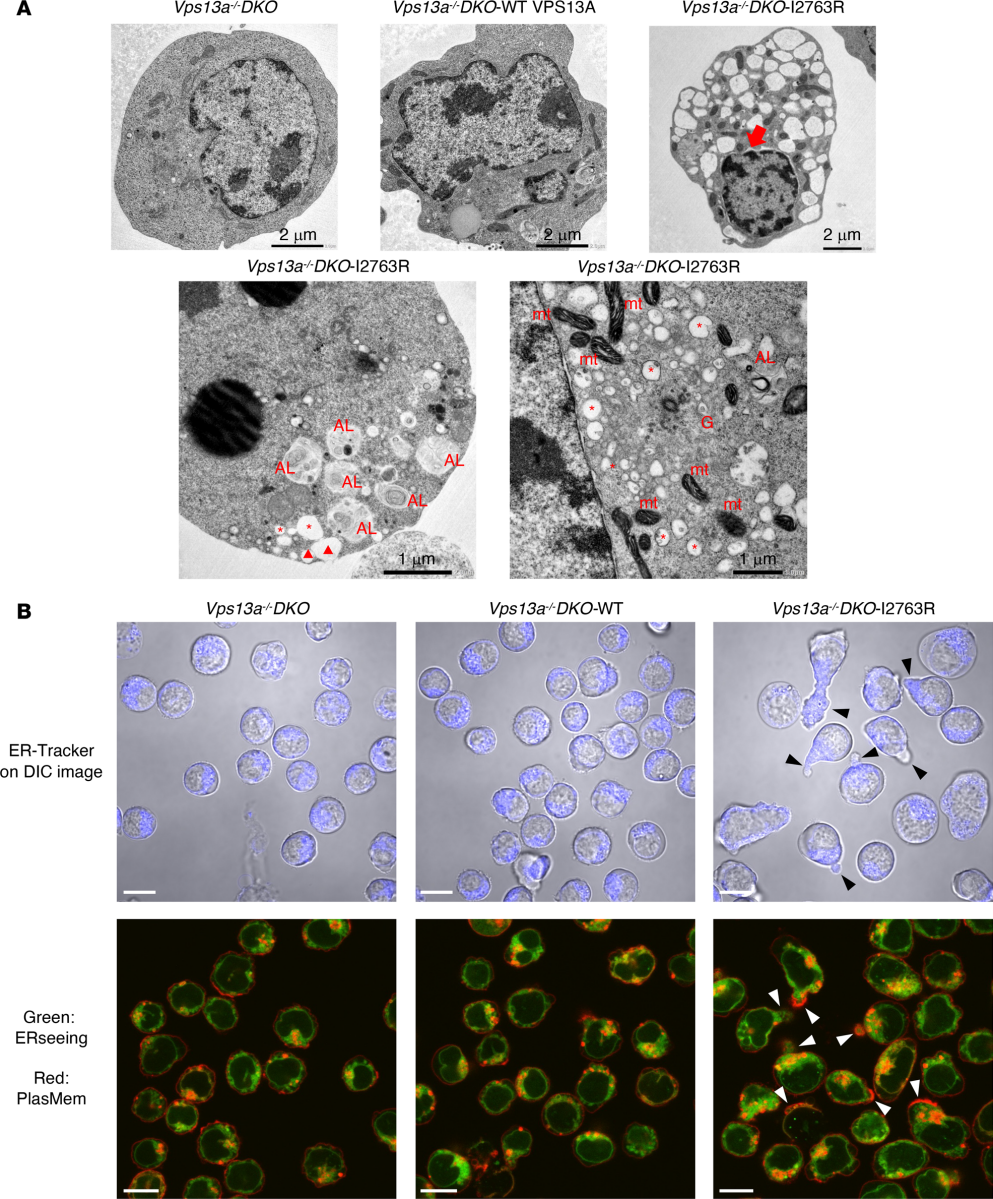
Damaged ER in cells expressing the I2763R mouse VPS13A mutant. (**A**) Transmission electron microscopy. mt, mitochondria; G, Golgi apparatus; AL, autophagosome/lysosome; *, fragmented or damaged ER; red triangles, damaged ER fusing with plasma membrane. The swollen nuclear membrane is indicated by a red arrow. (**B**) Confocal microscopy images. Cells were stained with ER-Tracker (top panels) or ERseeing and PlasMem (bottom panels). The regions where ERs are polarized to the plasma membrane are indicated by arrowheads. DIC, differential interference contrast. Scale bar 10 �m.

VPS13A is a lipid transfer protein that mediates lipid transfer between intracellular organelles and plasma membranes (1, 2). To determine which organelles were affected by the I2763R mutation, we analyzed the cells, using transmission electron microscopy. As shown in Figure 6A, *Vps13a*-deficient cells displayed normal morphology, and re-expression of WT mouse VPS13A had little effect, indicating that VPS13A is dispensable for maintaining plasma membrane and organelle integrity in *Vps13a^–/–^DKO-*P2X7 cells. In contrast, cells expressing the I2763R mouse VPS13A mutant contained numerous vacuole-like structures, consistent with the increased SSC-A in the FACS profile (Figure 5A). Closer examination revealed that these vacuoles contained fragmented ER and, in some cases, they fused with the plasma membrane. Swelling of the nuclear envelope was occasionally observed. Although the mitochondria were largely intact, some showed signs of damage, whereas the Golgi apparatus appeared unaffected.

Fluorescence staining with ER-Tracker and ERseeing further confirmed these findings. In *Vps13a^–/–^DKO-*P2X7 cells and those expressing WT mouse VPS13A, the ER was evenly distributed throughout the cytoplasm. In contrast, the I2763R mutant frequently caused polarized ER localization in plasma membrane protrusion (Figure 6B). These regions often show strong PlasMem staining, suggesting localized lipid enrichment (46). Together, these results demonstrate that the I2763R mutant disrupts ER integrity, leading to fragmentation and abnormal ER–plasma membrane interactions, possibly reflecting localized lipid transfer from the ER to the plasma membrane.

## Discussion

In *Saccharomyces cerevisiae*, the single *VPS13* gene mediates the bulk lipid transfer required for the expansion and maintenance of organelle membranes (47, 48). In contrast, mammals possess 4 *VPS13* paralogs, and mutations in these genes are associated with distinct human disorders (37, 49–52). Specifically, mutations in *VPS13A* cause *VPS13A* disease, *VPS13B* mutations cause Cohen syndrome, *VPS13C* mutations have been linked to familial parkinsonism with Lewy bodies (50, 53), and *VPS13D* is associated with spastic ataxia or spastic paraplegia (54). Despite these genetic associations, the biochemical mechanisms underlying *VPS13A* disease remain poorly defined, largely because of the lack of robust functional assays for VPS13A in mammalian cells.

We previously demonstrated, using mouse WR19L lymphoma cells, that VPS13A is recruited to the plasma membrane via XK and mediates ATP-stimulated PtdSer exposure (22). Using this assay system, we examined the functional consequences of a series of VPS13A deletions and missense mutations. Analysis of C-terminal truncations and targeted mutagenesis of mouse VPS13A and XK revealed that the β-hairpin of XK interacts with a protruding β-α-β structural motif within the C-terminal PH domain of VPS13A. Xu et al. (55) recently reported that *Vps13a^–/–^Vps13c^–/–^* double-knockout mice, but not single-knockout mice, are embryonic lethal due to defective erythropoiesis, indicating functional redundancy between VPS13A and VPS13C. In agreement with this notion, the C-terminal region of mouse VPS13C also contains a β-α-β fold similar to that of VPS13A (Supplemental Figure 6). Therefore, it is of interest to determine whether VPS13C interacts with XK or XKR2 to support ATP-induced phospholipid scrambling.

In *S*. *cerevisiae*, *vps*13-null mutants display pleiotropic phenotypes, including defective sporulation (56–58), loss of mitochondrial DNA (29), and impaired vacuolar protein sorting (59). Similarly, *Drosophila* carries a single *Vps13* gene, and its disruption leads to reduced lifespan and age-dependent neurodegeneration (60). Importantly, these phenotypes can be partially rescued by the expression of human VPS13A (29, 56, 57, 60), indicating that VPS13 proteins in yeast, flies, and mammals share conserved molecular functions. Notably, both yeast and *Drosophila* VPS13 proteins possess a C-terminal β-α-β motif (Supplemental Figure 6), even though XK homologs are absent in these organisms. Whether VPS13-interacting proteins in yeast or *Drosophila* — potentially identifiable via the Molecular Interaction Search Tool — contain β-hairpin motifs analogous to those of XK remains an open question.

Among the 10 mouse VPS13A missense mutations analyzed in this study, 6 (L67P, I90K, A1091P, W2453R, I2763R, and M3080R) caused severe defects in protein expression and/or ATP-triggered PtdSer exposure (Tables 1 and 2). These results closely mirror the reported clinical and biochemical phenotypes of patients harboring the corresponding human mutations (L67P, I90K, A1095P, W2460R, I2771R, and M3088R, respectively) (33, 37, 40, 41, 61, 62).

In contrast, 4 mouse VPS13A mutants (S1446P, Q2689H, Y2713C, and R3084H) were expressed at appreciable levels and retained ATP-stimulated PtdSer exposure, indicating only modest effects on protein stability or on scrambling activity. The mouse Y2713C and Q2689H mutations correspond to the human VPS13A Y2721C and Q2697H variants. The human Y2721C variant was initially reported as a heterozygous mutation in a patient diagnosed with chorea-acanthocytosis (37, 63). However, a recent reanalysis by Danek et al. (62) demonstrated that compound heterozygous mutations in the *PANK2* (pantothenate kinase 2) gene account for the patient’s phenotype and that VPS13A Y2721C probably represents a benign polymorphism.

Notably, 3 of these residues — including Q2689, which was identified as a heterozygous variant in a patient with schizophrenia (42) — are not conserved in VPS13C, whereas all 6 residues associated with severe functional impairment are highly conserved (Supplemental Figure 7), underscoring their importance for VPS13A’s stability and function. Although a discrepancy was observed between the expression levels of the human R3092H variant and the corresponding mouse R3084H mutant (Table 2), these variants are likely polymorphic or may not contribute to disease severity.

**Figure 7:**
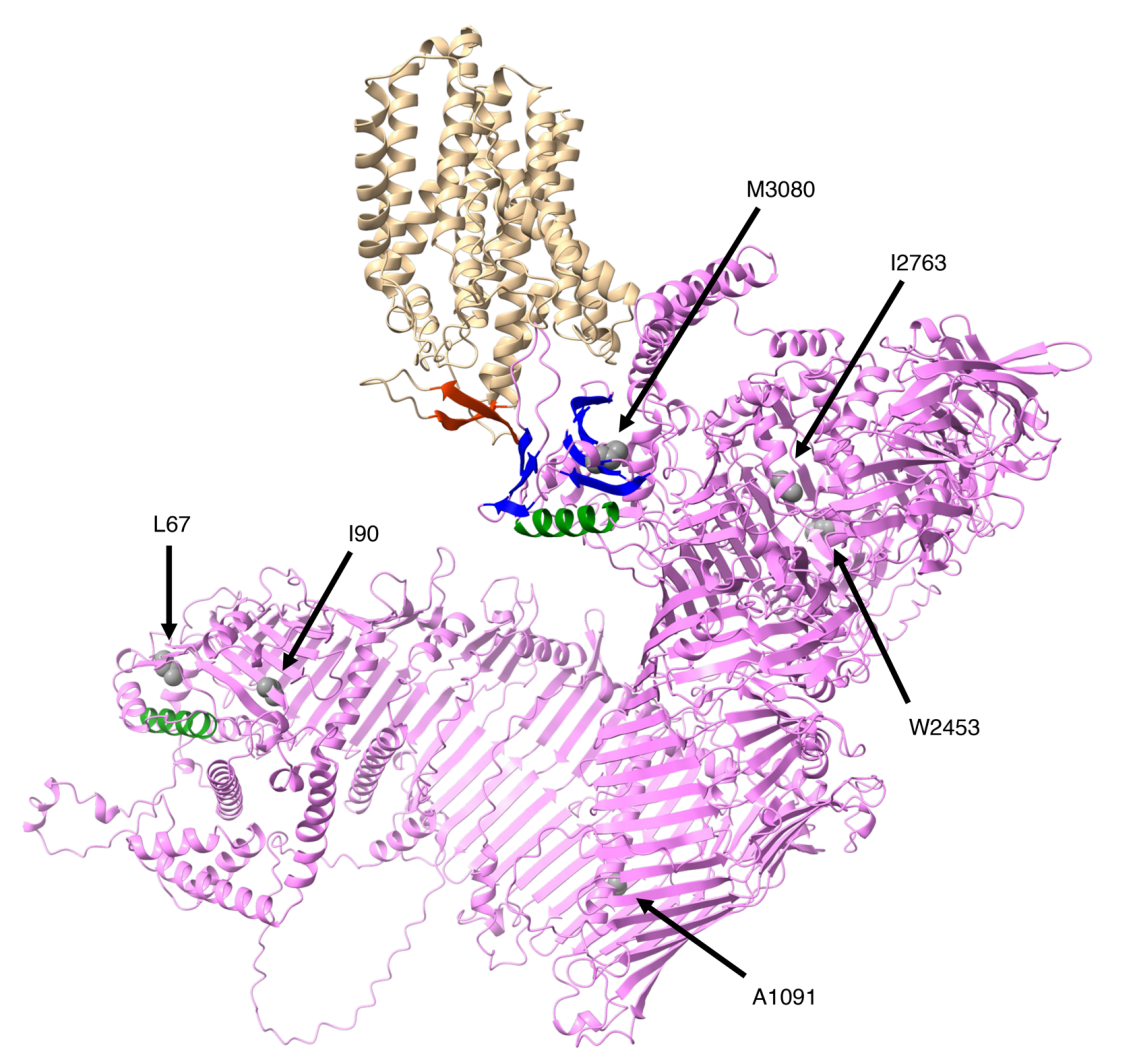
Pathological missense mutations in the mouse VPS13A. The AlphaFold 3–predicted tertiary structure of the mouse VPS13A-XK complex is shown with 6 residues, the missense mutations of which strongly affect protein expression or function. XK is shown in tan with a hairpin structure in orange. VPS13A is colored violet, and the β-strands and α-helix in the N- and C-termini are blue and green, respectively. The essential 6 residues are shown as sphere structures with colored elements.

Structural analysis provides insights into the molecular basis of these defects. L67, I90, and W2453 are located within the β-strands of mouse VPS13A (Figure 7). The L67P substitution introduces a proline residue that is predicted to disrupt β-strand integrity, whereas the I90K and W2453R substitutions introduce basic residues into hydrophobic cores surrounding I90 (L48, F55, L88, I160, and Y162) and W2453 (V2462, I2472, L2481, and F2494), respectively (Supplemental Figure 8). Therefore, these changes are expected to destabilize the folding by disrupting critical hydrophobic interactions. Among the remaining nonfunctional variants, M3080R likely perturbs the β-α-β motif required for XK binding (Figure 7 and Supplemental Figure 2), whereas A1091P, located in the central region of the protein, may obstruct the phospholipid passage through the VPS13A channel. Collectively, these observations are consistent with the notion that *VPS13A* disease can arise from mutations that compromise VPS13A-mediated ATP-dependent PtdSer exposure.

**Figure 8:**
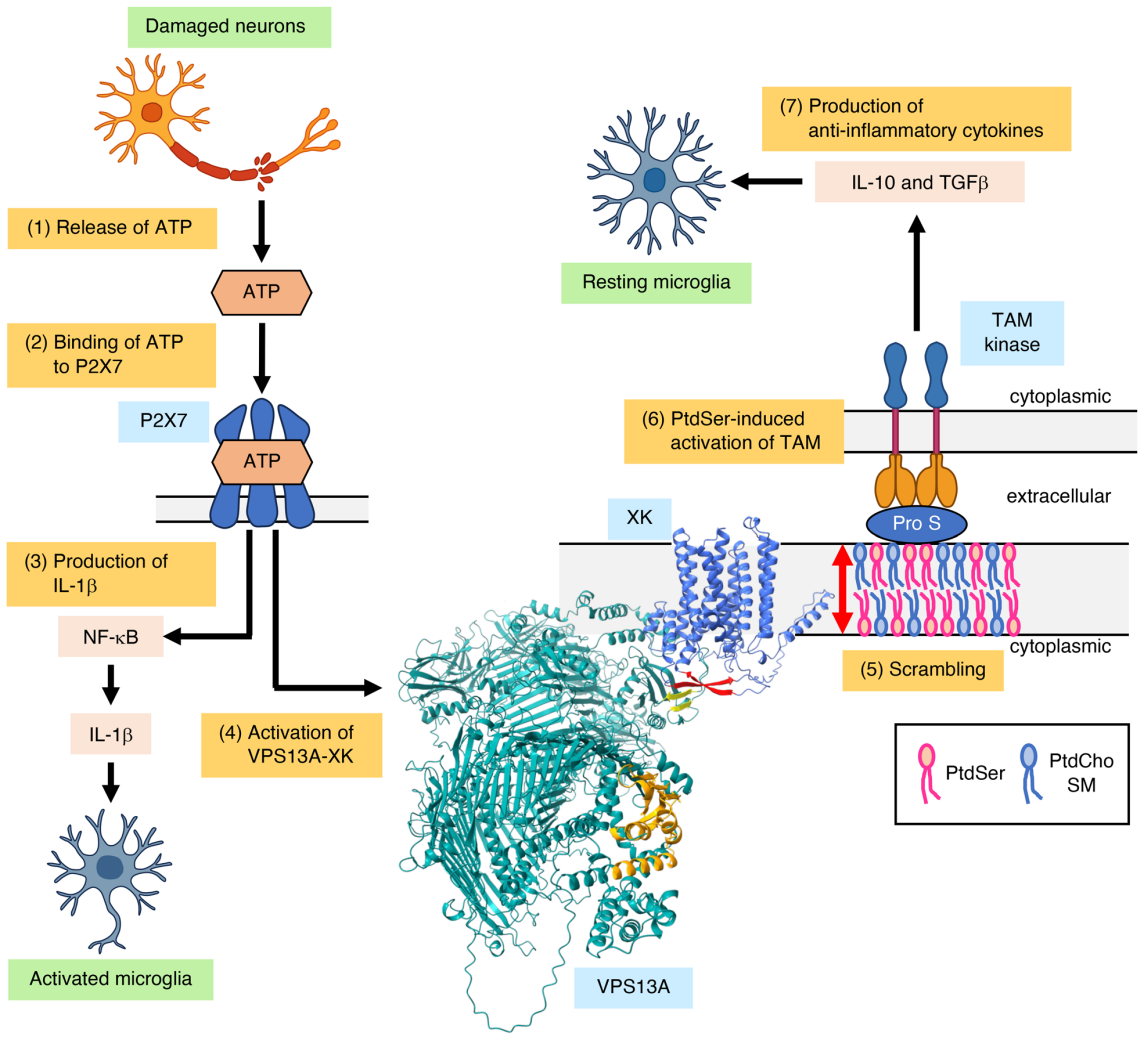
A model of the anti-inflammatory effect of PtdSer exposed by the VPS13A-XK complex. (1) ATP is released from damaged neurons and necrotic cells. (2) ATP binds to the P2X7 receptor on microglia. (3) ATP-activated P2X7 triggers NF-κB signaling, leading to IL-1β production and inflammation. (4) In parallel, ATP-activated P2X7 stimulates the VPS13A–XK complex through an unidentified mechanism. (5) The activated VPS13A-XK complex functions as a scramblase, exposing PtdSer to the cell surface. (6) The exposed PtdSer binds to Protein S, which then engages TAM tyrosine kinase receptors on microglia. (7) Activated TAM kinases induce anti-inflammatory cytokines, including IL-10 and TGF-β, thereby suppressing the inflammation. In *VPS13A* disease, PtdSer-mediated anti-inflammatory signaling is impaired, leading to chronic inflammation and progressive nervous system damage.

Based on these findings, we propose the following model (Figure 8). ATP released from neuronal tissues, particularly under injury conditions, activates P2X7 receptors that are highly expressed in microglia and oligodendrocytes (64). P2X7 activation promotes inflammatory signaling via the NF-κB pathway (20) and simultaneously induces PtdSer exposure through activation of the VPS13A–XK complex (22). Because cell-surface PtdSer exerts anti-inflammatory effects (65), VPS13A system dysfunction may impair this regulatory mechanism, leading to chronic neuroinflammation and progressive neuronal loss (61). This model is consistent with the neuroinflammatory features observed in mouse model of *VPS13A* disease (66, 67).

Finally, the I2771R mutation was identified as a heterozygous variant in a patient with *VPS13A* disease, although detailed clinical characterization was not reported (38). Correspondingly, the mouse VPS13A I2763R mutant exhibited markedly reduced expression and loss of ATP-stimulated PtdSer exposure, yet exerted a dominant gain-of function effect on cell size. Notably, in *S*. *cerevisiae*, the VPS13 D716H and L1627S mutations act as dominant gain-of-function alleles that bypass the ER–mitochondrial contact system (68). By analogy, the VPS13A I2763R mutation may bypass the ER–plasma membrane contact system, resulting in excessive lipid transfer and plasma membrane expansion, reminiscent of osmotically induced membrane growth (69). The presence of damaged nuclear and mitochondrial membranes in I2763R-expressing cells is consistent with VPS13A’s proposed role in lipid transfer between the ER and multiple organelles (1). It is important to determine whether the corresponding yeast mutation (I2749R) (57) exhibits similar phenotypes. Moreover, because the yeast Vps13 D716 and L1627 residues are well conserved in human and mouse VPS13A, it would be of interest to test whether equivalent mouse mutations exert dominant effects on lipid transfer between the ER and mitochondria.

## Methods

### Cell lines, Abs, and reagents.

WR19L is a mouse T cell lymphoma cell line (ATCC, TIB-52). *Tmem16f^–/–^P2rx7^–/–^*WR19L (*DKO*) and *Vps13a^–/–^DKO*, as well as their transformants expressing mouse P2X7 and/or VPS13A (*DKO*-P2X7, *Vps13a^–/–^DKO*-P2X7, and *Vps13a^–/–^DKO*-P2X7-VPS13A), were described previously (22). WR19L and its derivatives were grown in RPMI 1640 medium supplemented with 10% FBS. *DKO*-P2X7 and *Vps13a^–/–^DKO*-P2X7 cells were grown in the presence of 800 �g/mL G418, whereas *Vps13a^–/–^DKO*-P2X7-VPS13A and all other *Vps13a^–/–^DKO* transformants expressing VPS13A variants were grown in the presence of 800 �g/mL G418 and 1 �g/mL puromycin. HEK293T cells (ATCC, CRL-3216) were cultured in DMEM supplemented with 10% FBS.

Rabbit anti-XK (catalog HPA019036) and anti-VPS13A (catalog HPA021662) Abs were purchased from Atlas Antibodies. HRP–rabbit anti-GFP was obtained from MBL (anti-GFP pAb-HRP-DirecT; 598-7), and HRP–goat anti-rabbit Igs (catalog P0448) were from Agilent. Alexa 647-(catalog MCA4713A647) or Alexa 488-(catalog MCA4713A488) conjugated rat anti-mouse P2X7 mAbs (clone Hano43) were purchased from Bio-Rad Laboratories. Cy5-annexin V was from BD Biosciences (AB_2869267).

Hoechst 33342 (catalog H3570), ER-Tracker Blue-White DPX (catalog HY-D1429), and SYTOX Blue (catalog S34857) were obtained from ThermoFisher Scientific. PlasMem Bright Red (46) was purchased from Dojindo (346-09771), ERseeing (endoplasmic reticulum green) from Funakoshi (FDV-0038) (70), and colloidal gold protein stain from Bio-Rad Laboratories (1706527). (*p-*Amidinophenyl)methanesulfonyl fluoride hydrochloride (*p*-APMSF) was obtained from Wako (019-26331). Pefabloc SC (catalog 1142986800) and Complete EDTA-free protease inhibitor cocktail (catalog 11873580001) were purchased from Roche. Tris(2-carboxyethyl)phosphine hydrochloride (TCEP-HCl) was obtained from Nacalai Tesque (07277). *n*-Dodecyl-β-d-maltoside (DDM) was obtained from Dojindo (341-06161) and cholesteryl hemisuccinate (CHS) was from Sigma-Aldrich (C6512). FuGENE 6 was purchased from Promega (E2691).

### Plasmids.

pGag-pol-IRES-bsr (71) was a gift from T. Kitamura (Institute of Medical Science, the University of Tokyo, Tokyo, Japan). pCMV-VSV-G and pAdVAntage were obtained from RIKEN (RDB04392) and Promega (U47294), respectively. The human (h)VPS13A^mCherry plasmid (48) was obtained from Addgene (118758), and its coding sequence was transferred to pPEF-BOS-EX (RIKEN, RDB18971) to generate pPEF-hVPS13A^mCherry. The expression vectors for mouse P2X7 (72) and pPEF-VPS13A (22) were described previously. The GFP portion of pMXs-puro-XK-GFP and pMXs-puro-XKR2-GFP (36) was replaced with mEGFP (73). Mutant VPS13A, XK, and XKR2 were constructed using pPEF-VPS13A (Supplemental Figure 5), pMXs-puro-XK-mEGFP, and pMXs-puro-XKR2-mEGFP as templates. All constructs were verified by DNA sequencing. The forward and reverse primers used for PCR are listed in Supplemental Tables 2–4 and were synthesized by FASMAC Co.

To delete mCherry from pPEF-hVPS13A^mCherry, 2 DNA fragments were prepared by PCR — 1 with a forward primer containing BlpI and a reverse joint primer, and the other with a forward joint primer and a reverse primer containing SwaI (Supplemental Table 1). The PCR products were purified using the ExoSAP-IT Express PCR Product Cleanup Kit (ThermoFisher Scientific, 75001) and inserted between the BlpI-SwaI sites of pPEF-hVPS13A^mCherry using the In-Fusion HD Cloning Kit (TaKaRa, 639650) to generate pPEF-hVPS13A. The resulting plasmids were introduced into *E*. *coli* DH5α or XL10-Gold cells (Agilent Technologies, 200314).

For the L67P and I90K mutants of mouse VPS13A, 2 DNA fragments were generated by PCR using Primer_F1 and L67P_R or I90K_R, and L67P_F or I90K_F with Primer_R1, respectively (Supplemental Table 2 and Supplemental Figure 5). The PCR products were inserted between the PacI and EcoRV sites of pPEF-VPS13A. The other mutants were prepared similarly with mutagenesis primers along with the corresponding forward and reverse primers. For R3127Δ, 2 DNA fragments were generated by PCR using Primer_F6 and R3127Δ_R, and R3127Δ_F and Primer_R6, whereas for E3136Δ, Primer_F6 and E3136Δ_R, and E3136Δ_F and Primer_R6 were used. The resulting fragments were inserted into NheI-digested pPEF-VPS13A vector.

For mouse XK mutants, 2 PCR fragments were prepared with Primer_F7 and reverse mutagenesis primer and forward mutagenesis primer and Primer_R7 (Supplemental Table 3) and inserted between BamHI and EcoRI of pMXs-puro-XK-mEGFP. Mouse XKR2 mutants were generated similarly using Primer_F8 and Primer_R8 (Supplemental Table 4) and inserted between the BamHI and EcoRI sites of pMXs-puro-XKR2-mEGFP.

### Cell transformation.

To introduce the VPS13A expression plasmid into WR19L-derived cell lines, 10–20 �g DNA was digested with PvuI and electroporated into 1×10^6^ cells using a NEPA21 Super Electroporator (Nepa Gene). After 48 hours, puromycin was added to the medium at a final concentration of 1.0 �g/mL, cultured for 2–10 days, and subjected to limiting dilution. At least 10 puromycin-resistant clones were screened for VPS13A expression by Western blotting, and 1 high-expressing clone was selected for further analyses.

Regarding the transformation of mouse P2X7, XK, XKR2, or their derivatives, HEK293T cells were transfected with pMXs-puro or pMXs-neo vectors carrying the respective cDNA along with pGag-pol-IRES-bsr, pCMV-VSV-G, and pAdVAntage using FuGENE 6, and cultured for 48 hours. Retroviruses in the supernatant were collected by centrifugation at 6,000*g* for 16 hours and used to infect WR19L derivatives. After 24 hours, puromycin or G418 was added to a final concentration of 1.0 �g/mL or 800 �g/mL, respectively, and P2X7- and/or GFP-positive transformants were sorted using FACSAriaII (BD Bioscience).

### Gene editing.

Mouse *Xk* and *Xkrx* genes were knocked out using the CRISPR/Cas9 system (74), as described previously (22). The target sequence for sgRNA for *Xk* (5′-CTTTCTCCACCTCCTCTGAA-3′) was from Ryoden et al. (22). The mouse sequence (5′-TGTAGTCATCATACTTGATC-3′) corresponding to that used to knock out human *XKRX* (25) was adapted for the *Xkrx* gene. Complementary oligonucleotides carrying the sgRNA sequence were annealed, ligated into pX459v2 (75), and introduced into WR19L cells twice by electroporation at 3–10 day intervals, subjected to limiting dilutions, and screened by sequencing the target regions of the *Xk* and/or *Xkrx* genes.

### Preparation of whole-cell lysates and membrane fractions.

To prepare whole-cell lysates, 1.5 ×10^6^ cells were incubated at 4°C for 1 hour in 0.1 mL of lysis buffer (20 mM Tris-HCl at pH 7.5, 50 mM KCl, 1 mM MgCl_2_, 10% glycerol, 1% DDM, 0.1% CHS, 1 mM *p*-APMSF, and a protease inhibitor mixture [Complete, EDTA-free; Roche]). After centrifugation at 20,000*g* and 4°C for 15 minutes, the supernatants were collected as whole-cell lysates. Protein concentration was determined using the Pierce BCA Protein Assay Kit (ThermoFisher Scientific, 23225).

For the membrane fractions, between 1.6 × 10^7^ and 2.0 × 10^7^ cells in 4 mL of 20 mM HEPES-NaOH buffer (pH 7.0) containing 150 mM NaCl, 1 mM EDTA, 1 mM EGTA, 0.4 mM Pefabloc SC, 1 mM *p*-APMSF, 1 mM TCEP, and a protease inhibitor mixture were disrupted at 4°C using an ultrasonic disrupter (QSONICA) (0.5-second sonication at an amplitude of 30 followed by 0.5-second cooling intervals for a total sonication period of 1 minute). After successive centrifugation at 800*g* at 4°C for 10 minutes and at 8,000*g* for 10 minutes, membrane fractions were collected by centrifugation at 100,000*g* for 1 hour, resuspended in 0.09 mL of lysis buffer, homogenized with 29G needle syringes for 10–25 strokes, and incubated at 4°C for 2 hours. Insoluble materials were removed as described above, and the supernatant was used as the membrane fraction.

### SDS-PAGE, BN-PAGE, and Western blotting.

For SDS-PAGE, whole-cell lysates (4.7 μg) or membrane fractions (1.1 μg) were incubated at room temperature for 15 minutes to 2 hours in SDS sample buffer (62.5 mM Tris-HCl [pH 6.8], 2% SDS, 10% glycerol, 2.5% 2-mercaptoethanol, and 0.005% bromophenol blue) and separated on either a 7.5% SDS-PAGE gel or a 5%–10% gradient gel. HiMark Pre-stained Protein Standard (ThermoFisher Scientific, LC5699) and Precision Plus Protein Standards, Dual Color (Bio-Rad Laboratories, 1610374) were used as molecular weight markers.

BN-PAGE was performed using a system provided by ThermoFisher Scientific. In brief, membrane fractions (1.1 μg) were mixed with 0.25 volume of NativePAGE Sample Buffer (catalog BN2003) and 1/20 volume of NativePAGE 5% G-250 Sample Additive (catalog BN2004) and loaded onto a NativePAGE 4%–16% Bis-Tris gel (catalog BN2111BX10 or BN2112BX10). Electrophoresis was performed at 150 V at 4°C for 35 minutes, after which the concentration of CBB G-250 in the running buffer was reduced from 0.02% to 0.002%. Electrophoresis was then performed sequentially at 150 V for 25 minutes, 250 V for 30 minutes, and 350 V for 20 minutes. NativeMark Unstained Protein Standard (ThermoFisher Scientific, LC0725) was used as the molecular weight marker.

For Western blotting, proteins were transferred from gels to PVDF membranes (Immobilon-P; Merck, IPVH00010) either immediately after SDS-PAGE or after BN-PAGE gels were incubated in SDS-PAGE running buffer (25 mM Tris-HCl [pH 8.3], 192 mM glycine, 0.1% SDS) at room temperature for 15 minutes. Membranes were blocked with 5% skim milk and probed with HRP-conjugated Abs. Signals were detected using the Immobilon Western Chemiluminescent HRP substrate (Merck, WBKLS0500). As a loading control, proteins on the membranes were visualized by staining with CBB R-250 or colloidal gold protein stain.

### Flow cytometry and ATP-induced PtdSer exposure.

ATP-induced exposure of PtdSer was assayed by annexin V binding, as described previously (72). Briefly, the cells were seeded at a density of 2 × 10^5^ cells/mL 1 day before the experiment. On the day of analysis, 4 × 10^5^ cells were washed with chilled PBS and pre-incubated for 10 minutes at 4°C in 400 �L of annexin buffer (10 mM HEPES-NaOH at pH 7.5, 140 mM NaCl, and 2.5 mM CaCl_2_) containing Cy5–annexin V (1:1,000 dilution) and either 2.5 �g/mL propidium iodide or 250 nM SYTOX Blue. The cells were then stimulated with 500 μM ATP at 4°C for 10 minutes. Annexin V–binding profiles were acquired by flow cytometry using a FACSCanto II (BD Biosciences) and analyzed with FlowJo software (version 7.6.5; BD Biosciences).

### Confocal microscopy and cell size estimation.

To localize XK and XKR2, 2 × 10^5^ cells expressing XK-mEGFP or XKR2-mEGFP were washed with PBS and suspended in 0.2 mL of HBSS supplemented with 5 �g/mL Hoechst 33342. The cells were placed in a glass-bottom dish (Matsunami, D141400) and observed using a confocal fluorescence microscope (Evident FV1000-D; IX81). To assess ER morphology, cells were incubated at 37°C for 15 minutes with 100 nM ER-Tracker Blue-White DPX or ERseeing, collected by centrifugation, resuspended in HBSS, and observed using a confocal microscope, as described above. In some cases, PlasMem Bright Red (1:100 dilution) was added to the staining solution to localize plasma membranes.

To assess cell size, cells were incubated at 37°C for 5 minutes with 5 μg/mL Hoechst 33342 and PlasMem Bright Red (1:100 dilution) in HBSS. After centrifugation, the cells were resuspended in HBSS and observed under a confocal fluorescence microscope. Fluorescence images were recognized using Cellpose-SAM for automatic segmentation and labelling of the region of interest (ROI) (76). ROIs corresponding to incorrectly segmented or edge-captured cells were manually excluded from the analysis. The area of each ROI was measured using Fiji (Image J2, version 2.16.0/1.54p).

### Electron microscopy.

Cells (1 × 10^6^) were fixed overnight at 4°C in 0.1 mL of 0.1 M phosphate buffer (pH 7.4) containing 2.5% glutaraldehyde and 2% paraformaldehyde. The samples were post-fixed with 2% OsO_4_ for 1 hour and block-stained with 2% aqueous uranyl acetate. After dehydration through a graded ethanol series, the samples were embedded in Epon 812 resin (TAAB Epon 812; EM Japan, 342). The sections were cut to a thickness of 70–80 nm using a UC6 ultramicrotome (Ultracut CUT N; Leica), stained with lead citrate and uranyl acetate, and examined using a JEM-1400Flash transmission electron microscope (JEOL).

### AlphaFold-based predictions.

Structural models of XKR and VPS13 family members were generated using the AlphaFold 3 online server (https://alphafoldserver.com) (27). For the VPS13A-XK complex, the full-length amino acid sequence of mouse VPS13A (NP_766616.2) was modeled in combination with that of mouse XK (NP_075989.1). Each AlphaFold prediction provided the 5 highest-ranked models, from which the top-ranked structure was selected for further analyses. Molecular graphics and analyses were performed using UCSF ChimeraX (version 1.10), developed by the Resource for Biocomputing, Visualization, and Informatics at UCSF (77).

### Statistics.

Statistical analysis was performed with GraphPad Prism (version 10.5.0). The significance of the differences was calculated using 1-way or 2-way ANOVA with Dunnett’s, Tukey’s, or Šídák’s multiple comparison test. Differences were considered significant when *P* < 0.05.

### Data availability.

Uncropped scans of all Western blots and all raw data used to create all graphs are in the supplemental material. All individual values represented in graphs are provided in the Supporting Data Values file or otherwise are available from the corresponding author upon request.

## Author contributions

SN and YU conceptualized the study; TS and HI devised the methodology; XL, YR, and CS conducted the investigation; XL, YR, and CS curated the data; XL, YR, HI, TS, YU, and SN reviewed and edited the manuscript; TS, YU, and SN acquired funding for the study.

## Conflict of interest

HI is on leave of absence from Chugai Pharmaceutical Co., Ltd. SN received a research grant from Chugai Pharmaceutical Co. Ltd.

## Funding support

- Japan Society for the Promotion of Science (grants 22K06102 and 25K09613 to TS and A21H04770 to SN).

## Supplementary Material

Supplemental data

Unedited blot and gel images

Supporting data values

## Figures and Tables

**Table 1:**
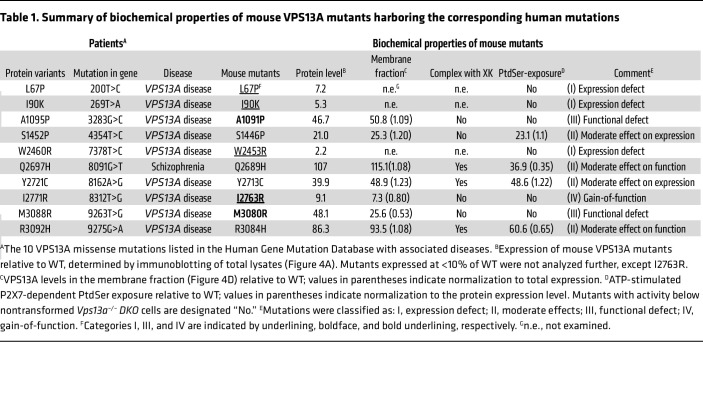
Summary of biochemical properties of mouse VPS13A mutants harboring the corresponding human mutations

**Table 2:**
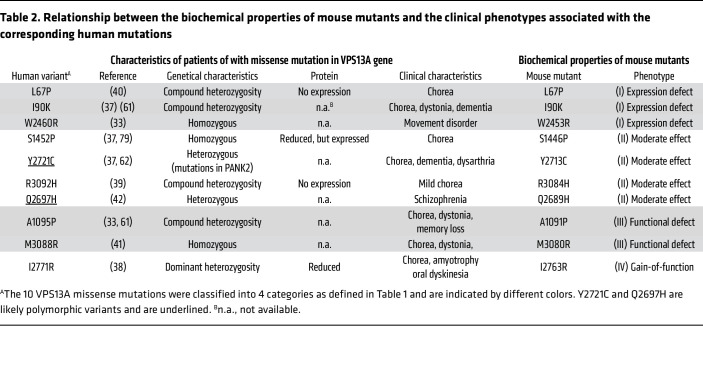
Relationship between the biochemical properties of mouse mutants and the clinical phenotypes associated with the corresponding human mutations

## Version 1 03/24/2026

In-Press Preview

## Version 2 05/15/2026

Electronic publication
