# Supplementary material for: Four subtypes of disease-causing missense mutations underlie pathogenic protein interactions in neurodegenerative VPS13A disease
Source: J Clin Invest. 2026 Mar 24;136(10):e200890. doi: 10.1172/JCI200890 (PMC13178662; doi:10.1172/JCI200890)
Supplement: Supplemental data [file jci-136-200890-s144.pdf]

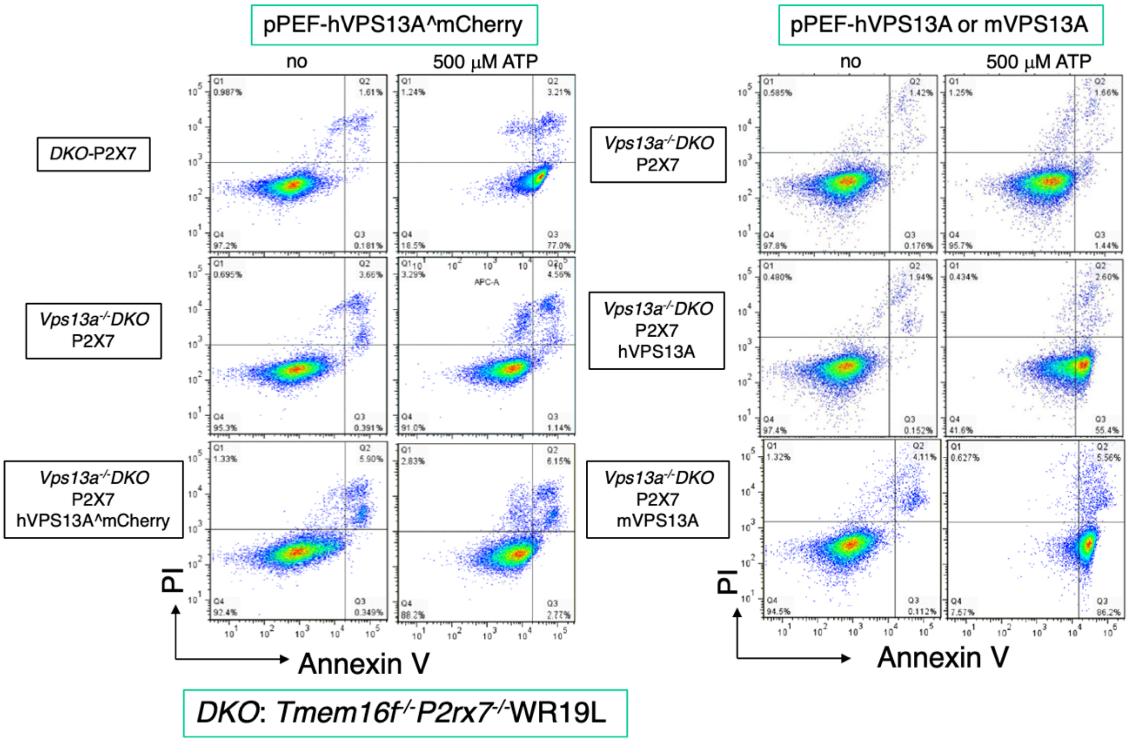

Supplemental Figure 1. **Comparison of the activity of internally mCherry-tagged human VPS13A and intact VPS13A in supporting ATP-stimulated P2X7-mediated PtdSer exposure.** Expression plasmids (pPEF-hVPS13A^mCherry, pPEF-hVPS13A, and pPEF-mVPS13A) were introduced into *Vps13a*$^{-/-}$*DKO*-P2X7 cells by electroporation, and stable transformants were established by culturing the cells in the presence of puromycin. *DKO*-P2X7, *Vps13a*$^{-/-}$*DKO*-P2X7, and transformants expressing hVPS13A^mCherry (left panels), human VPS13A, or mouse VPS13A (right panels) were incubated at 4°C with or without ATP for 5 min, stained with Cy5-Annexin and PI, and analyzed by flow cytometry.

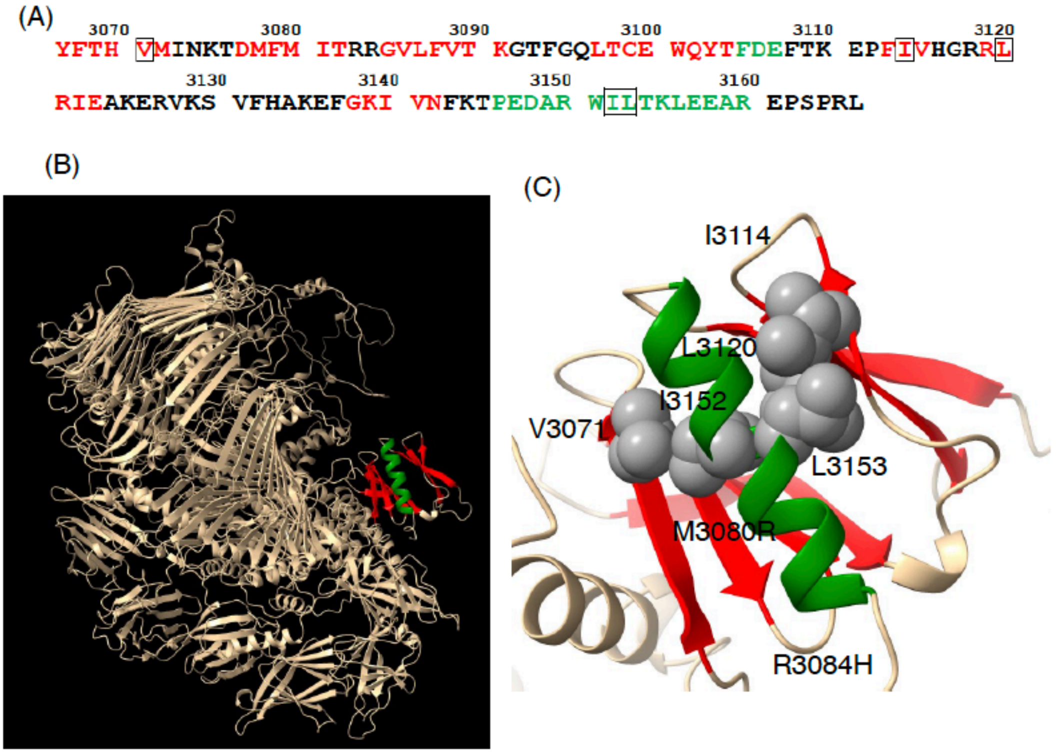

Supplemental Figure 2. **Knob-like structure in the C-terminal PH region of VPS13A.** (**A**) Amino acid sequence of the C-terminal region (residues 3067–3166) of mouse VPS13A. The β-strand and α-helical regions are highlighted in red and green, respectively. The residues involved in hydrophobic interactions are boxed. (**B**) AlphaFold 3-predicted tertiary structure of mouse VPS13A. The β-strand and α-helix in the C-terminal PH region are shown in red and green, respectively. (**C**) Hydrophobic interactions between the β-strand and α-helix. I3152 in the α-helix interacts with V3071 in the β-strand, and L3153 in the α-helix interacts with I3114 and L3120 in the β-strand, respectively.

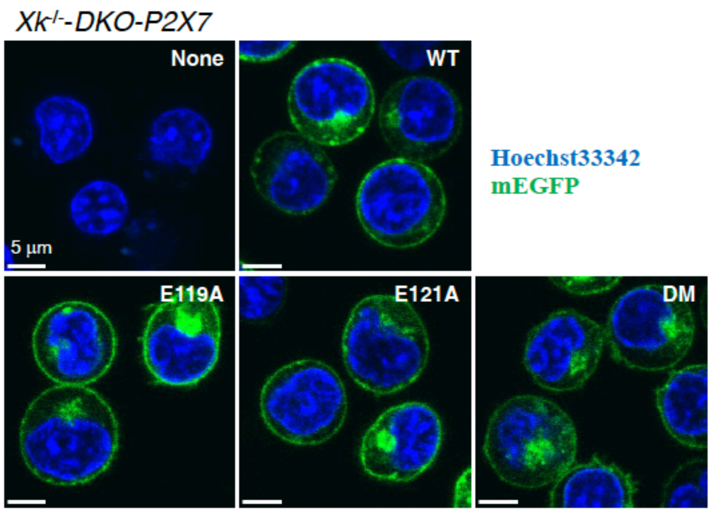

Supplemental Figure 3. Plasma membrane localization of mouse XK mutants.
Xk$^{-/-}$DKO cell transformants expressing C-terminally mEGFP-tagged mouse XK (WT, E119A, E121A, or DM [E119A/E121A]) were analyzed using confocal fluorescence microscopy.

```
XK   ------------------------------MKFPASVIASVFLFVAETAAALYLSSTYRSAGDRMWQVLTLLFSLMPCALVQ-FTLLFVHRDLSR------DRP
XKR2 MDRVYEIPEEPNVVPISSLEEDVIRGPNPRFTFPFSILFSTFLYCGEAASALYMVRIYRKNNETFWMTYTFSFFMFSSIMVQ-LTLIFVHRDLAK------DRP
XKR8 --------------------MPLSVHHHVALDVVVGLV-SILSFLLDLVADLWAVVQYVLLGRYLWAALVLVLLGQASVLLQLFSWLWLTADPTELHHSQLSRP

XK   LALLMHLLQLGPLYRCCEVFCIYCQ---SDQNEEPYVSITKKRQMPKDGLSEEVEKEVGQAEGKLITHRSAFSRASVIQAFLGSAPQLTLQLYITVLEQNITTG
XKR2 LSLFMHLILLGPVIRCLEAMIKYLTLWKKEGQEEPYVSLTRKK-MLIAGQEVLIEWEVGHSIRTLAMHRNAYKRMSQIQAFLGSVPQLTYQLYVSLISAEVPLG
XKR8 FLALLHLLQLGYLYRCLHGMHQGLSMCYQEMPSECD-------------------------LAYADFLSLDISMLKLFESFLEATPQLTLVLAIVLQNGQAEYY

XK   RCFIMTLSLLSIVYGALRCNILAIKIKYDEYEVKVKPLAYVCIFLWRSFEIATRVIVLVLFTSVLKIWVVAVILVNFFSFFLYPWIVFWCSGSPFPENIEKAL-
XKR2 RAVLMAFSLISVTYGATLCNMLAIQIKYDDYKIRLGPLEVLCITVWRTLEITSRLVILVLFSATLKLKAVPFLVLNFLIILFEPWVKFWRSGAQMPNNIEKNF-
XKR8 QWFGISSSFLGISWALLDYHR-SLRTCLPSKP-RLGRSSSAIYFLWNLLLLGPRICAIALFSAVF----PYYVALHFFSLWLVLLFWIWLQGTNFMPDSKGEWL

XK   SRVGTTIVLCFLTLLYAGINMFCWSAVQLKIDNPELISKSQNWYRLLIYYMTRFIENSVLLLLWYFFKTDIYMYVCAPLLILQL-LIGYCTGILFMLVFYQFFH
XKR2 SRVGTLVVLISVTILYAGINFSCWSAMQLKLADRDLVDKGQNWGHMGLHYSVRLVENVIMVLVFKYFGVKVLLNYCHSLIAVQL-IIAYLISIGVMLLFFQYLH
XKR8 YRVTMALILYFSWFN---------------------VSGGRTRGRAVIHLIFIFSDSVLLVTTSWVTHGTWLPSGISLLMWVTIGGACFFLGLALRVIYYLWLH

XK   PCKKLFSSSVSESFRALLRCACWSSLRRKSSEPVGRIDTDLKACTEQDVMPTTSKVIPEATDIWTAVDLCSA
XKR2 PLRSLFTNNVVD----YLHCIC---CRRPRPERVENSETSCEADTTQSIV----------------------
XKR8 PSCSWDPDLVDGTLGLLSPHRPPKLIYNRRATLLAENFFAKAKARAVLTEEVQLNGVL--------------
```

Supplemental Figure 4. Amino acid sequence alignment of the mouse XKR family. The amino acid sequences of mouse XK (UniProt, Q9QXY7), XKR2 (UniProt, Q5GH68), and XKR8 (UniProt, Q8C0T0) were aligned to obtain maximal homology by introducing gaps (-). The putative transmembrane regions in XK are shaded. The β-hairpins in XK and XKR2 are highlighted in yellow. The residues that are identical between mouse XK and XKR2 or among all three members are indicated in red. The glutamic acid residues conserved between XK (E119 and E121) and XKR2 (E151 and E153) are highlighted in green. The caspase recognition site in XKR8 is underlined.

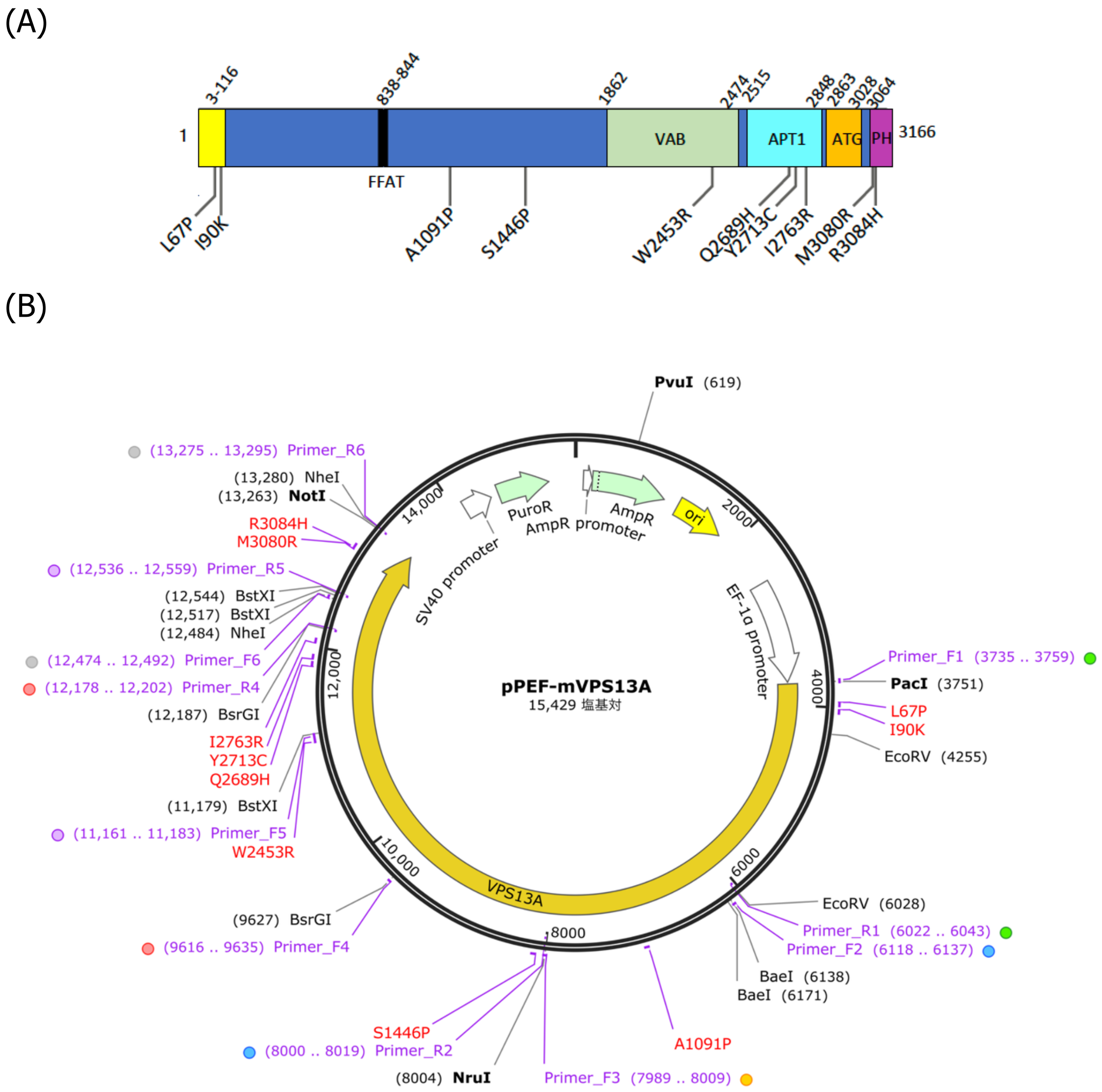

Supplemental Figure 5. **Expression vector construction for mouse VPS13A mutants.**
(**A**) Schematic representation of the domain structure of the mouse VPS13A. The N-terminal Chorein_N (aa 3–116), VAB (VPS13 adaptor binding, aa 1862–2474), APT1 (aberrant pollen transmission 1 domain, aa 2515-2848), ATG_C (autophagy C-terminal, aa 2863–3028), and PH (pleckstrin homology, aa 3064–3166) domains are shown. The FFAT motif (two phenylalanine in an acidic tract, aa 838-844) is also shown. The positions of point mutations introduced into mouse VPS13A are indicated. (**B**) Construction strategy for the mutant expression vectors. pPEF-Vps13a carries a 9498 bp DNA fragment encoding mouse VPS13A, preceded by a Kozak sequence, inserted between the PacI and NotI sites of pPEF-BOS-EX vector. Ten missense mutants (L67P, I90K, A1091P, S1446P, W2453R, Q2689H, Y2713C, I2763R, M3080R, and R3084H) are highlighted in red. The forward (F1–F6) and reverse (R1–R6) primers used for PCR mutagenesis are shown in purple. Restriction enzyme sites (PacI, EcoRV, BaeI, NruI, BsrGI, BstXI, and NheI) used for In-Fusion cloning are indicated by their nucleotide positions.

(A)

```
                                       β1                               β2
mVPS13A(3063) AKYKYFTHVMINKTDMFMITRRGVLFVTKGTFG-QLTCEWQYTFDEFTKEPFIVHGRRLRIEAKER
mVPS13C(3625) EAYQFHCAVPGNKRAVLMITNRRALFIKEVEILGHMSVDWQCLFEDFVCPPEVSEN-LLKISVKEQ
mVPS13D(4232) IQDEFFIAVENIDSYCVLISSKAVYFLKSGDYVDREAIFLEVKYDDLYHCLVSKDHGKVYVQVTKK
 yVPS13(3049) MNDEYLSHVILPGKELAVIVSMQHIAEVQMATQ---ELMWSTGYPS--IQGITLERSGLQIKLKSQ
 dVPS13(3205) TDNFIHCEEIIQKSEYLVVTNYRVMYVQRNEMFGVWTSLWSYLWNEISSVAATARGVQFTVKTDGK

                    β2                    α
mVPS13A(3128) ------VKSVFHAKEFGKIVNFKTPEDARWILTKLEEAREPSPRL---------------------
mVPS13C(3690) ---GLFHKKDSANIGHLRKIYLKDPITAKRAFDAIESAQSARQQQKLMRQSSVKLLRPQGPS----
mVPS13D(4298) AANSSSGVSIPGPSHQKPMVHVKSEVLAVKLSQEINYAKSLYYEQQLMLRLSENQEQLELDS----
 yVPS13(3110) ---------------SEYFIPISDPEERRSLYRNIAIAVREYNKYCEAIL----------------
 dVPS13(3271) -----KVLGLFSSKESPRKLVLVADERKRDALVDIIESQRSDPNPLRATIAYPAHN----------
```

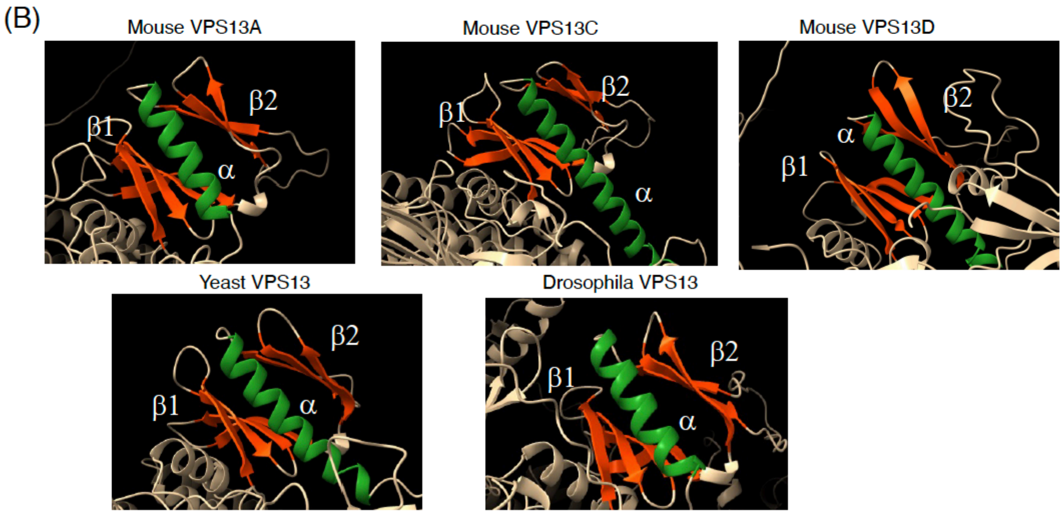

Supplemental Figure 6. The conserved C-terminal region of the VPS13 family. (**A**) Amino acid sequences of the C-terminal regions of mouse VPS13A (UniProt: Q5H8C4), VPS13C (UniProt: Q8BX70), VPS13D (UniProt: B1ART2), *S. cerevisiae* VPS13 (UniProt Q07878), and *Drosophila* VPS13 (UniProt: A1Z713) were aligned. Two clusters of β-strands (β1 and β2) and α αν-helix are highlighted in orange and green, respectively. (**B**) AlphaFold 3-predicted β-α-β structure of Mouse VPS13A, mouse VPS13C, mouse VPS13D, *S. cerevisiae* VPS13, and *Drosophila* VPS13. The α-helix and β-strands are shown in green and red, respectively.

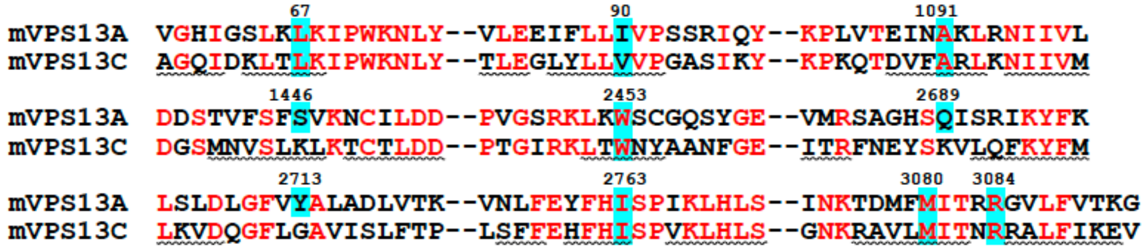

Supplemental Figure 7. Conservation of essential residues between mouse VPS13A and VPS13C. The amino acid sequence surrounding the mutated 10 residues (highlighted in sky blue with the residue number above the line) of mouse VPS13A (UniProt: Q5H8C4) was aligned with the corresponding region of mouse VPS13C (UniProt: Q8BX70). Identical residues between the two proteins are shown in red. β-strand regions are indicated by wavy underlines.

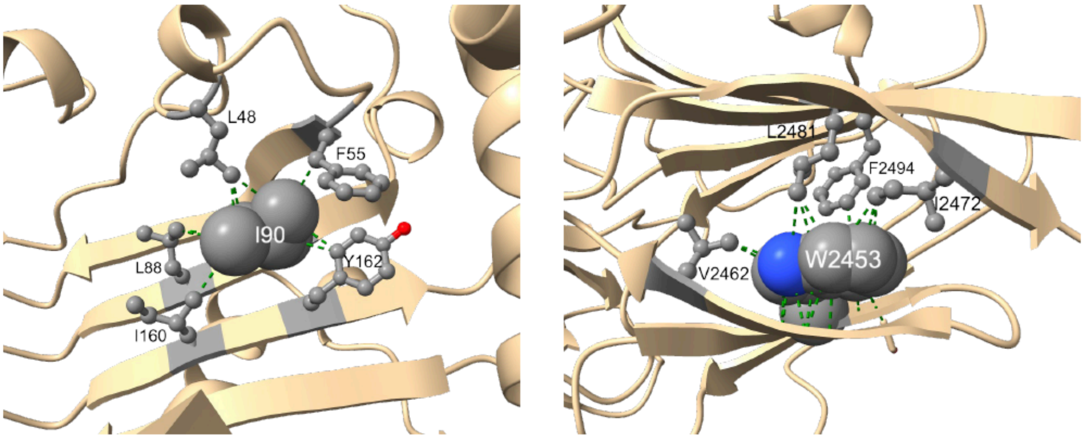

Supplemental Figure 8. Hydrophobic interactions between I90 and W2453 and the surrounding residues. The local structures of I90 and W2453 in mouse VPS13A are shown. The residues interacting with I90 and W2453 were predicted using the Contact Command of Chimera X and are shown as colored elements.

Supplementary Table 1. Primers used for deleting mCherry from human VPS13A^mCherry

	Forward primer[*1]	Reverse joint primer[*2]
upstream fragment	5'-TGTTTTAATGTAAATGCTCAG (BlpI)	5'-CCACAGTTGCTCCTCCTGAATGGTGTGAAG

	Forward joint primer	Reverse primer
downstream fragment	5'-TTCAGGAGGAGCAACTGTGGTGACAGCTGC	5'- CTTATAATATTCTCCAATTTAAATTC (SwaI)

*1 The recognition sites for restriction enzyme (BlpI and SwaI) in the forward and reverse primers are underlined.

*2 The complementary region in the reverse and forward mutagenesis primers are underlined.

Supplementary Table 2. Primers used for mutagenesis of mouse VPS13A

Mutagenesis Primers for mouse VPS13A mutants

mutant	forward primer	reverse mutagenesis primer	forward mutagenesis primer*	reverse primer
L67P	Primer_F1	5’-ATTTTAgGTTTAAGACTACCTATATGACCAAC-3’	5’-TCTTAAACcTAAAATTCCATGGAAAAACCTTT-3’	Primer_R1
I90K	Primer_F1	5’-GGCACTtTGAGTAAAAAAATTTCTTCCAAAAC-3’	5’-TTTACTCAaAGTGCCTTCTTCTAGAATACAG-3’	Primer_R1
A1091P	Primer_F2	5'-GCTTTGgATTTATTTCAGTGACTAGAGGTTTC-3'	5'-AAATAAATcCAAAGCTAAGGAATATAATTGTG-3'	Primer_R2
S1446P	Primer_F3	5'-TTACTGgGAAAGAAAAGACTGTAGAATCATCT-3'	5'-TTTCTTTCcCAGTAAAAAACTGTATTTTAGATGA-3'	Primer_R4
W2453R	Primer_F4	5’-AGCTCCtCTTCAGCTTTCTAGAGCCCACTGG-3’	5’-AGCTGAAGaGGAGCTGTGGGCAAAGCTATGG-3’	Primer_R4
Q2689H	Primer_F5	5’-TGATATgTGGGAATGTCCTGCAGATC-3’	5’-CATTCCCAcATATCACGCATTAAGTATTTCAAAG-3’	Primer_R5
Y2713C	Primer_F5	5’-AGGGCAcACACAAATCCAAGATCCAAACTGAG-3’	5’-ATTTGTGTgTGCCCTAGCAGACCTTGTCAC-3’	Primer_R5
I2763R	Primer_F5	5’-GGAGATcTATGAAAATATTCAAAGAGATTGACTTGTG-3’	5’-TTTTCATAgATCTCCTATCAAGTTGCACTTGAG-3’	Primer_R5
M3080R	Primer_F6	5'-GTTATCcTGAACATATCTGTTTTATTGATCAT-3'	5'-TATGTTCAgGATAACAAGACGTGGCGT-3'	Primer_R6
R3084H	Primer_F6	5’-tGTCTTGTTATCATGAACATATCTGTTTTATTGATCATG-3’	5’-CATGATAACAAGACaTGGCGTGTTGTTCGTAACAAAGG-3’	Primer_R6
R3119A	Primer_F6	5’-CGCAGTgcTCTCCCATGGACAATGAACGG-3’	5’-TGGGAGAgcACTGCGCATTGAAGCCAAGG-3’	Primer_R6
R3121A	Primer_F6	5’-TCAATGgcCAGTCTTCTCCCATGGACAATG-3’	5’-AAGACTGgcCATTGAAGCCAAGGAACGGGTG-3’	Primer_R6
R3119A/ R3121A	Primer_F6	5’-ATGgcCAGTgcTCTCCCATGGACAATGAACGG-3’	5’-GAGAgcACTGgcCATTGAAGCCAAGGAACGGG-3’	Primer_R6
R3127Δ	Primer_F6	5'-TTCACtcaTTCCTTGGCTTCAATGCGC-3'	5'-CAAGGAAtgaGTGAAGTCTGTATTCCATGCC-3'	Primer_R6
E3136Δ	Primer_F6	5'-TTCCAAA^CTTTGGCATGGAATACAGACTTCA-3'	5'-TGCCAAAG^TTTGGAAAAATCGTTAACTTCAAGA-3'	Primer_R6

*Complementary sequences are underlined, while mutated nucleotides are shown in lower case letters. Caret symbols indicate positions of nucleotide deletions.

Forward and reverse junction primers

Junction Primers*			
Primer_F1	5’-CGTGAGGAATTCTTAATTAAGCCAC-3’ (PacI)	Primer_R1	5’-TTTGGGCATCTTGATATCCATG-3’ (EcoRV)
Primer_F2	5'-GTTGGGAAGCATTCCAAAGC-3'	Primer_R2	5'-TCCAGAGAAGGATCGCGAAC-3' (NruI)
Primer_F3	5'-CCTTTACAGATGTTCGCGATC-3' (NruI)		
Primer_F4	5’-GGGACGCCGTCTGTACACTG-3’ (BsrGI)	Primer_R4	5’-AACTACATCTTGTACATCTGTGAGG-3’ (BsrGI)
Primer_F5	5’-TATGATGACGCCCATAAGTGTGG-3’ (BstXI)	Primer_R5	5’-GTAGTCTTCATCCATGGTCATGGC-3’ (BstXI)
Primer_F6	5'-CCGTTGGTGGGCTAGCTGG-3' (NheI)	Primer_R6	5'-TAGAGTCGACGCTAGCGGATC-3' (NheI)

*The recognition site for restriction enzyme is underlined.

Supplementary Table 3. Primers used for mutagenesis of mouse XK

Mutagenesis Primers for XK mutants

mutant	forward primer	reverse mutagenesis primer	forward mutagenesis primer	reverse primer
E119A	Primer_F7	5'-TCTTTCgCCACCTCCTCTGAAAGGCC-3'	5'-GGAGGTGGcGAAAGAGGTTGGCCAGGC-3'	Primer_R7
E121A	Primer_F7	5'-CCAACCgCTTTCTCCACCTCCTCTGAAAGGC-3'	5'-GGAGAAAGcGGTTGGCCAGGCAGAAGG-3'	Primer_R7
E119A/E121A	Primer_F7	5'-CCAACCgCTTTCgCCACCTCCTCTGAAAGGCC-3'	5'-GTGGcGAAAGcGGTTGGCCAGGCAGAAGG-3'	Primer_R7

*Complementary sequences are underlined, while mutated nucleotides are shown in lower case letters.

Forward and reverse junction primers

Junction Primers			
Primer_F7	5'-ATATGGATCCGAGATGAAATTCCCGGCCTCGGT-3' (BamHI)	Primer_R7	5'-ATATGAATTCAGCAGAGCACAGATCAACAG-3' (EcoRI)

*The recognition site for restriction enzyme is underlined.

Supplementary Table 4. Primers used for mutagenesis of mouse XKR2

Mutagenesis Primers for XKR2 mutants

mutant	forward primer	reverse mutagenesis primer	forward mutagenesis primer	reverse primer
E151A	Primer_F8	5'-TCCCATgCTATCAGCACCTCCTGGCCAG-3'	5'-GCTGATAGcATGGGAGGTGGGCCACTC-3'	Primer_R8
E153A	Primer_F8	5'-CCCACCgCCCATTCTATCAGCACCTCCTGG-3'	5'-AGAATGGGcGGTGGGCCACTCCATCCG-3'	Primer_R8
E151A/E153A	Primer_F8	5'-ACCgCCCATgCTATCAGCACCTCCTGGCC-3'	5'-GATAGcATGGGcGGTGGGCCACTCCATCCG-3'	Primer_R8

*Complementary sequences are underlined, while mutated nucleotides are shown in lower case letters.

Forward and reverse junction primers

Junction Primers			
Primer_F8	5'-ATATGGATCCACAATGGACAGAGTTTATGAAAT-3' (BamHI)	Primer_R8	5'-ATATGAATTCGACAATACTTTGTGTTGTGT-3' (EcoRI)

*The recognition site for restriction enzyme is underlined.
