# Supplementary material for: Four subtypes of disease-causing missense mutations underlie pathogenic protein interactions in neurodegenerative VPS13A disease
Source: J Clin Invest. 2026 Mar 24;136(10):e200890. doi: 10.1172/JCI200890 (PMC13178662; doi:10.1172/JCI200890)

Full unedited blot for Figure 1B (none, WT, R3127Δ/CBB stain)

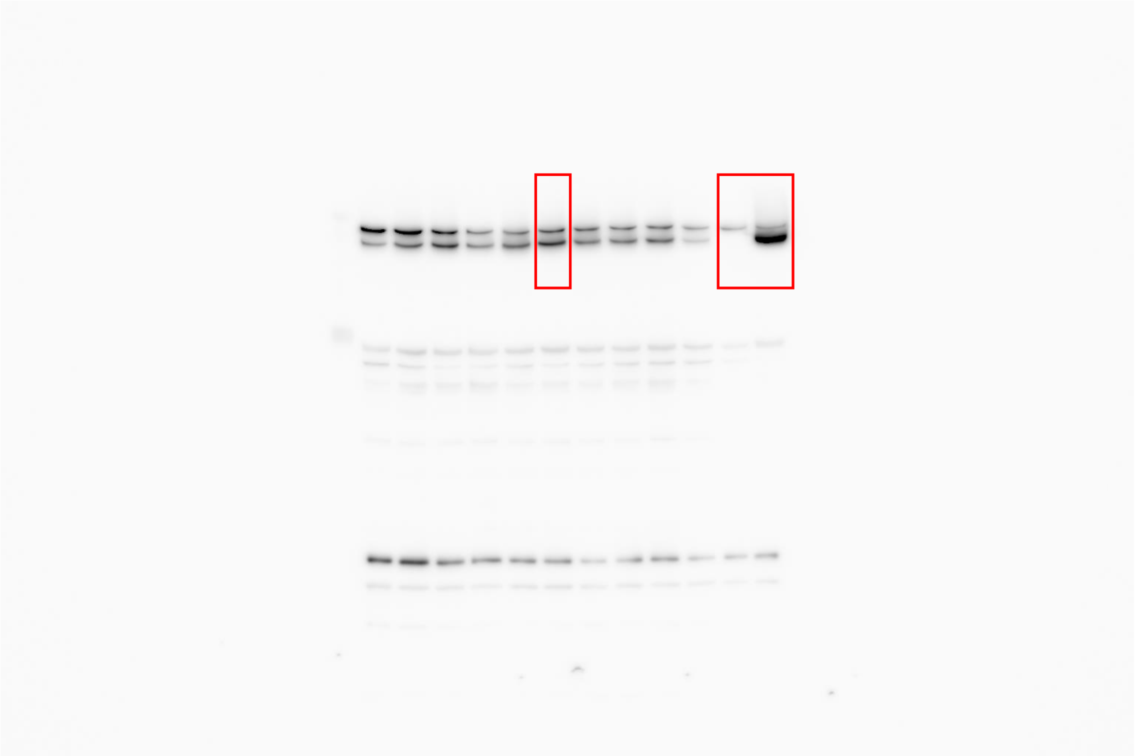

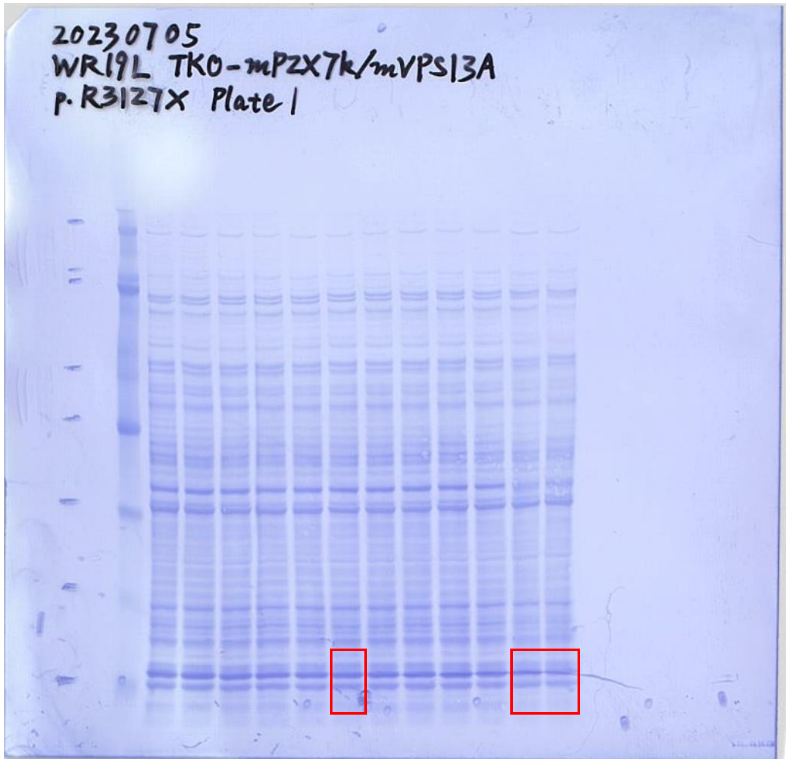

Full unedited blot for Figure 1B (E3136Δ/CBB stain)

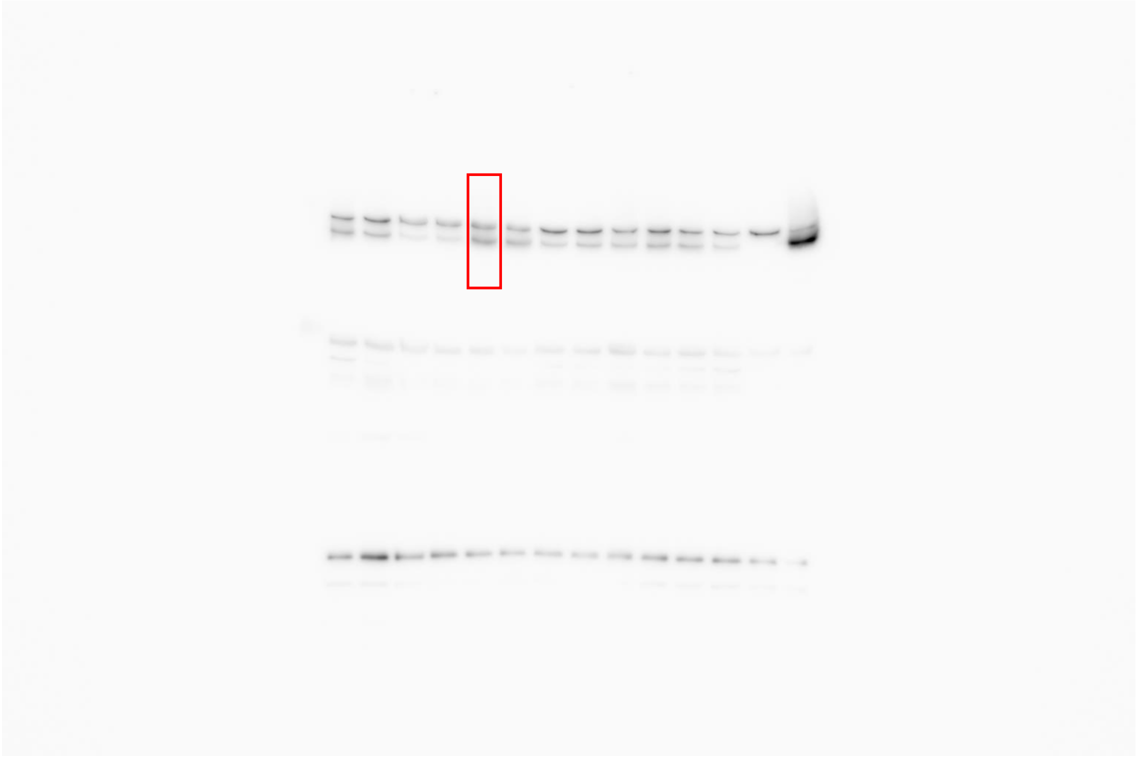

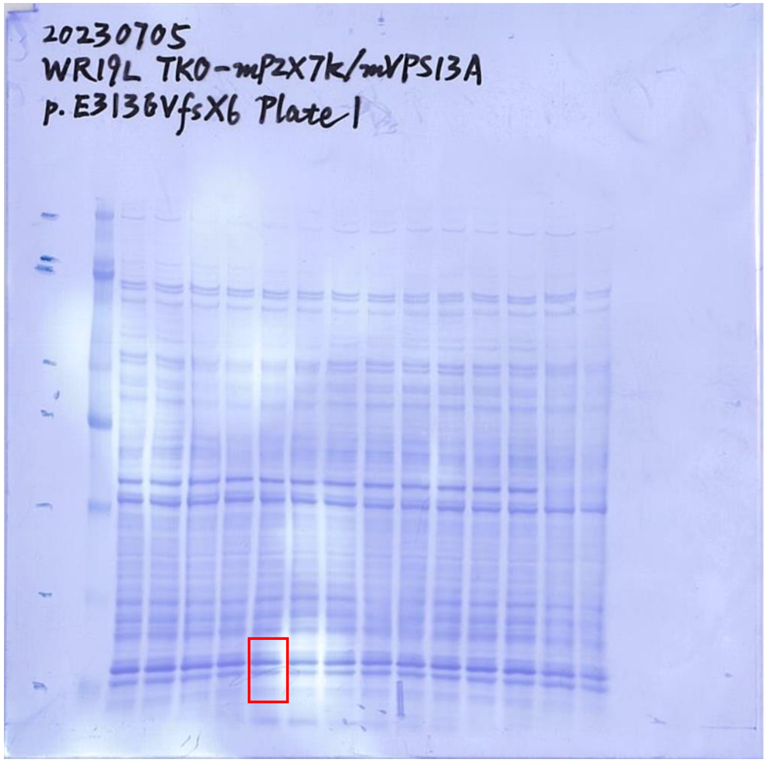

Full unedited blot for Figure 1C (VPS13A)

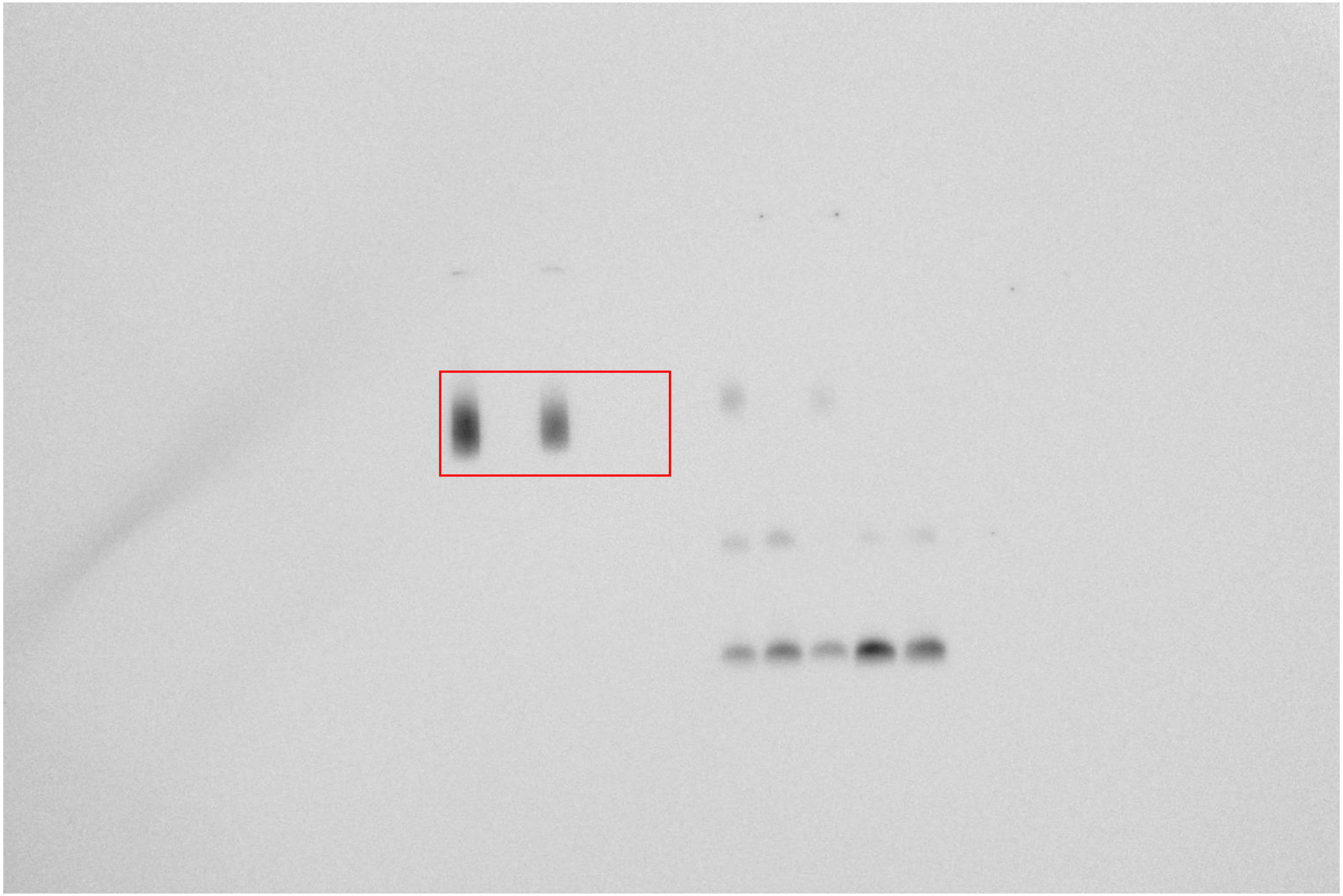

Full unedited blot for Figure 1C (XK)

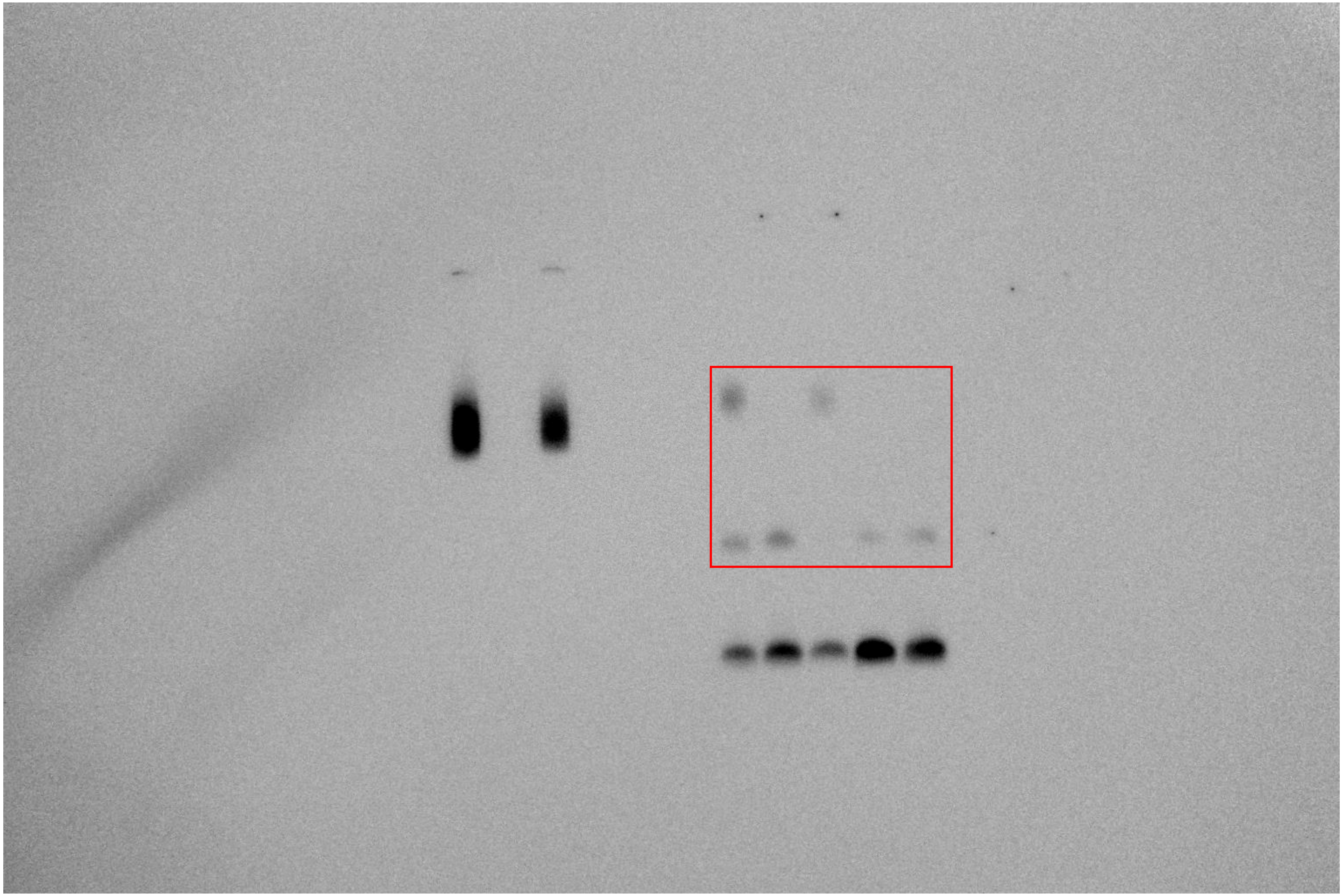

Full unedited blot for Figure 1C (Gold stain)

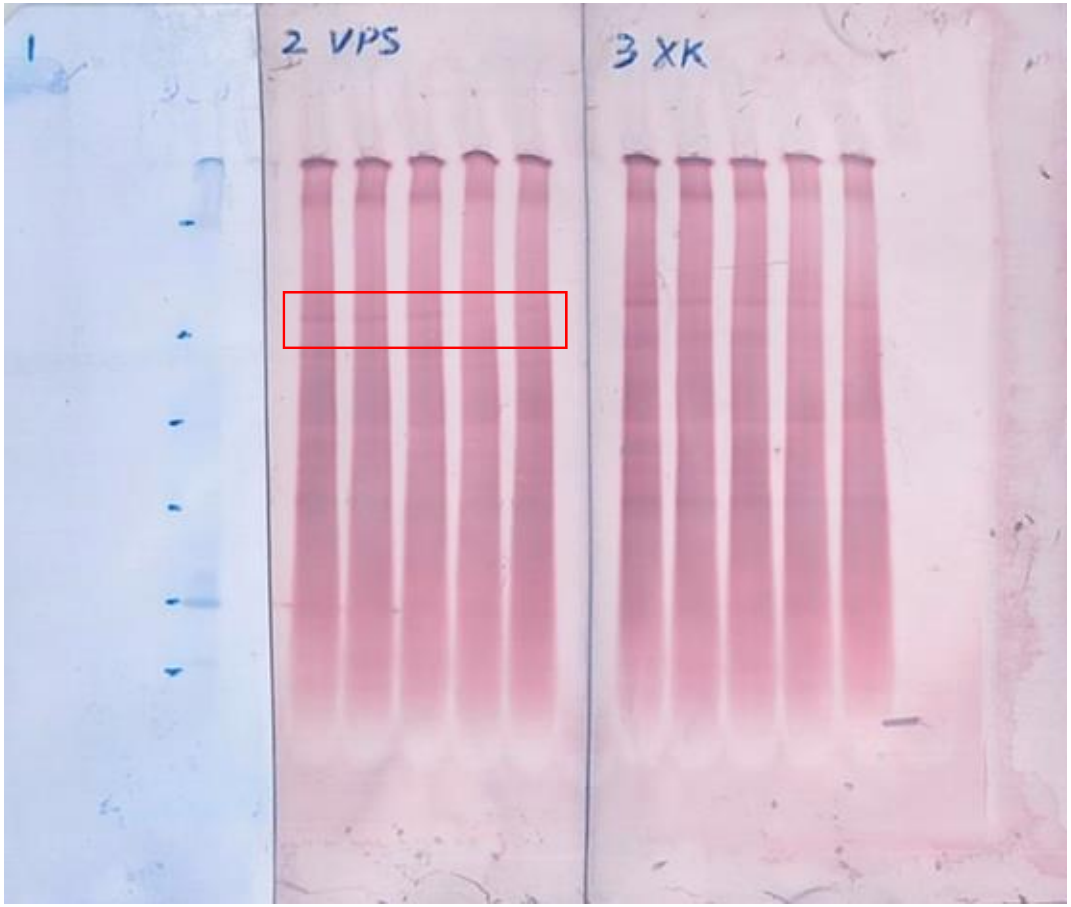

Full unedited blot for Figure 2B

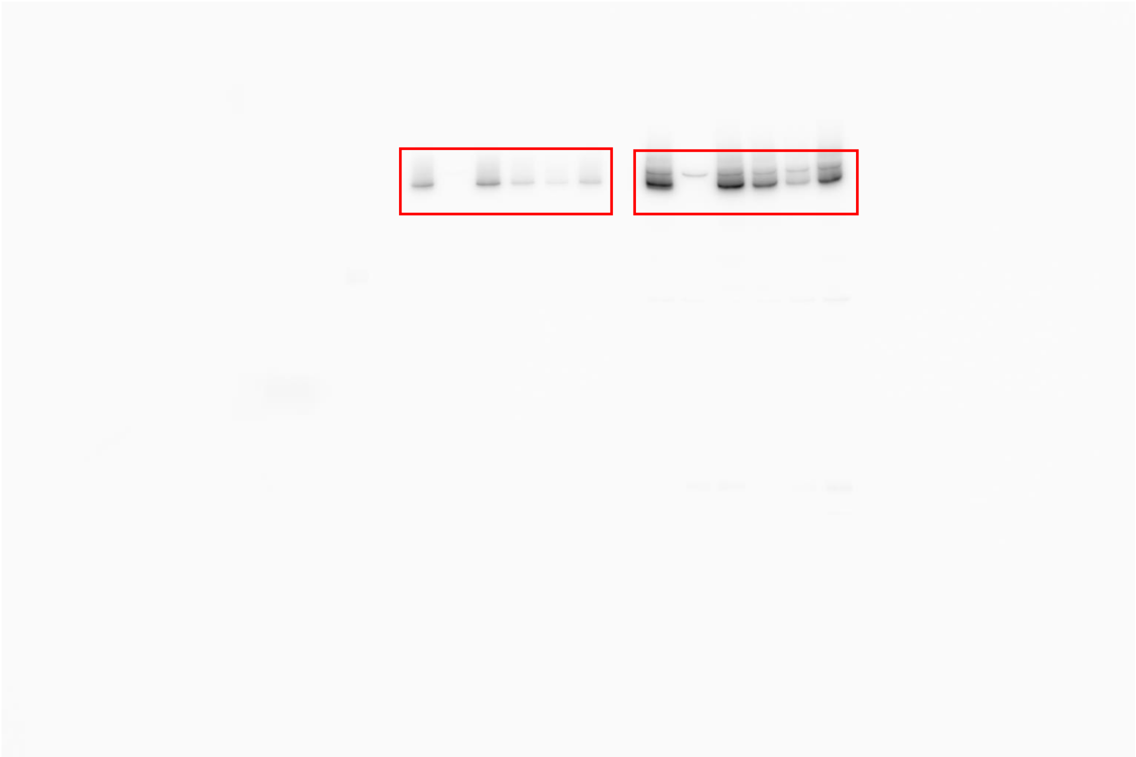

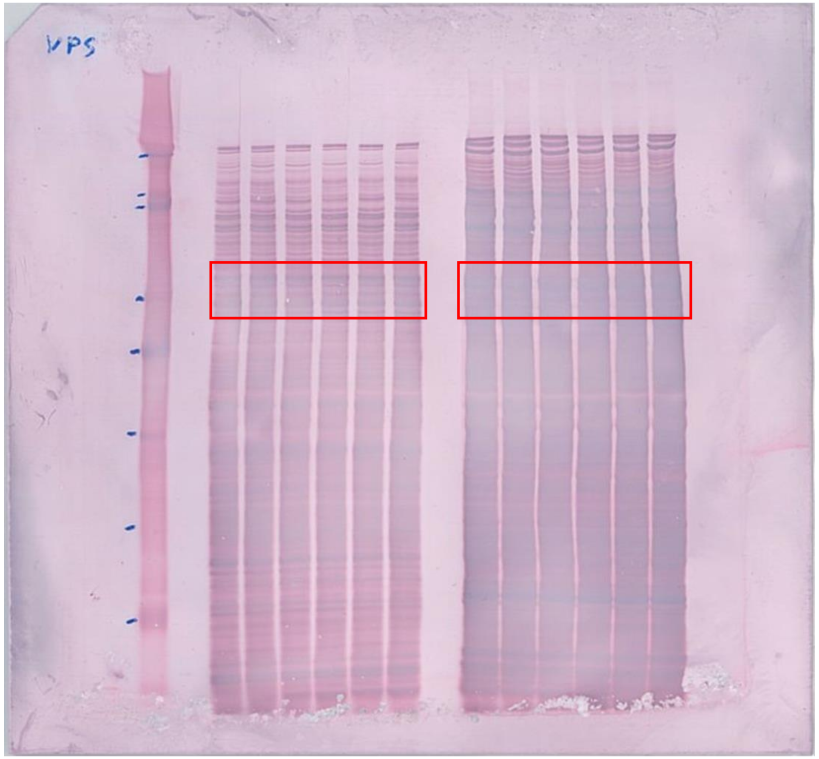

Full unedited blot for Figure 2C (VPS13A)

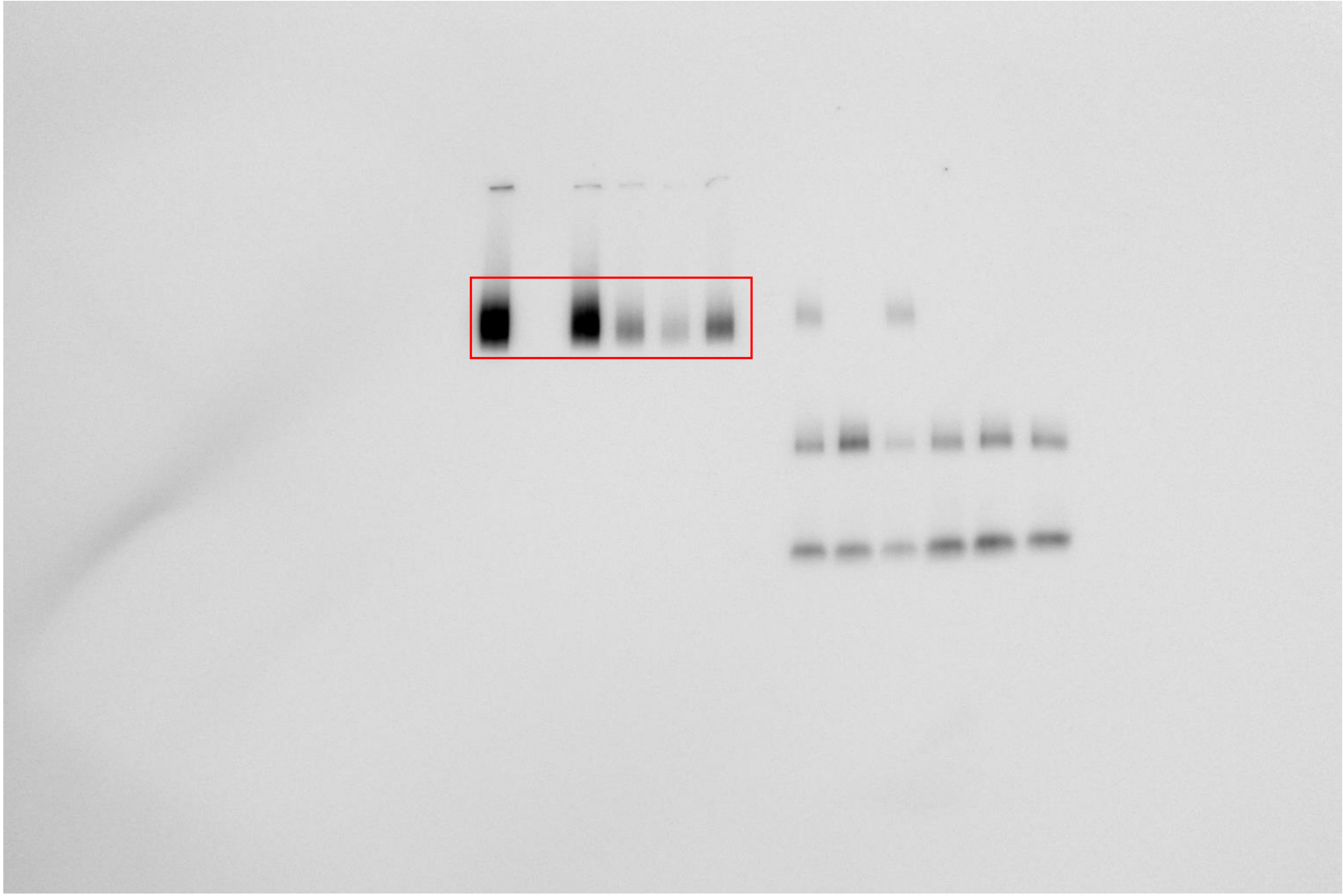

Full unedited blot for Figure 2C (XK)

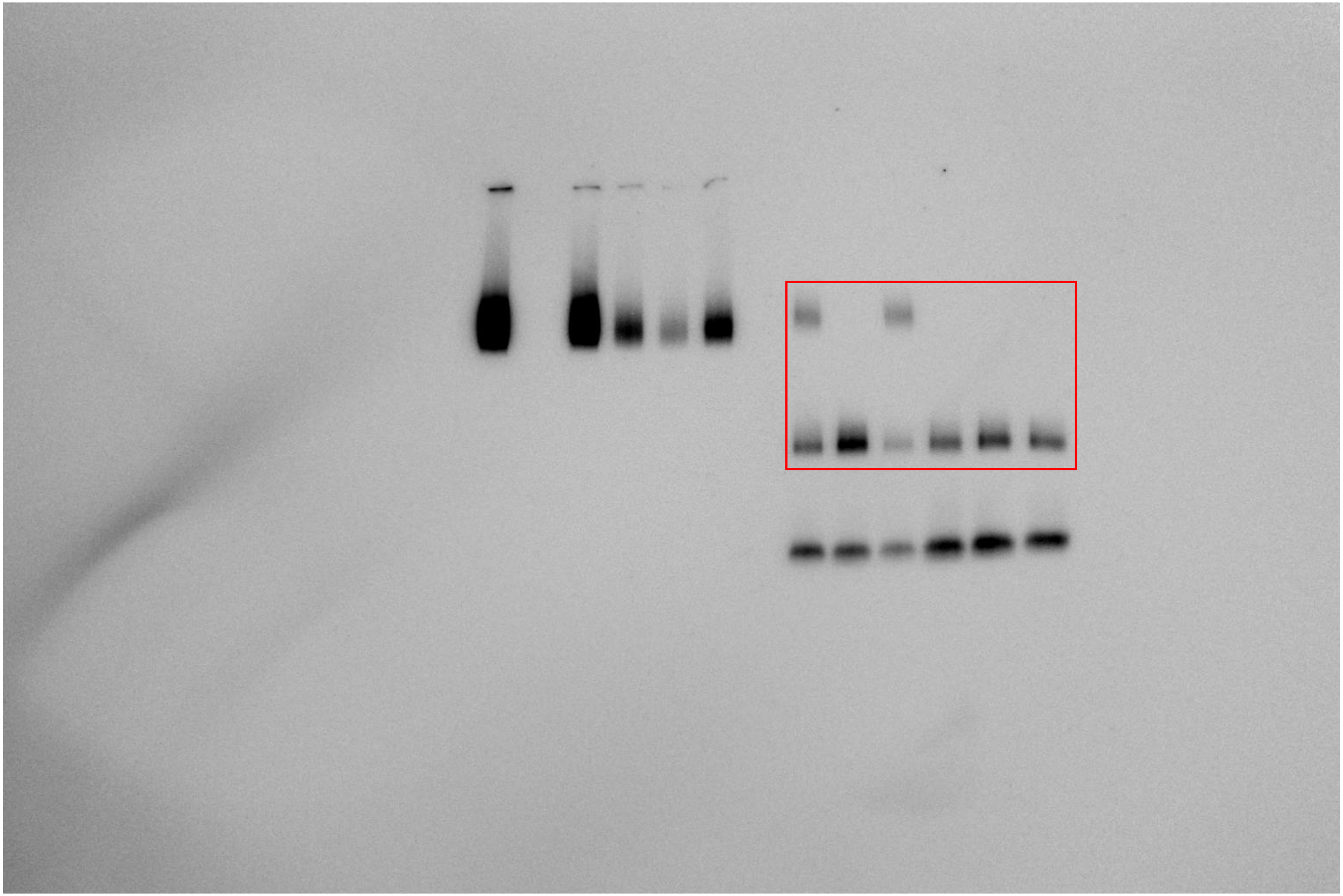

Full unedited blot for Figure 2C (Gold stain)

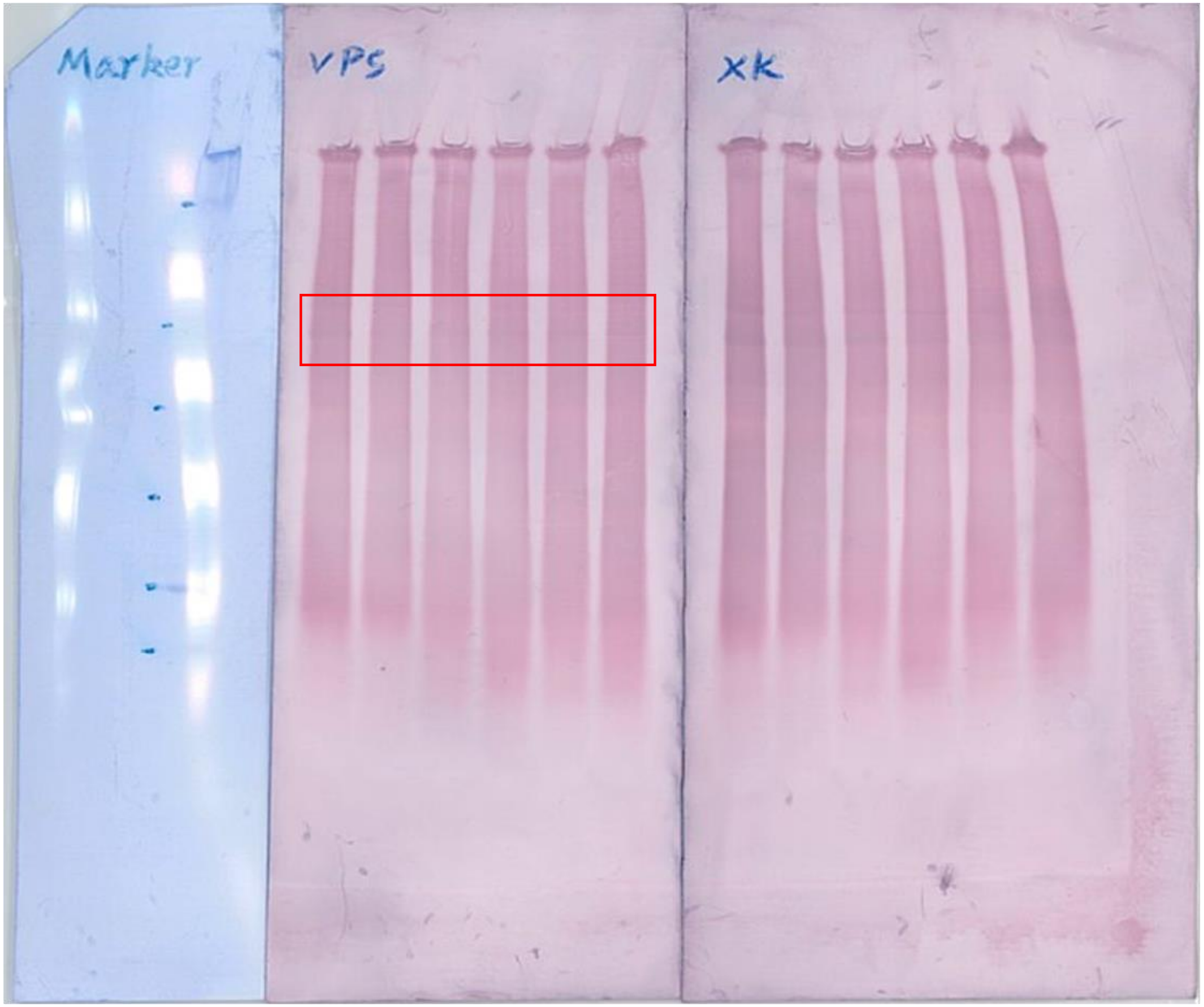

Full unedited blot for Figure 2E (GFP/CBB stain)

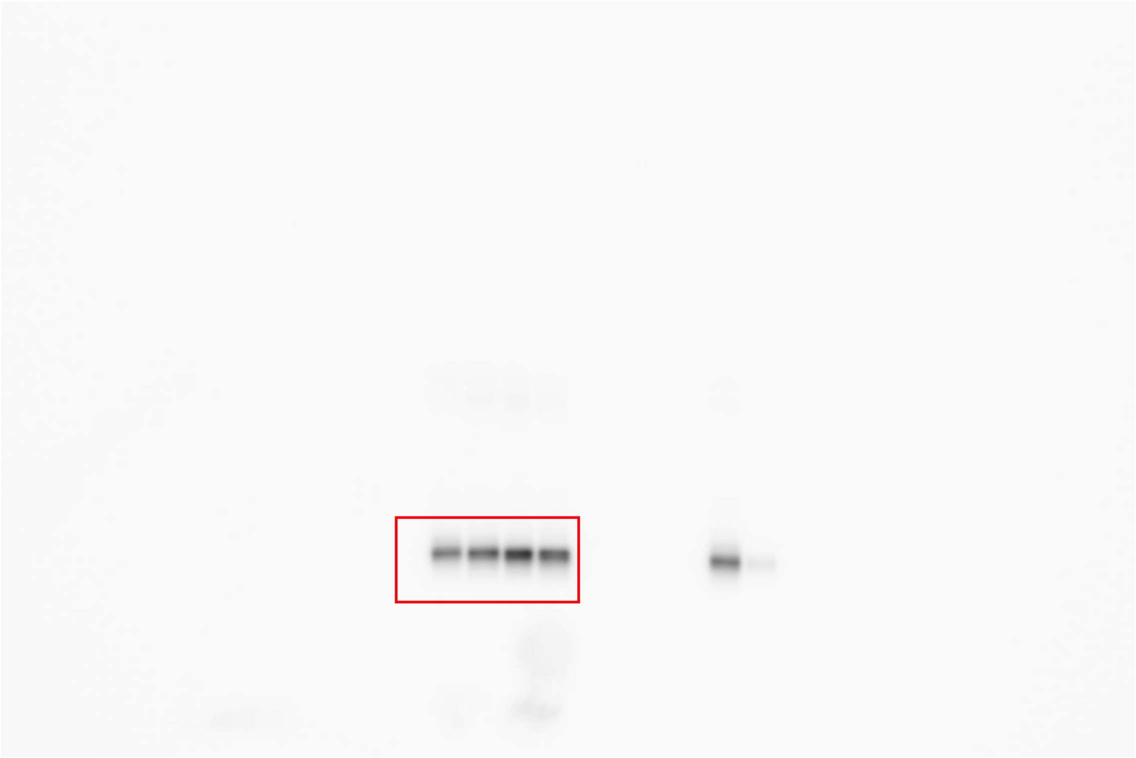

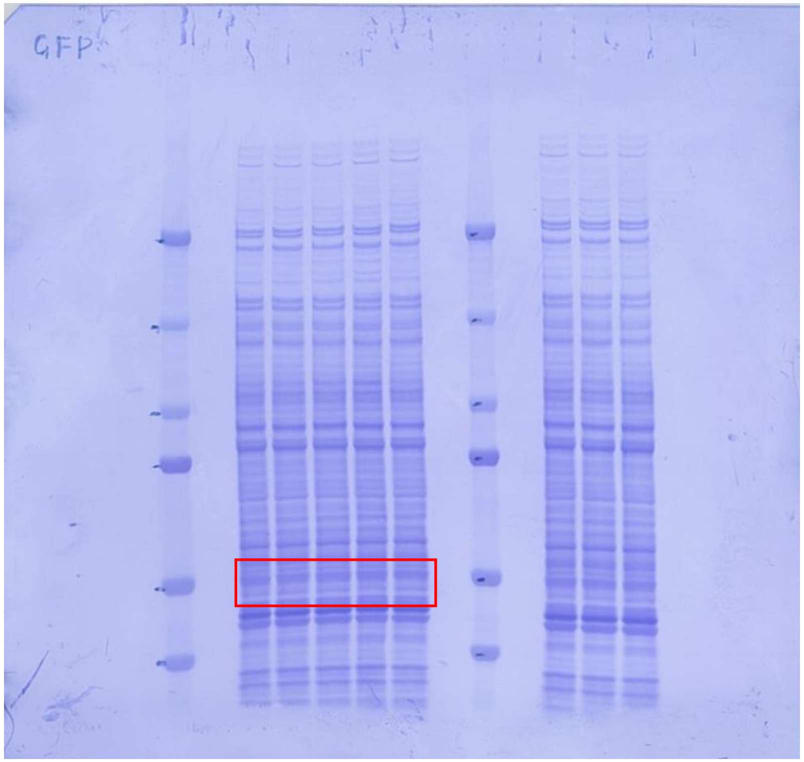

Full unedited blot for Figure 2F (BN-PAGE)

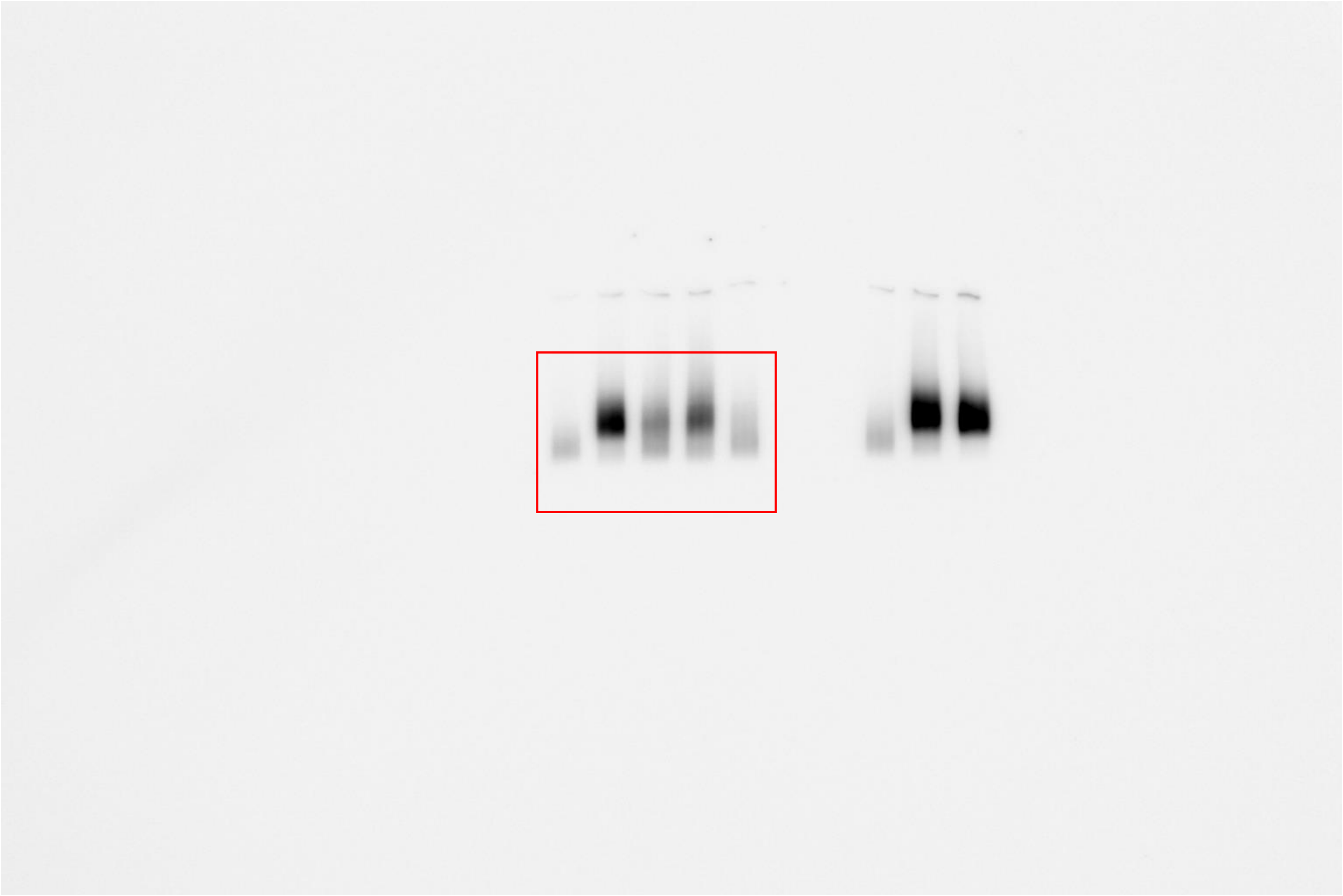

Full unedited blot for Figure 2F (BN-PAGE/Gold stain)

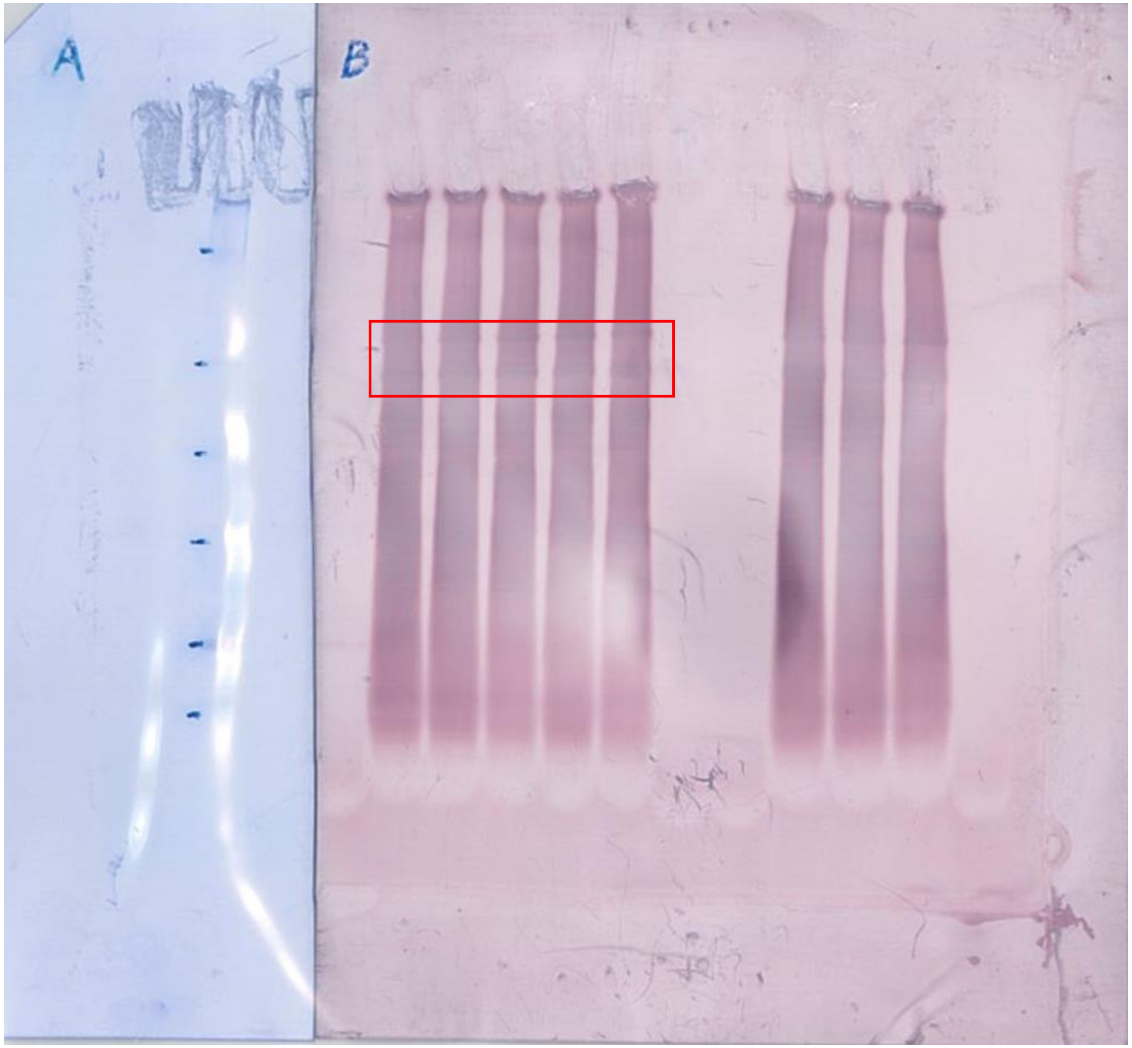

Full unedited blot for Figure 2F (SDS-PAGE)

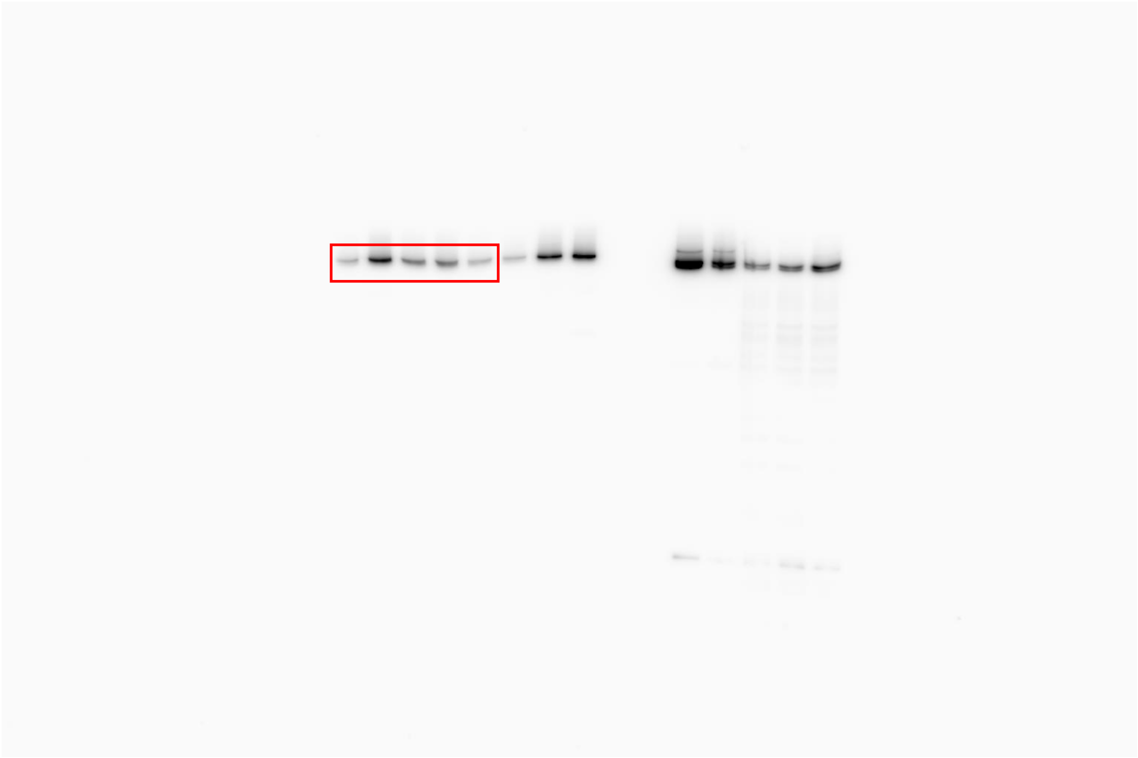

Full unedited blot for Figure 3B (SDS-PAGE/CBB Stain)

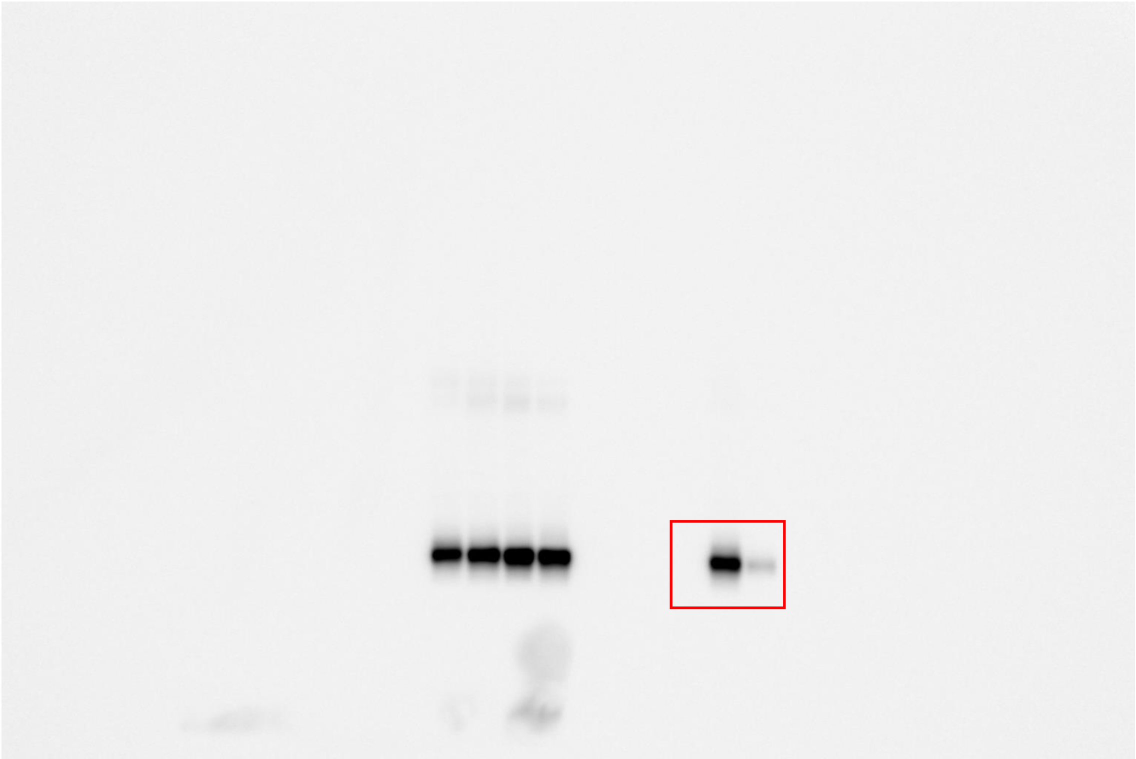

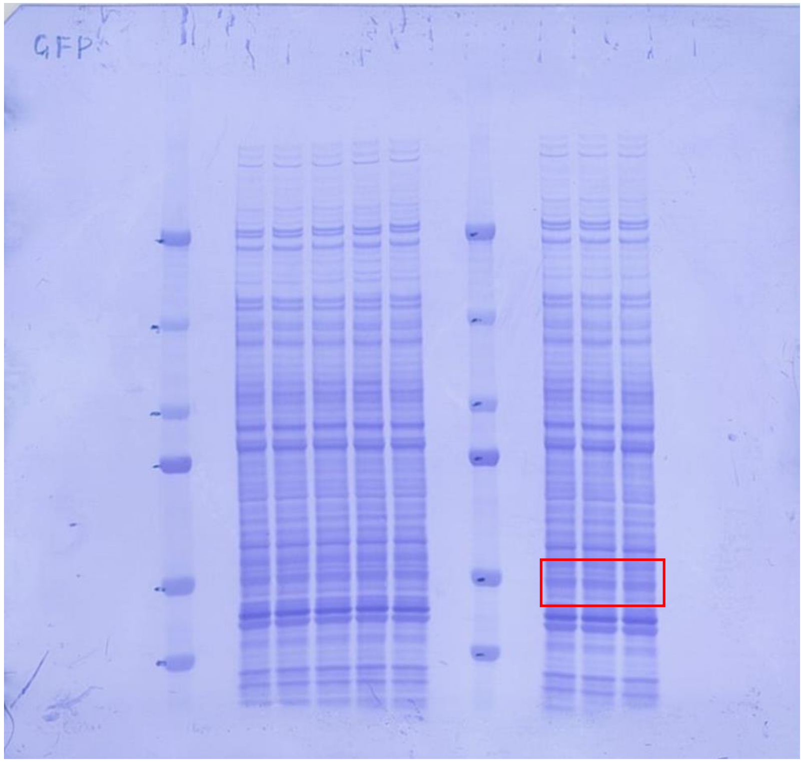

Full unedited blot for Figure 3D (VPS13A)

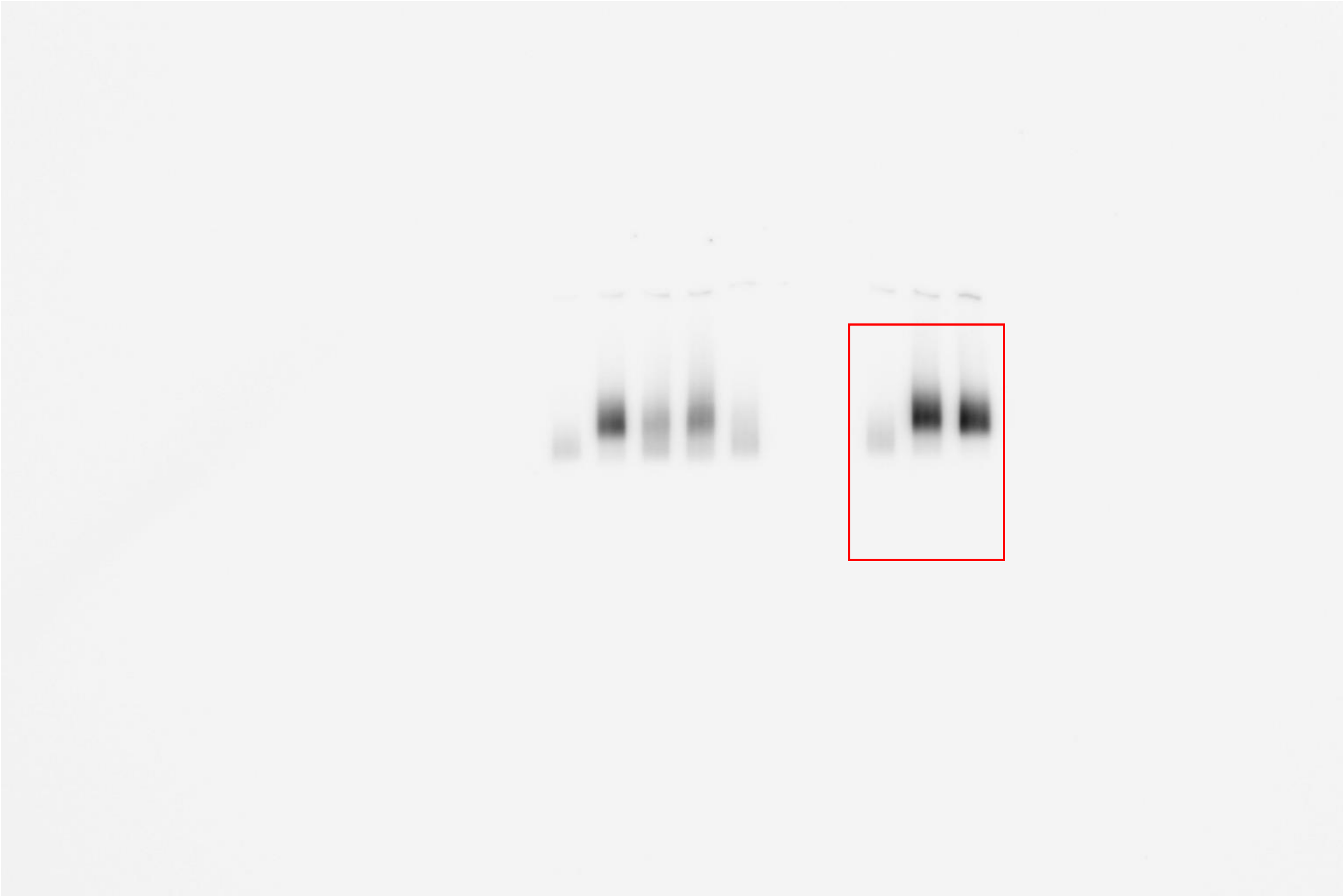

Full unedited blot for Figure 3D (VPS13A/Gold stain)

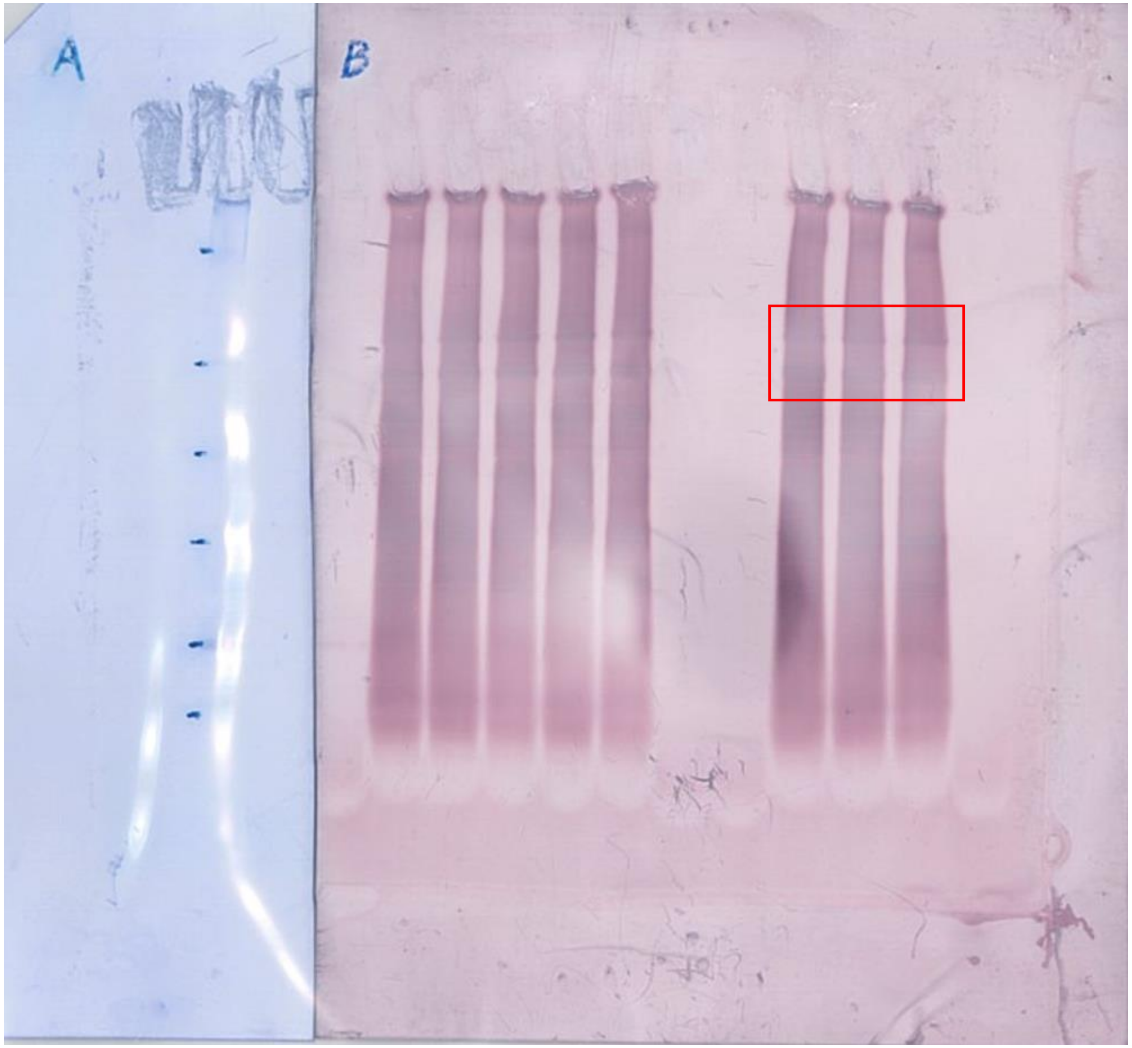

Full unedited blot for Figure 3D (GFP)

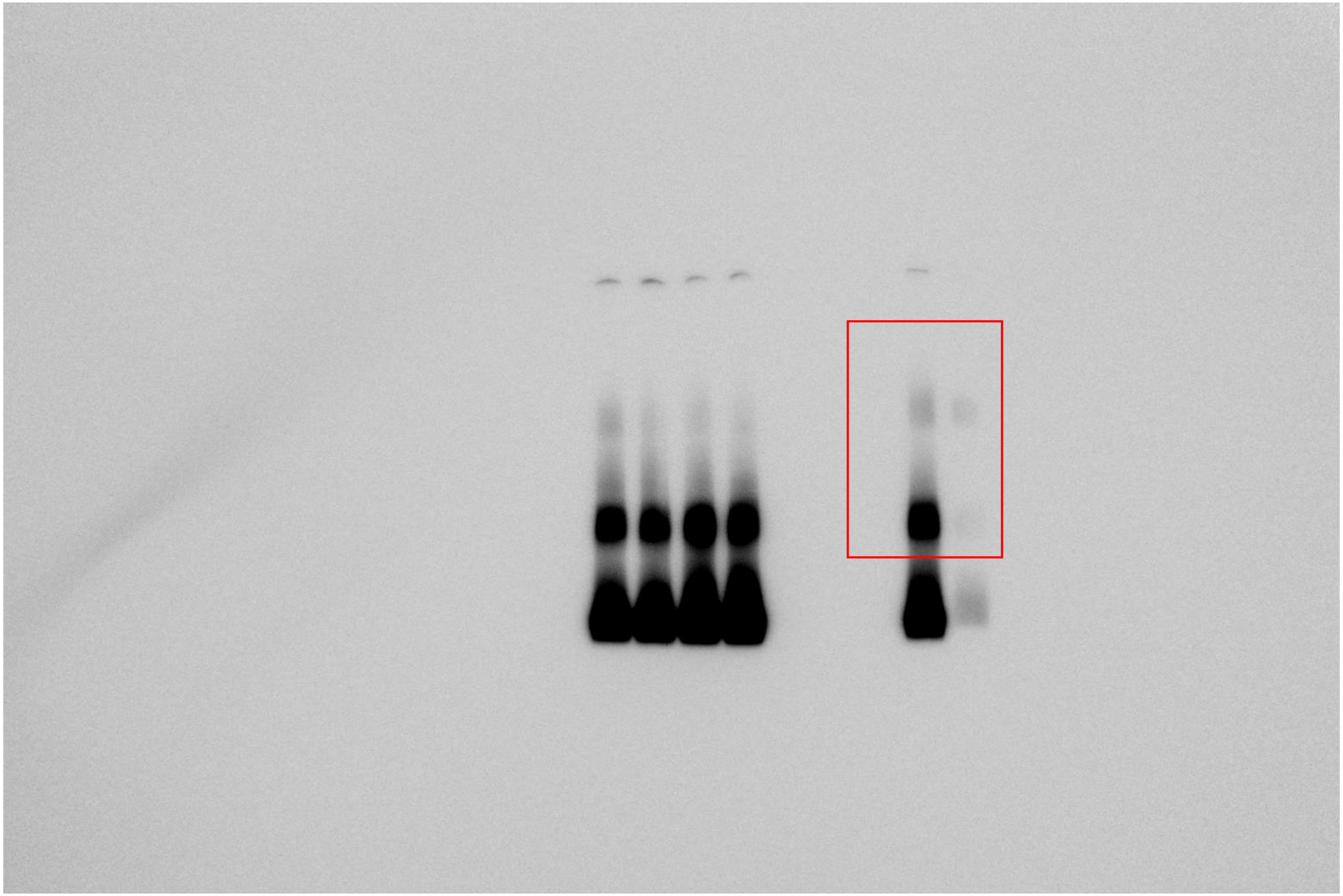

Full unedited blot for Figure 3D (GFP/Gold stain)

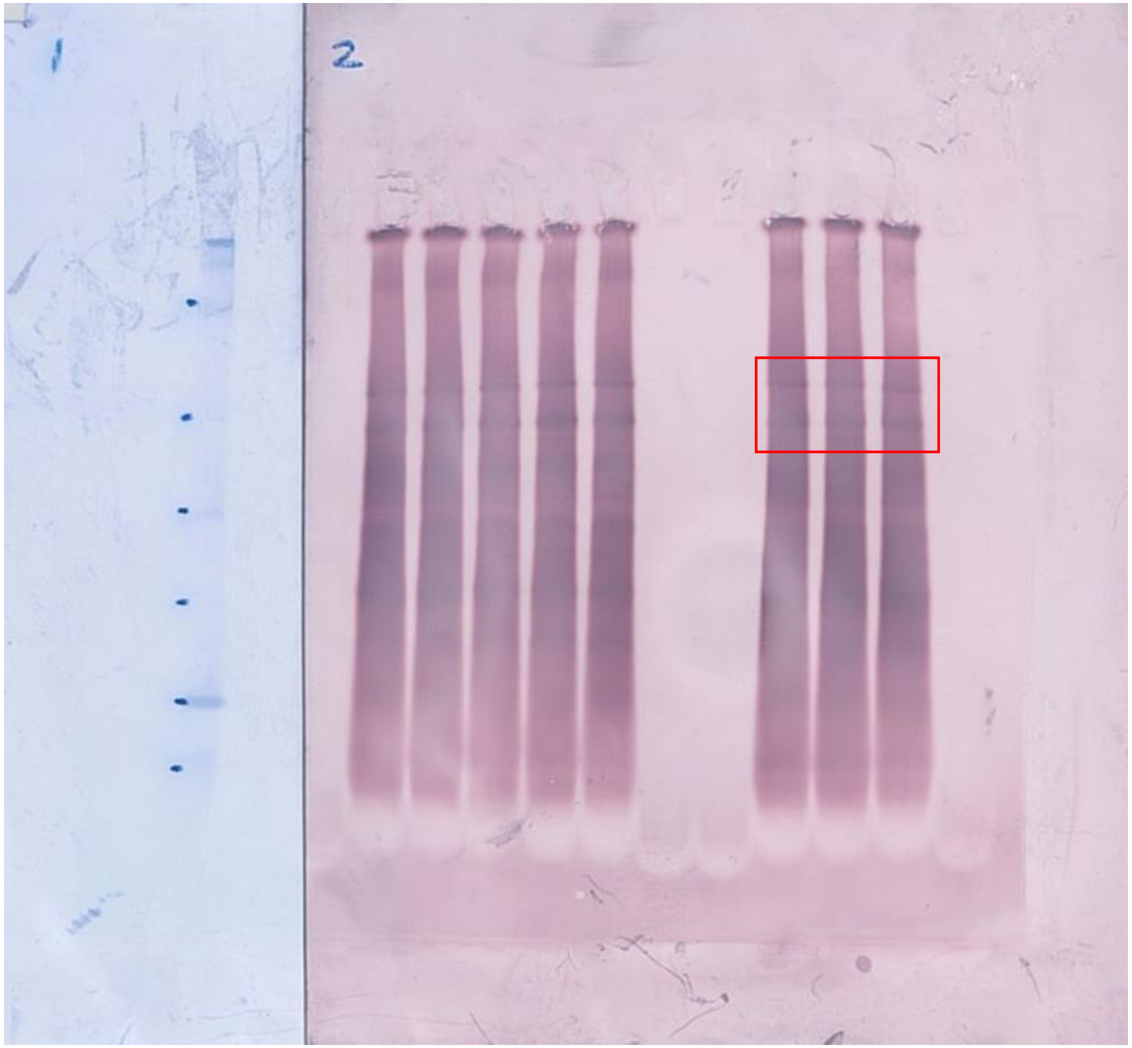

Full unedited blot for Figure 3F (GFP/CBB stain)

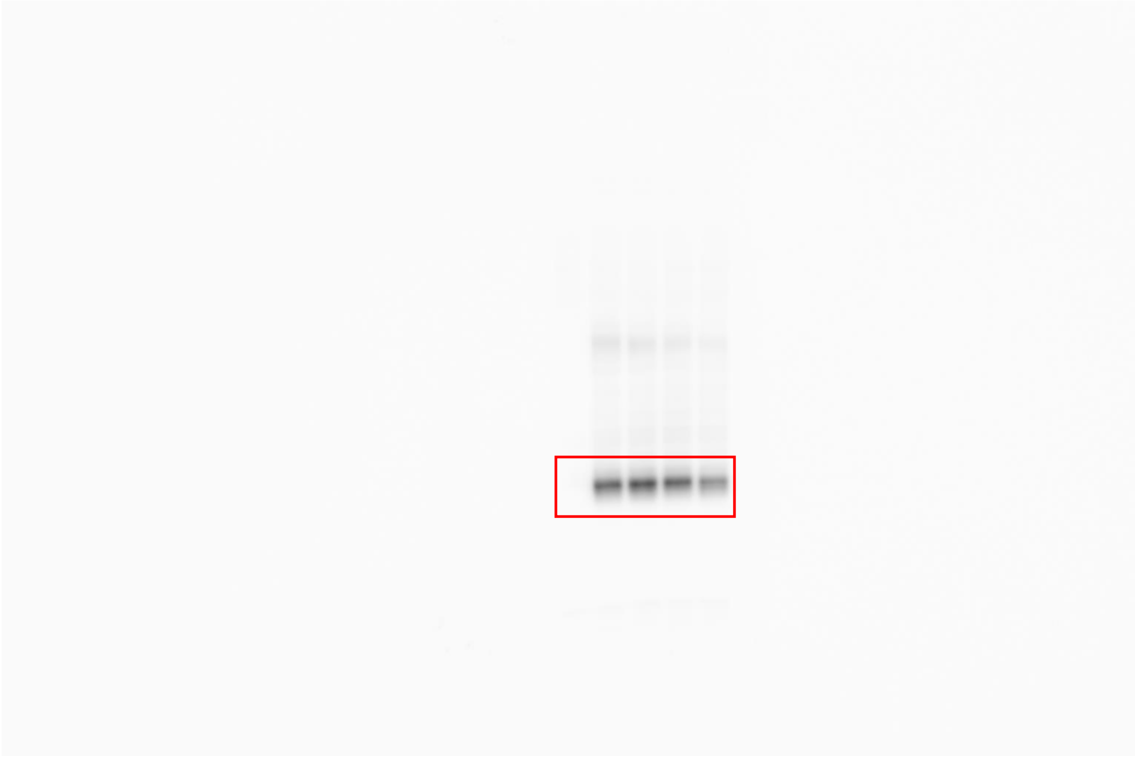

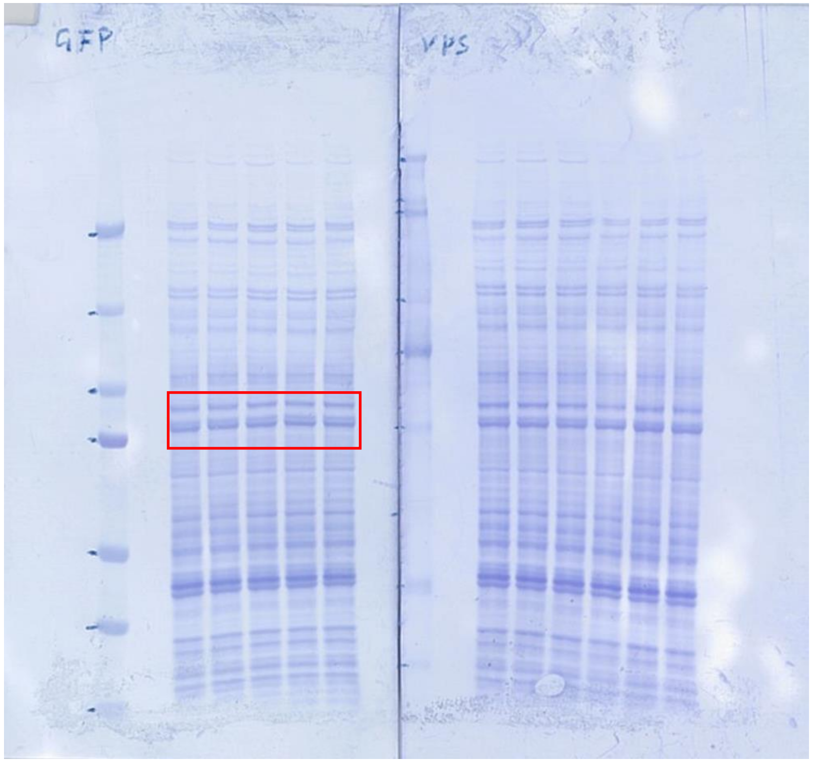

Full unedited blot for Figure 3G (BN-PAGE)

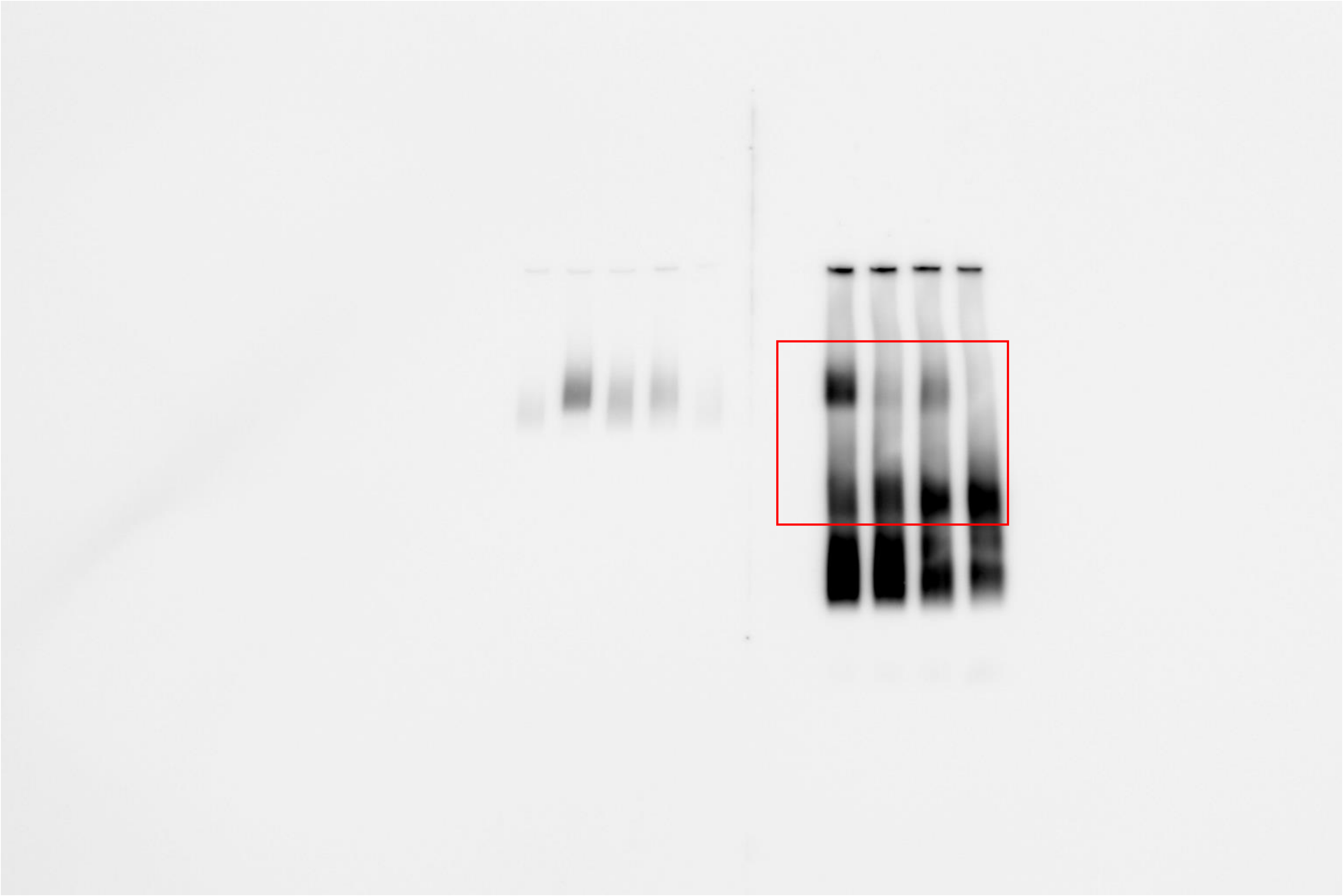

Full unedited blot for Figure 3G (BN-PAGE/Gold)

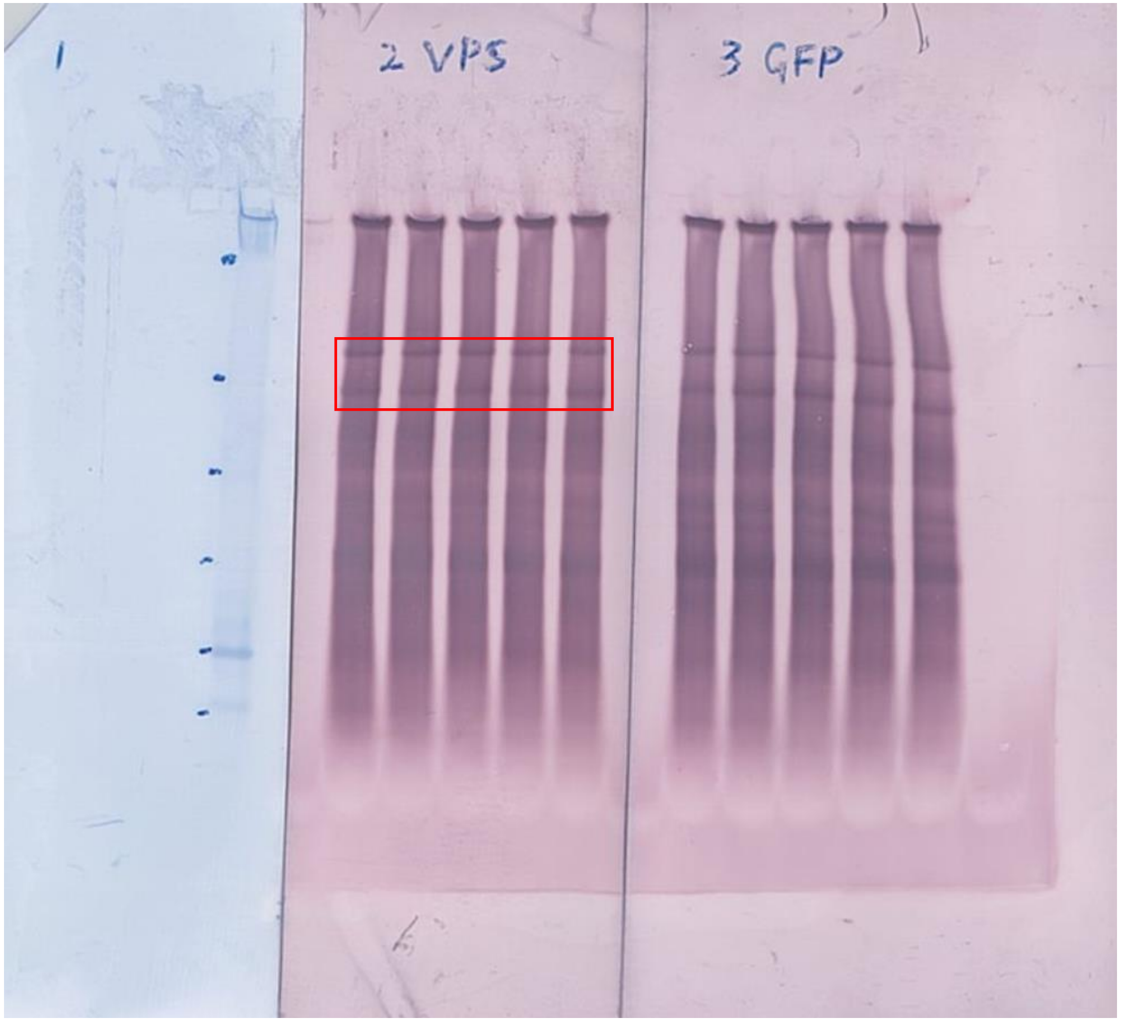

Full unedited blot for Figure 3H (BN-PAGE/Gold stain)

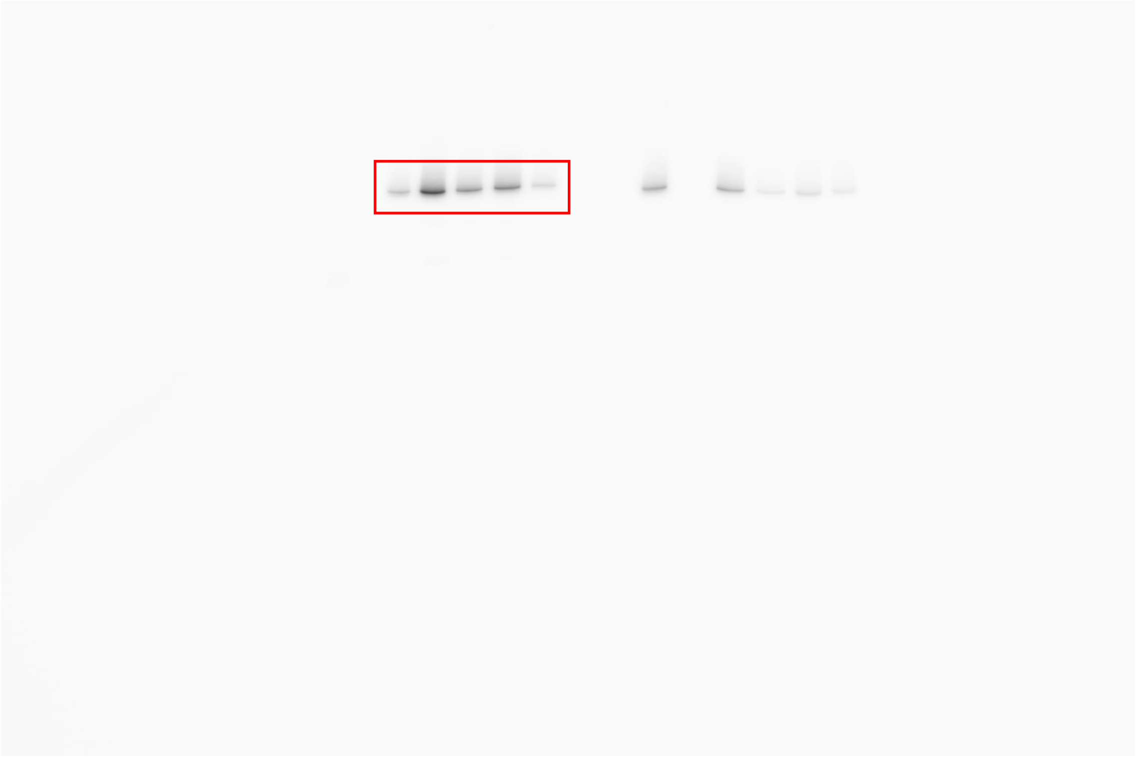

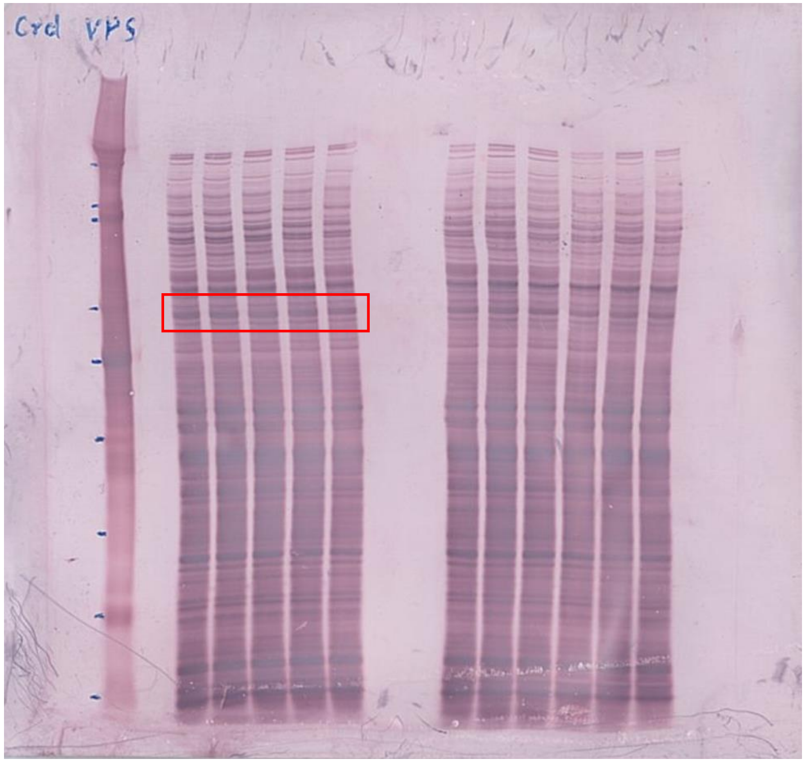

Full unedited blot for Figure 3I (BN-PAGE)

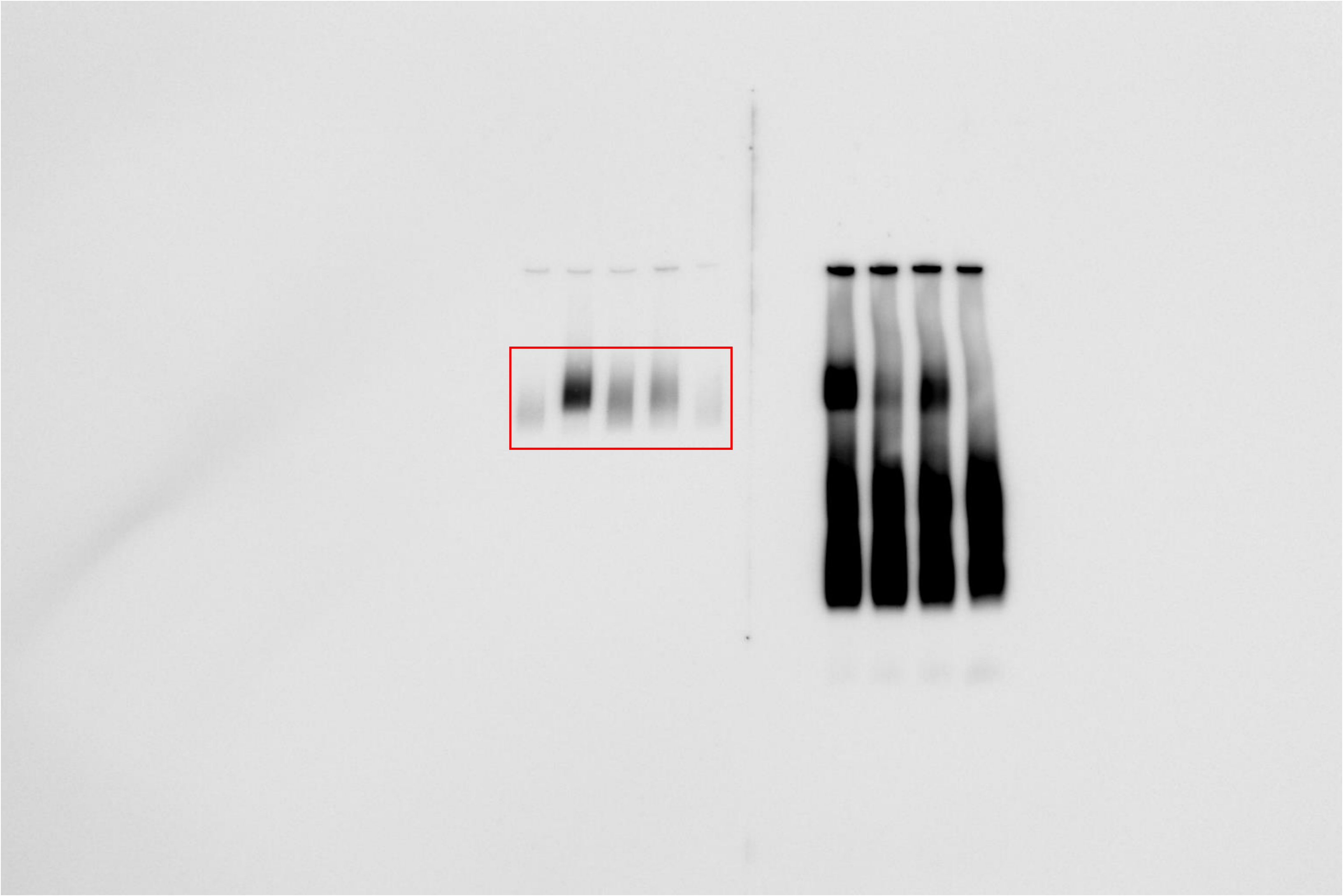

Full unedited blot for Figure 4A (SDS-PAGE)

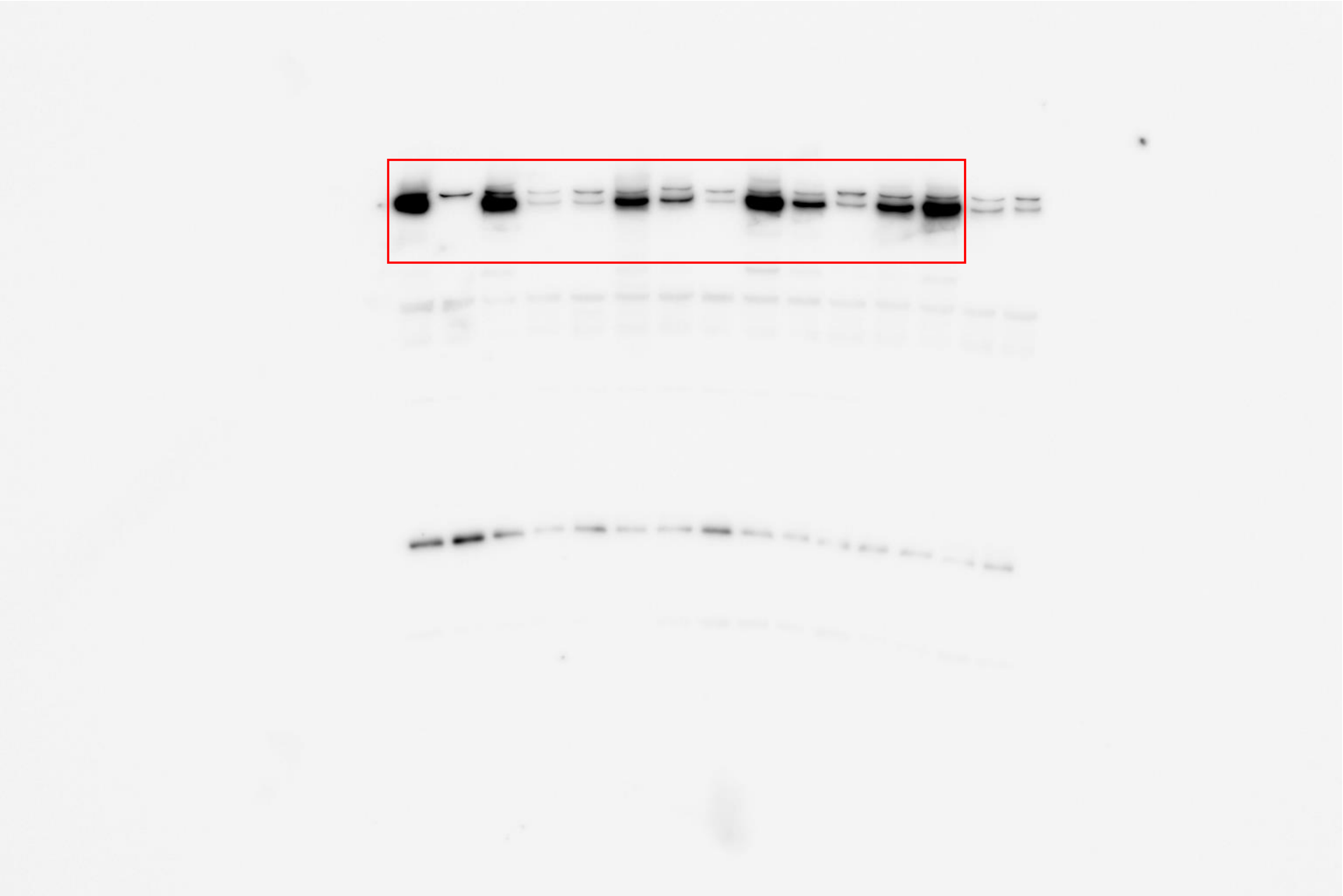

Full unedited blot for Figure 4A (SDS-PAGE/CBB stain)

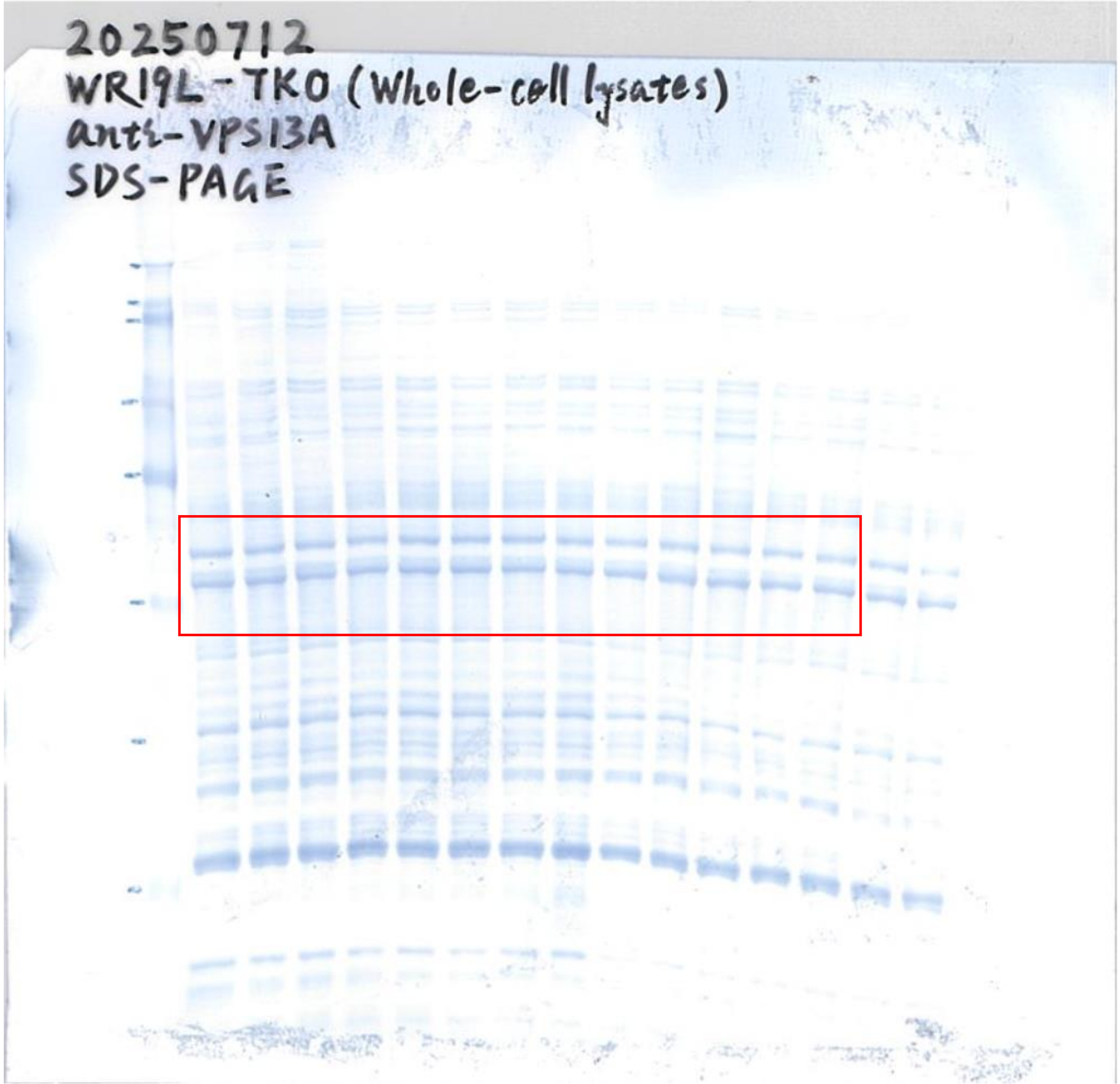

Full unedited blot for Figure 4D (VPS13A)

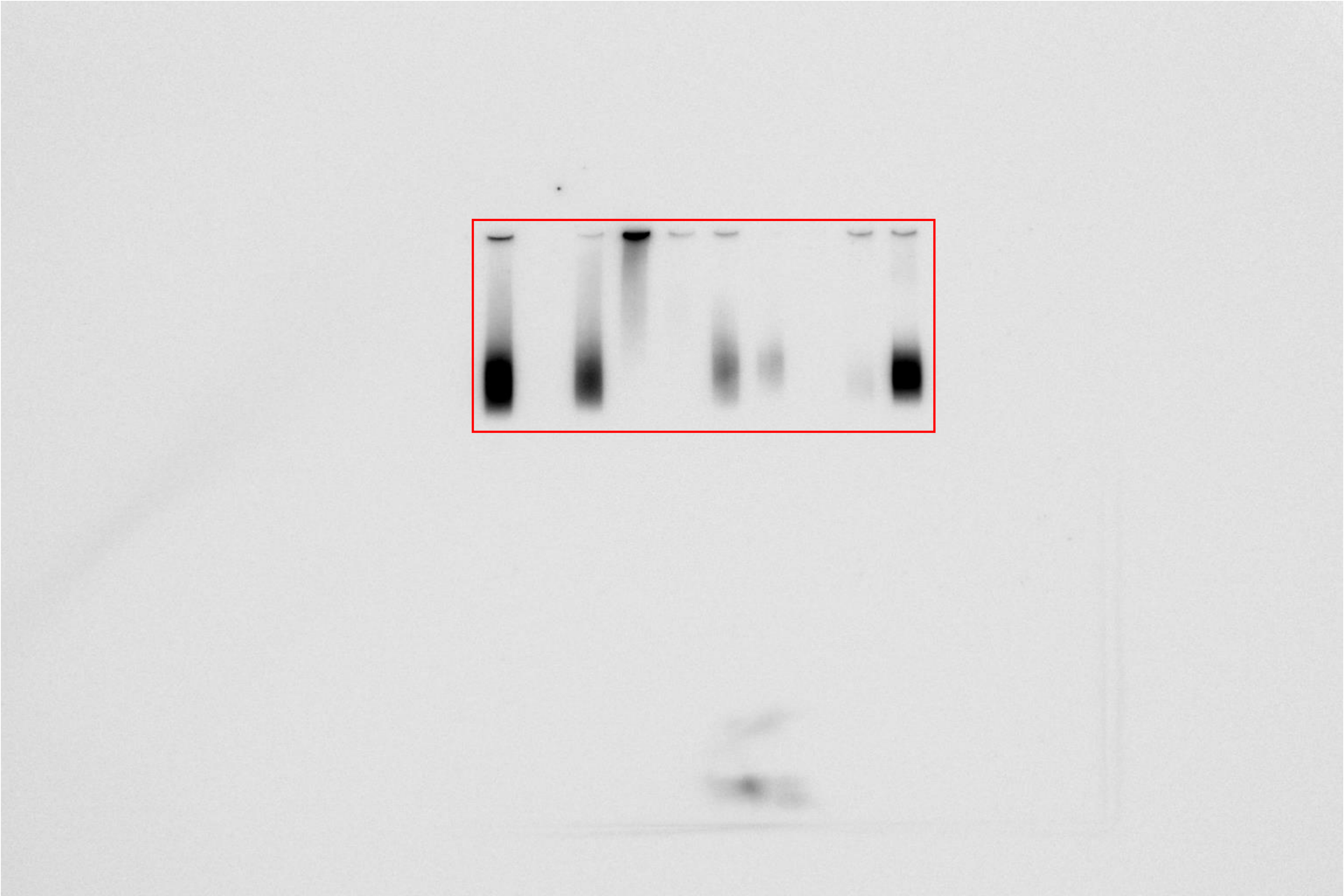

Full unedited blot for Figure 4D (VPS13A/Gold stain)

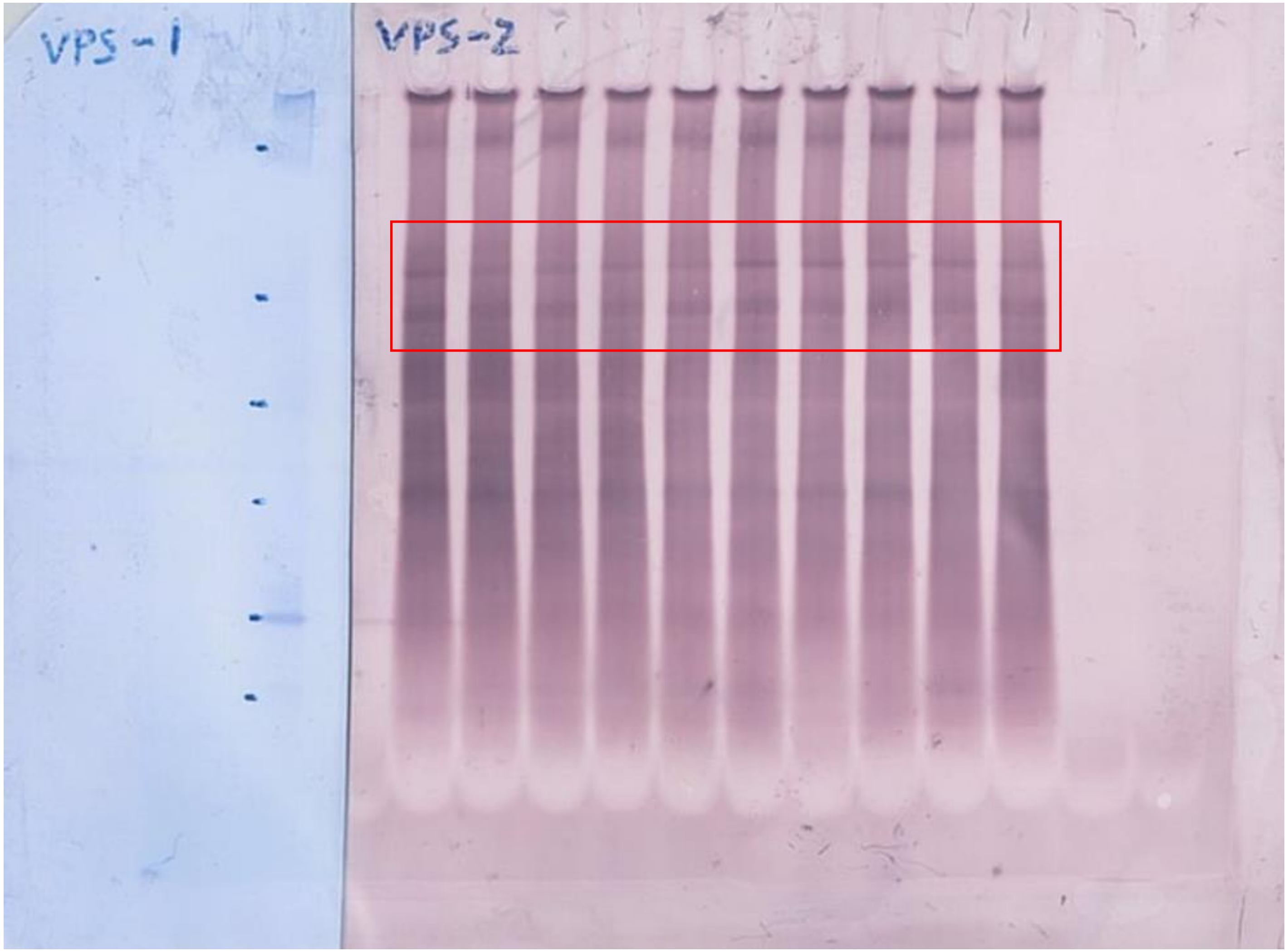

Full unedited blot for Figure 4D (XK)

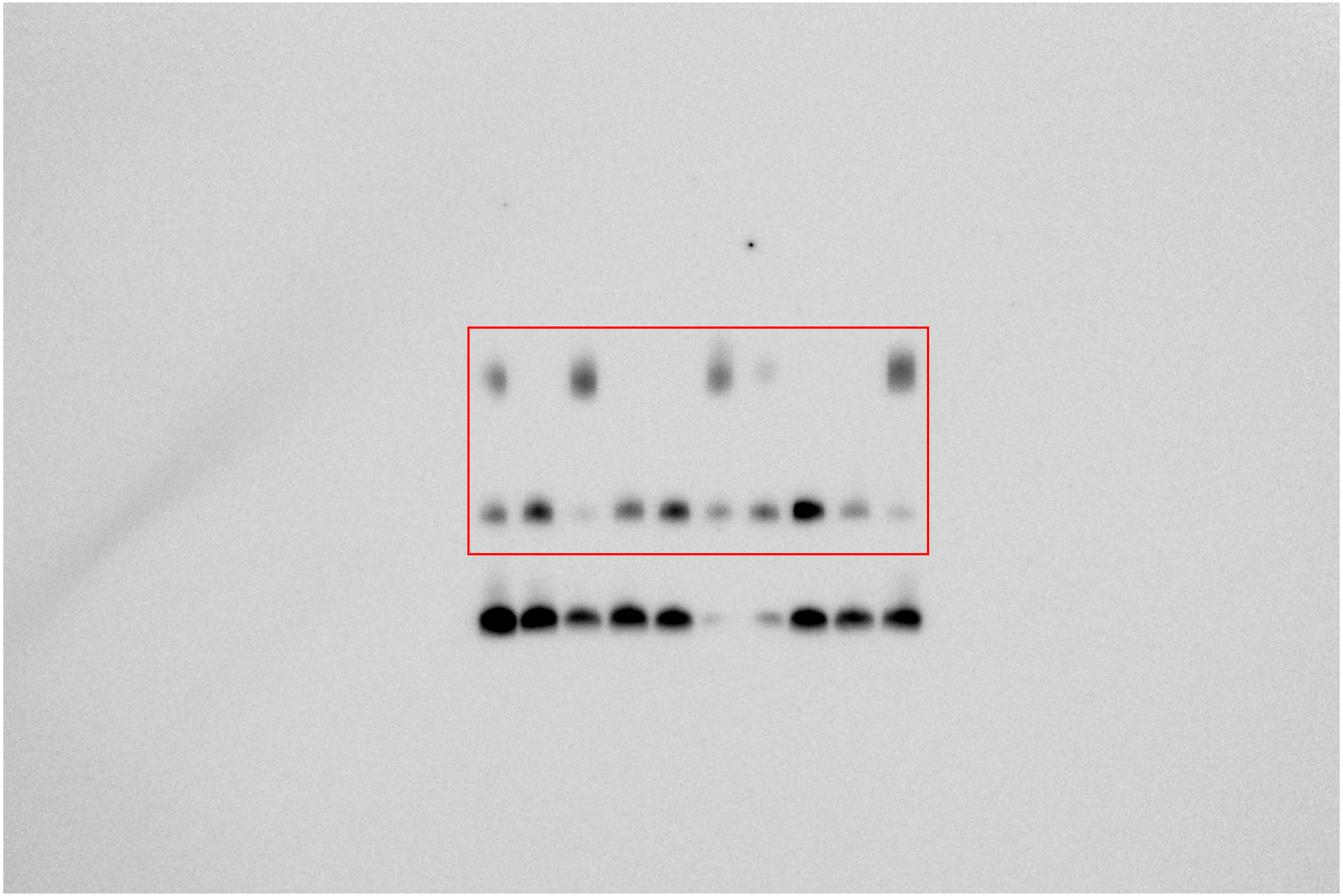

Full unedited blot for Figure 4D (XK/Gold stain)

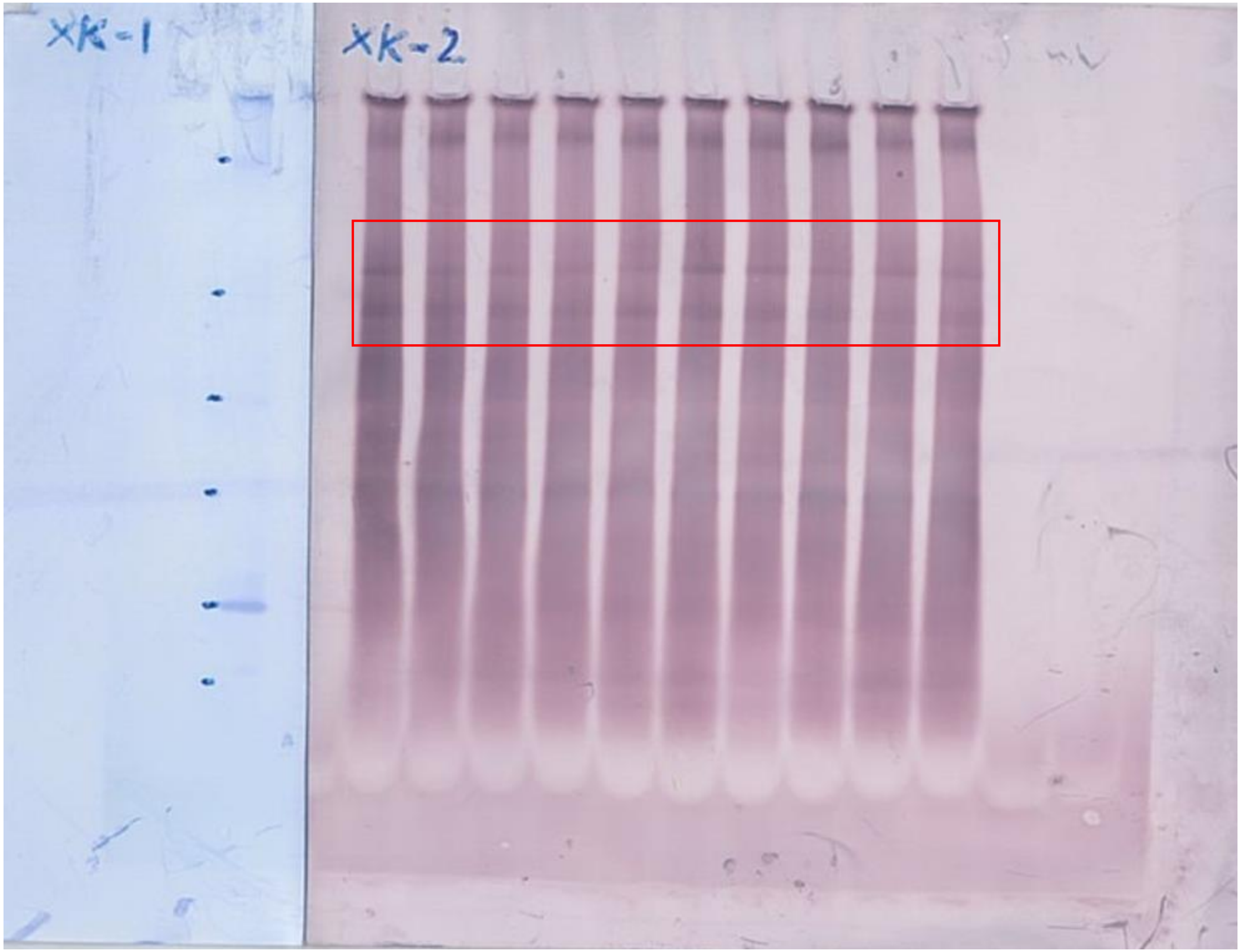

Supplement: Unedited blot and gel images [file jci-136-200890-s145.pdf]
